# Expanding agroforestry can increase nitrate retention and mitigate the global impact of a leaky nitrogen cycle in croplands

**DOI:** 10.1038/s43016-022-00657-x

**Published:** 2022-12-28

**Authors:** Ahmed S. Elrys, Yves Uwiragiye, Yanhui Zhang, Mohamed K. Abdel-Fattah, Zhao-xiong Chen, Hui-min Zhang, Lei Meng, Jing Wang, Tong-bin Zhu, Yi Cheng, Jin-bo Zhang, Zu-cong Cai, Scott X. Chang, Christoph Müller

**Affiliations:** 1 School of Geography, Nanjing Normal University, Nanjing, China; 2 College of Tropical Crops, Hainan University, Haikou, China; 3 Soil Science Department, Faculty of Agriculture, Zagazig University, Zagazig, Egypt; 4 College of Natural Resources and Environment, Northwest A&F University, Yangling, China; 5 Department of Agriculture, Faculty of Agriculture, Environmental Management and Renewable Energy, University of Technology and Arts of Byumba, Byumba, Rwanda; 6 Co-Innovation Center for Sustainable Forestry in Southern China, Nanjing Forestry University, Nanjing, China; 7 Key Laboratory of Karst Dynamics, MLR & Guangxi, Institute of Karst Geology, Chinese Academy of Geological Sciences, Guilin, China; 8 Jiangsu Center for Collaborative Innovation in Geographical Information Resource Development and Application, Nanjing, China; 9 Key Laboratory of Virtual Geographic Environment (Nanjing Normal University), Ministry of Education, Nanjing, China; 10 Department of Renewable Resources, University of Alberta, Edmonton, Alberta Canada; 11 Institute of Plant Ecology, Justus Liebig University Giessen, Giessen, Germany; 12 School of Biology and Environmental Science and Earth Institute, University College Dublin, Dublin, Ireland

**Keywords:** Element cycles, Environmental impact

## Abstract

The internal soil nitrogen (N) cycle supplies N to plants and microorganisms but may induce N pollution in the environment. Understanding the variability of gross N cycling rates resulting from the global spatial heterogeneity of climatic and edaphic variables is essential for estimating the potential risk of N loss. Here we compiled 4,032 observations from 398 published ^15^N pool dilution and tracing studies to analyse the interactions between soil internal potential N cycling and environmental effects. We observed that the global potential N cycle changes from a conservative cycle in forests to a less conservative one in grasslands and a leaky one in croplands. Structural equation modelling revealed that soil properties (soil pH, total N and carbon-to-N ratio) were more important than the climate factors in shaping the internal potential N cycle, but different patterns in the potential N cycle of terrestrial ecosystems across climatic zones were also determined. The high spatial variations in the global soil potential N cycle suggest that shifting cropland systems towards agroforestry systems can be a solution to improve N conservation.

## Abstract (web summary)

The spatial patterns of global soil potential nitrogen cycling were characterized using 4,032 observations from 398 published studies. The global soil nitrogen cycle shifts from conservative in forests to leaky in croplands, highlighting the importance of forests in the global nitrogen cycle and the need for further insights on nitrate retention in croplands.

## Main

Reactive nitrogen (N) supplies N to soil microorganisms and plants but has a negative impact on the environment by affecting the quality of air and water, which in turn affects human health^1^. We thus need to maximize the benefits of reactive N while minimizing its negative impact on the environment^1^. The fate of soil N is affected by the rate of N fluxes and by the chemical form of N^2^, among a large number of other factors. Soil gross N cycling rates provide an understanding of the internal N cycle. A process-based understanding of global gross N transformations remains paramount to explaining how the internal soil N cycle contributes to sustained N losses from terrestrial ecosystems. Given the importance of soil gross N cycling rates for estimating the potential risk of N loss, it is critical to understand the variability of soil gross N cycling rates resulting from the global spatial heterogeneity of climatic and edaphic variables. However, our understanding of the global spatial variations of soil gross N transformation rates is still insufficient. Conceptual frameworks and empirical studies have been suggested during the past few decades to characterize the soil N cycle. For example, the conceptual model of Davidson et al.^3^ suggests that soil where nitrate (NO_3_^−^) dominates over ammonium (NH_4_^+^) has excess N and a ‘leaky’ N cycle (that is, high NO_3_^−^ losses through denitrification or leaching), whereas soil where NH_4_^+^ dominates over NO_3_^−^ is characterized by a ‘conservative’ N cycle. Experimentally, Corre et al.^4^ found a conservative N cycle in boreal forests, where soil immobilization rates of NO_3_^−^ ($I_{{\mathrm{NO}}_3}$, the conversion of NO_3_^−^ into organic N) and NH_4_^+^ ($I_{{\mathrm{NH}}_4}$, the conversion of NH_4_^+^ into organic N) were comparable to rates of gross nitrification (GN, the microbial oxidation of organic N or NH_4_^+^ to NO_3_^−^) and gross N mineralization (GNM, the conversion of organic N into inorganic N), respectively. However, in tropical forest soils, a leaky N cycle has been observed where GNM and GN are greater than $I_{{\mathrm{NH}}_4}$ and $I_{{\mathrm{NO}}_3}$, respectively^5^. In temperate grasslands, a leaky N cycle was observed in China, whereas a conservative N cycle was observed in other regions^6^. Croplands in the different regions are usually also characterized by a leaky N cycle^2,7^. It is unlikely that a general pattern will emerge from these conceptual frameworks and individual experiments that can be applied to a broad range of ecosystems. However, the findings of the individual experiments can be pooled to show a general tendency of ecosystem N cycling patterns. So far, global gross N transformation rates have not been assessed to explain the pattern of soil internal N cycling and its contribution to potential N losses in different ecosystem types. Furthermore, previous global-scale studies reported that soil gross N cycling rates are mainly driven by a combination of soil attributes and climate^8,9^, but these studies neglected the connection between gross N cycling rates. The last data synthesis on these processes dates back almost 20 years^8^ and did not draw firm conclusions about the global pattern of the soil internal N cycle due to the lack of data. There is an urgent need for a global synthesis to clarify how ecosystem-wide, land use, edaphic and climatic factors influence the internal soil N cycle, taking into account the relationship between gross N transformation rates.

To fill these knowledge gaps, we compiled 4,032 observations from 398 published ^15^N pool dilution and tracing studies (Supplementary References and Supplementary Data 1) incorporating gross N cycling rate data across various ecosystems (Supplementary Fig. 1a,b) to characterize the spatial patterns of global soil N cycling. We also analysed the impacts of soil and climate attributes and their interactions on controlling global soil gross N cycling rates, as well as the relationship between gross N cycling rates. Our synthesis aimed to answer three questions. First, what are the global patterns and spatial variations of soil gross N cycling rates, and do they differ across terrestrial ecosystems and climatic zones? Second, how do soil and climate variables interact with gross N cycling rates globally, and what is the connection between gross N cycling rates? Third, what are the implications of the above relationships for the spatial variations of the global soil N cycle? To answer these questions, we first calculated the average gross N transformation rates across ecosystem types and analysed global-scale patterns in the data (Supplementary Tables 1–10). We then predicted the distribution of soil gross N transformation rates globally by five machine learning models using a global database of soil and climatic variables (Supplementary Figs. 2a–c and 3a–h). Next, we conducted structural equation modelling (SEM) to estimate the factors that directly and indirectly control soil N cycling. Finally, we calculated the ratios of gross autotrophic nitrification (GAN, the microbial oxidation of NH_4_^+^ to NO_3_^−^) to $I_{{\mathrm{NH}}_4}$ and of soil NO_3_^−^ to NH_4_^+^, and we used mixed-effects meta-regression models to investigate the main factors affecting these ratios. These ratios are utilized as indicators of the potential risk of N losses.

## Results and discussion

In our dataset, most incubation periods for gross N transformation rates ranged from 24 to 48 h, because gross N rate estimates based on ^15^N isotopic pool dilution after a 48 h incubation can lead to inconsistent estimates^8,10^. Although many studies suggested an incubation period of 24 to 48 h to minimize the effect of remineralization on computed GNM^11^, other studies suggested that GNM is overestimated during short incubation periods^12^. Estimates of soil gross N cycling rates in our analysis should therefore be interpreted with caution. Moreover, most of our data were based on laboratory studies, which do not necessarily reflect the in situ conditions of soil N cycling^8,13–15^. Hence, we recognize that a number of the soil N cycling rates used in our study are possibly more in line with potential rates, a circumstance that also applies to all other studies of this kind. However, to avoid further inconsistencies, we do not use the term ‘potential’ here either, but we point out that the data should be interpreted with the appropriate caution.

### Global patterns of internal N cycling

**Fig. 1:**
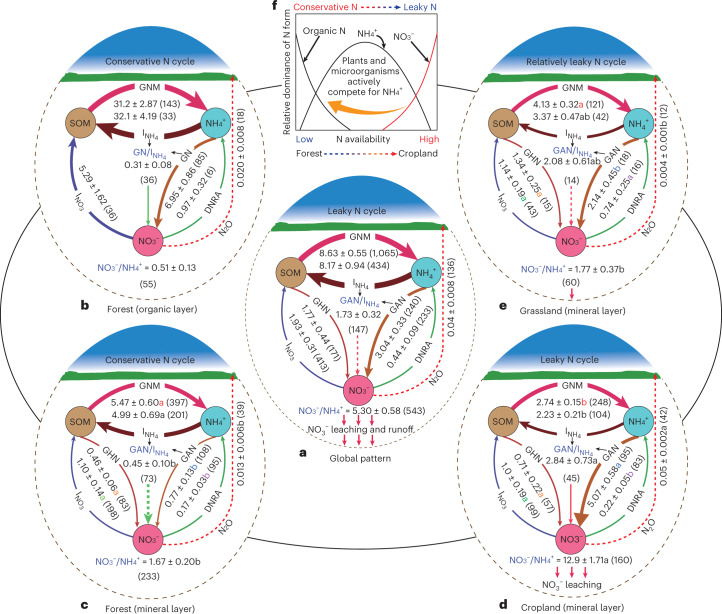
Changes of gross N cycling and N_2_O emission rates (means ± s.e.; mg N kg^−1^ d^−1^) in terrestrial ecosystems and two unique soil layers in forest ecosystems and in the mineral soil in grasslands and croplands. **a**, Pattern of global soil gross N cycling and N_2_O emission rates. **b**, Pattern of soil gross N cycling and N_2_O emission rates in the organic soil in forests. **c**–**e**, Patterns of gross N cycling rates in the mineral soil in forests (**c**), croplands (**d**) and grasslands (**e**). **f**, Conceptual diagram of global soil N cycle under different land uses. Differences in GNM (*P* < 0.0001), $I_{{\mathrm{NH}}_4}$ (*P* = 0.045), $I_{{\mathrm{NO}}_3}$ (*P* = 0.408), GAN (*P* < 0.0001), GHN (*P* = 0.393), DNRA (*P* = 0.004) and N_2_O emission (*P* = 0.01) rates among mineral soil horizons of forests, croplands and grasslands were tested using one-way analysis of variance with least significant differences. The different letters next to the numbers indicate significant differences in gross N transformation and N_2_O emission rates across terrestrial ecosystems at *P* < 0.05, while the values in parentheses are the number of observations. The *P* values were obtained by two-tailed tests. The comparisons among terrestrial ecosystems were here confined to mineral soil horizons. SOM, soil organic matter. Source data

The global averages (±standard errors) of GNM, GAN, gross heterotrophic nitrification (GHN, the microbial oxidation of organic N to NO_3_^−^), $I_{{\mathrm{NO}}_3}$, $I_{{\mathrm{NH}}_4}$ and dissimilatory nitrate reduction to ammonium (DNRA) were 8.63 ± 0.55, 3.04 ± 0.33, 1.77 ± 0.44, 1.93 ± 0.31, 8.17 ± 0.94 and 0.44 ± 0.09 mg N kg^–1^ d^–1^, respectively (Fig. 1a). Soil GN was dominated by GAN (63%; Fig. 1a). GHN was also an important N transformation process, representing 37% and 17% of the total production of NO_3_^−^ and of mineral N, respectively (Fig. 1a). However, recent studies have shown that GHN is stimulated in the presence of plants^16^. Hence, since most of the studies included in our analysis were laboratory studies, it can be expected that the fraction of NO_3_^−^ produced via GHN would be higher than what was demonstrated by our study. Soil $I_{{\mathrm{NH}}_4}$ dominated (81%) gross N immobilization rate (GI) and consumed 90% of the total NH_4_^+^ production, manifesting high NH_4_^+^ retention globally (Fig. 1a). This is consistent with previous studies, indicating a preferential microbial uptake of NH_4_^+^ (ref. 17). Soil microorganisms prefer NH_4_^+^ because of the additional energy requirement for $I_{{\mathrm{NO}}_3}$ and NO_3_^−^ reduction and also because NH_4_^+^ can suppress $I_{{\mathrm{NO}}_3}$ (ref. 18). However, most of our results are based on laboratory studies, so this preference may not be absolute but influenced by other factors. Under high plant NH_4_^+^ demand, for example, plants outcompeted microbial NH_4_^+^ acquisition, resulting in a switch towards $I_{{\mathrm{NO}}_3}$ (ref. 16). We also cannot ignore that NO_3_^−^ moves more easily than NH_4_^+^ in soil solution by diffusion and mass flow to the root surface. A recent study found that plants take up less labelled NH_4_^+^ than NO_3_^−^, while soils retain more NH_4_^+^ than NO_3_^−^ (ref. 19). Furthermore, previous studies suggested that sieving stimulates soil $I_{{\mathrm{NH}}_4}$ but inhibits $I_{{\mathrm{NO}}_3}$ as a result of evenly distributing NH_4_^+^, resulting in an underestimation or overestimation of the gross N transformation rates^13^. As laboratory studies are probably limited in capturing the full soil gross N cycling rate dynamics, our global estimates of soil gross N cycling rates should be interpreted with appropriate caution.

**Fig. 2:**
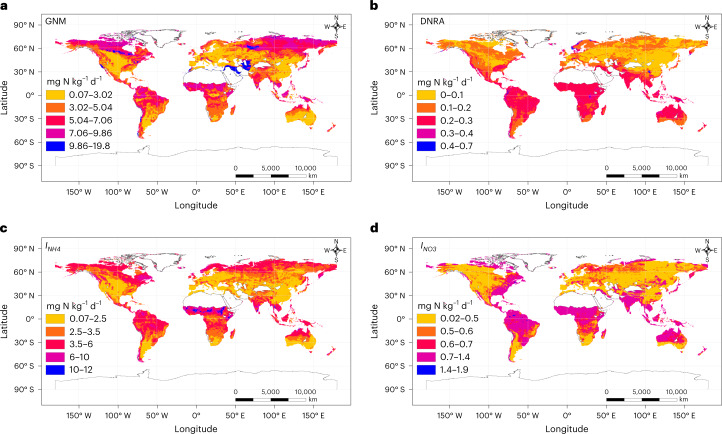
The global spatial variations of soil gross N transformation rates resulting from the global spatial heterogeneity of climatic and edaphic variables. **a**–**d**, The global spatial variations of GNM (**a**), DNRA (**b**), gross ammonium immobilization (**c**) and gross nitrate immobilization (**d**).

The fact that soil NO_3_^−^ is more likely to be lost to the environment indicates the need to maximize the global NO_3_^−^ consumption processes ($I_{{\mathrm{NO}}_3}$ and DNRA). Although previous studies have demonstrated that the contribution of $I_{{\mathrm{NO}}_3}$ to GI was negligible^20^, we found that $I_{{\mathrm{NO}}_3}$ represents 19% of global GI and 40% of total NO_3_^–^ production (Fig. 1a). $I_{{\mathrm{NO}}_3}$ in the soil temporarily converts NO_3_^−^-N into microbial biomass, where it can later be converted into stable organic N or remineralized, decreasing the risk of N loss from the soil^21^. We also found that DNRA accounts for 18.5% of the global NO_3_^−^ consumption (Fig. 1a). Although we noticed that the processes of $I_{{\mathrm{NO}}_3}$ and DNRA occur, they are still low and consume less than 50% of the global NO_3_^−^ production, demonstrating a lower global NO_3_^−^ retention. However, we cannot disregard recent studies indicating the critical role of plant root exudates in stimulating DNRA in soil^22^, suggesting that gross N cycling rates based on laboratory studies in our analysis may be different in the presence of plants. As a result of low NO_3_^−^ retention, high ratios of soil NO_3_^−^ to NH_4_^+^ (5.30) and GAN to $I_{{\mathrm{NH}}_4}$ (1.73) were observed at the global scale, indicating a leaky N cycle (Fig. 1a), and thus there is a high potential risk of N loss^2^. A relatively high average nitrous oxide (N_2_O) emission rate (40 ± 8.0 µg N kg^−1^ d^−1^, *n* = 136) was observed globally (Fig. 1a). However, we observed high spatial variations in the global N cycle (Fig. 2) as its pattern changes from a conservative cycle in forests to a less conservative one in grasslands and a leaky one in croplands (Fig. 1), as discussed below.

#### Patterns of internal N cycling in croplands

**Fig. 3:**
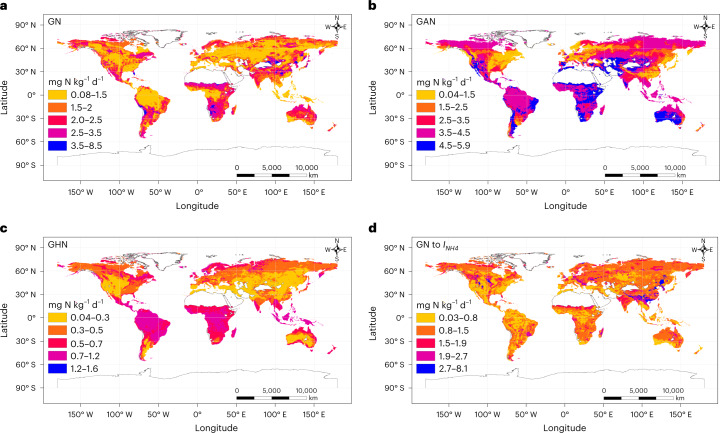
The global spatial variations of soil gross N transformation rates resulting from the global spatial heterogeneity of climatic and edaphic variables. **a**–**d**, The global spatial variations of GN (**a**), GAN (**b**), GHN (**c**) and the ratio of GN to gross ammonium immobilization (**d**).

A decoupled N cycle was observed in croplands: $I_{{\mathrm{NH}}_4}$ rates were somewhat lower than GNM rates, GN rates were six times those of $I_{{\mathrm{NO}}_3}$, the GAN-to-$I_{{\mathrm{NH}}_4}$ ratio was 2.84 ± 0.73 and the NO_3_^−^-to-NH_4_^+^ ratio was 12.9 ± 1.71, indicating a leaky soil N cycle (Fig. 1d), which is in line with previous findings^23^. Soils with a low GAN-to-$I_{{\mathrm{NH}}_4}$ or a low soil NO_3_^−^-to-NH_4_^+^ ratio have a lower potential for N losses than those with high ratios^2^. High ratios of GAN to $I_{{\mathrm{NH}}_4}$ and NO_3_^−^ to NH_4_^+^ in croplands resulted in high N_2_O emissions (Fig. 1d and Supplementary Fig. 4). Our study revealed that GAN, GN, and the ratios of GAN to $I_{{\mathrm{NH}}_4}$ and NO_3_^−^ to NH_4_^+^ in grasslands and forests were significantly lower than those in croplands (Fig. 1c–e), which is in line with previous studies^8,9,23,24^. Agricultural practices result in different soil pH conditions, leading to a different function and structure of the community of soil microorganisms. For example, the high rate of GN in croplands may be associated with high nitrifier activity^24^. Generally, ammonia-oxidizing bacteria (a type of nitrifying bacteria that oxidizes ammonia to NO_3_^−^) cannot grow in soil with pH less than 5.0–5.5 (ref. 25). In our dataset, croplands have an average pH of 6.26, conditions that favour ammonia-oxidizing bacteria^25^. Long-term N supply would promote GN through enhancing the abundance and activity of ammonia-oxidizing bacteria^26^. However, agricultural practices increase soil aeration by damaging soil structure, which accelerates carbon (C) decomposition^27^. Additionally, high rates of mineral N additions block the production of humus-degrading enzymes by soil microorganisms and thus inhibit GNM^4^ and ultimately GI. Among the climatic zones, the highest rates of GN and the highest ratios of GAN to $I_{{\mathrm{NH}}_4}$ and NO_3_^−^ to NH_4_^+^ were found in humid subtropical croplands (Supplementary Figs. 5 and 6). In support of this, our global predictions revealed higher rates of GAN and GN as well as higher ratios of GN to $I_{{\mathrm{NH}}_4}$ in croplands in tropical and subtropical regions (Fig. 3a,b,d).

#### Patterns of internal N cycling in natural ecosystems

**Fig. 4:**
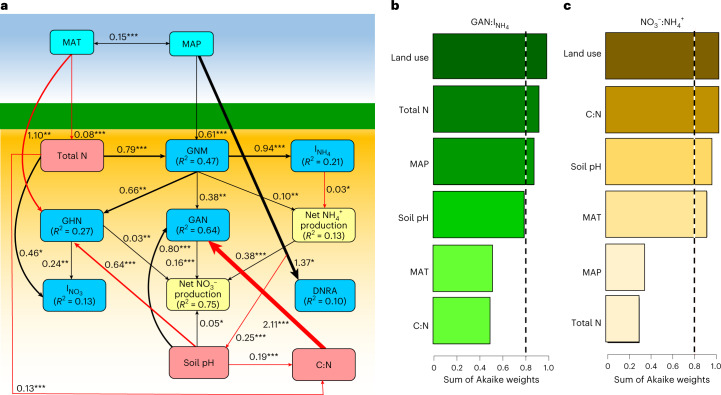
Climatic and edaphic variables that affect soil gross N transformation rates globally. **a**, SEM revealing the influences of MAP, MAT, soil pH, soil total N and soil C/N ratio on gross N transformation rates (GNM, $I_{{\mathrm{NH}}_4}$*,* $I_{{\mathrm{NO}}_3}$, GAN, GHN and DNRA) and net NH_4_^+^ and NO_3_^−^ production. The black and red arrows indicate significant positive and negative relationships, respectively, where the significance level was set at *α* = 0.05. *P< 0.05; **P < 0.01; ***P < 0.001, based on two-tailed tests. The values beside the arrows are standardized coefficients. *R*^2^ refers to the proportion of the variance explained by endogenous variables. **b**,**c**, Model-averaged importance of the predictors of the effect of the variable on the ratios of GAN to $I_{{\mathrm{NH}}_4}$ (**b**) and soil NO_3_^−^ to NH_4_^+^ (**c**). The importance is based on the sum of Akaike weights derived from the model selection process using Akaike’s information criterion corrected for small samples. The cut-off is set at 0.8 (dashed line) to differentiate between important and unimportant predictors. Source data

We found a coupled N cycle between the organic and mineral layers of forest soils: $I_{{\mathrm{NH}}_4}$ and $I_{{\mathrm{NO}}_3}$ rates were comparable to GNM and GN rates, the ratios of GN to $I_{{\mathrm{NH}}_4}$ and of GAN to $I_{{\mathrm{NH}}_4}$ were 0.31 ± 0.08 and 0.45 ± 0.10 in the organic and mineral layers, respectively, and the ratios of NO_3_^−^ to NH_4_^+^ were 0.51 ± 0.13 and 1.67 ± 0.20 in the organic and mineral layers, respectively, manifesting a conservative soil N cycle (Fig. 1b,c), which is consistent with earlier findings^23^. GNM and $I_{{\mathrm{NH}}_4}$ in croplands were significantly lower than those in grasslands and forests (Fig. 1c–e), which is again consistent with previous studies^8,9,23,24^. Our global predictions are in line with our observed patterns of gross N transformation rates across ecosystem types (Fig. 2a,c,d), as forest and grassland soils mostly had high rates of GNM and $I_{{\mathrm{NH}}_4}$ across various climatic zones, and most had high $I_{{\mathrm{NO}}_3}$ rates in tropical and subtropical zones. Former regional-scale to global-scale studies reported that GNM, $I_{{\mathrm{NH}}_4}$ and $I_{{\mathrm{NO}}_3}$ were best explained by soil microbial biomass^8,9^, which is consistent with our findings (Supplementary Tables 1, 6 and 7). Soil total C and N, which are key sources of energy for soil microorganisms, were higher in grasslands and forests than in croplands, thus promoting soil microbial biomass^24^. In support of this, the higher availability of soil substrates to microorganisms in forest organic soil horizons enhances microbial activity and ultimately GNM, $I_{{\mathrm{NH}}_4}$ and $I_{{\mathrm{NO}}_3}$ (*P* < 0.01; Fig. 1b and Supplementary Fig. 7). However, due to the limited availability of substrates in mineral layers of forest soils, microbial activities were restricted^9^, and thus gross N transformation rates in mineral soil layers also decreased (Fig. 1c). In contrast, significantly higher soil C/N ratios in forests increase the microbial N demand and thus reduce the substrate (NH_4_^+^) availability for nitrification, which explains the observed lower rates of GAN and GN (Fig. 4a and Supplementary Tables 2 and 4). Moreover, the rapid recycling of NH_4_^+^ in forests may leave little chance for nitrifiers to compete for available NH_4_^+^. In our dataset, forests had an average pH of 4.86, so nitrification in forest soils was probably limited by low pH^25^. This also may explain why GAN in grasslands was higher than in forests (Fig. 1e), as the average pH of grasslands was 6.17 in our dataset. We thus noted a decoupled N cycle in grasslands; total NO_3_^−^ consumption represents 57% of total NO_3_^−^ production, and the ratios of GAN to $I_{{\mathrm{NH}}_4}$ and NO_3_^−^ to NH_4_^+^ were 2.08 ± 0.61 and 1.77 ± 0.37, respectively, manifesting a leaky N cycle (Fig. 1e). However, the soil N cycle in grasslands was less leaky than that in croplands; the ratios of GAN to $I_{{\mathrm{NH}}_4}$ and NO_3_^−^ to NH_4_^+^ in grasslands were 1.36 and 7.29 times less than those in croplands.

Furthermore, we analysed a subset of data for sites that measured the full N cycle or most variables of soil N processes (Supplementary Data 2) to test whether the number of observations affected the global pattern of the soil N cycle. The results of this subset confirmed our findings that the soil N cycle pattern changes from conservative in forests to leaky in croplands, as indicated by the increasing ratios of GAN to $I_{{\mathrm{NH}}_4}$ and of soil NO_3_^–^ to NH_4_^+^ from 0.48 ± 0.10 and 1.72 ± 0.34 in forests to 2.06 ± 0.35 and 14.2 ± 2.94 in croplands, respectively (Supplementary Fig. 8).

Arctic ecosystems are generally expected to be limited by the availability of nutrients, including N. When soil freezes, microbial activity (which is the main stimulator of GNM^8^) is inhibited as the temperature decreases because the liquid water film, which is a prerequisite for biological activity, is reduced^28^. This reduction in liquid water films prevents soil substrate diffusion and soil microorganism and enzyme activities^29^, ultimately reducing GNM^9^. Moreover, the space of air-filled pores in the soil may decrease as a result of the expansion of water during freezing, causing less oxygen diffusion and the microbial depletion of oxygen remaining in those pores, thereby suppressing aerobic respiration^28^. Hence, the cold climate in the Arctic slows down the activities of decomposers, reducing GNM. The high C/N ratio is also a major reason for the low N mineralization rates in Arctic soils^30^. In contrast, our global predictions showed that GNM rates in the Arctic are higher than in some of the most productive black soils on Earth, which is hard to imagine. However, given that the gross N transformation rates included in our global analysis are often measured under laboratory conditions, it is not surprising that C-rich soils would have higher gross N rates than soils with lower C (for example, boreal forests versus croplands)^8,9^. In contrast to field studies, soil moisture and temperature conditions are precisely controlled in the laboratory, which may affect the activities of the decomposers and ultimately the GNM. For instance, Rustad et al.^31^ found that a temperature increase of 2.4 °C improves soil N mineralization by 46%. In addition, microbial access to substrates is driven by the availability of water in the frozen soil^32^. In dry tundra, the effect of snow depth on the increase of soil N availability was less pronounced than that in moist tundra^33^. Therefore, the difference between field and laboratory flux measurements may be the main reason for the high rate of GNM in the Arctic in our global predictions. Gross N cycling rates in our global analysis should thus be interpreted with caution and need to be validated under field conditions. However, we should not ignore the studies that reported that soil GNM increases with increasing snow depth, which is due to enhanced soil organic C availability and abundance of N mineralization genes^34^. This increase in organic C substrate availability may have resulted from the increased breakdown of soil organic macromolecules or C and N input through microbial cell turnover or killed roots^33^. During winter, deepened snow increases the underlying soil thermal insulation, causing higher soil temperatures^34^. For example, increased snow depth from 30 to 150 cm increased the soil surface temperature by 6 °C^35^, which may enhance soil organic matter decomposition and gross N transformation rates^33,34^. There is therefore an ongoing debate about soil N cycling rates in the Arctic, and there is still an urgent need for more field studies to resolve this controversy.

### Classifying drivers of the global soil internal N cycle

**Fig. 5:**
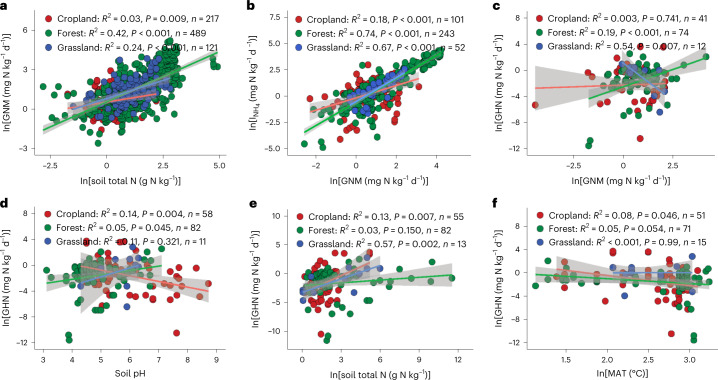
Relationships of gross N transformation rates to each other and to environmental factors across terrestrial ecosystems. **a**, The regression relationship between GNM and soil total N across terrestrial ecosystems. **b**, The regression relationship between $I_{{\mathrm{NH}}_4}$ and GNM across terrestrial ecosystems. **c**, The regression relationship between GHN and GNM across terrestrial ecosystems. **d**, The regression relationship between GHN and soil pH across terrestrial ecosystems. **e**, The regression relationship between GHN and soil total N across terrestrial ecosystems. **f**, The regression relationship between GHN and MAT across terrestrial ecosystems. The solid lines are the slopes, the grey areas indicate the 95% confidence intervals around the regression lines and *n* is the number of observations. Statistical significance was obtained with a two-tailed Student’s *t*-test. Source data

**Fig. 6:**
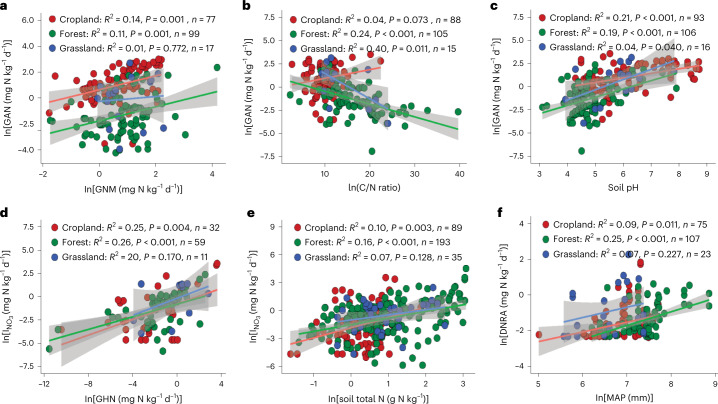
Relationships of gross N transformation rates to each other and to environmental factors across terrestrial ecosystems. **a**, The regression relationship between GAN and GNM across terrestrial ecosystems. **b**, The regression relationship between GAN and soil C/N ratio across terrestrial ecosystems. **c**, Regression between GAN and soil pH across terrestrial ecosystems. **d**, The regression relationship between $I_{{\mathrm{NO}}_3}$ and GHN across terrestrial ecosystems. **e**, The regression relationship between $I_{{\mathrm{NO}}_3}$ and soil total N across terrestrial ecosystems. **f**, The regression relationship between DNRA and MAP across terrestrial ecosystems. The solid lines are the slopes, the grey areas indicate the 95% confidence intervals around the regression lines and *n* is the number of observations. Statistical significance was obtained with a two-tailed Student’s *t*-test. Source data

Total soil N content was the most important factor influencing GNM (Fig. 4a). Soils with a higher total N content typically contain more microbial biomass^9^ and exhibit greater GNM rates^8,9^ (Supplementary Table 1). This relationship between soil total N and GNM is maintained across terrestrial ecosystems and climatic zones (Fig. 5a and Supplementary Fig. 9a). Precipitation can also influence global GNM (Fig. 4a) by altering plant community composition and related litter fall input, which increases soil substrate availability, thus promoting soil microbial biomass^9^. In support of this, the highest rates of GNM were observed in tropical forests (Supplementary Fig. 5a) with high rainfall and abundant soil substrates. We also found that GAN is mainly controlled by soil C/N ratio, soil pH and GNM, with standardized coefficients of −2.11, 0.80 and 0.38, respectively (Fig. 4a and Supplementary Table 4). The requirement of microorganisms for inorganic N increases during organic C decomposition in soils with a high C/N ratio, thus decreasing the substrate NH_4_^+^ for nitrifiers and resulting in a low abundance of ammonia-oxidizing bacteria, which use NH_4_^+^ as a substrate^36^. We found that GAN increased with increasing ammonia-oxidizing bacteria (*R*^2^ = 0.31) and overall bacteria (*R*^2^ = 0.52) abundances (*P* = 0.001; Supplementary Table 4). However, our study showed that soil C/N ratio controls GAN only in natural ecosystems (forests and grasslands) (Fig. 6b) and in all climatic zones except the continental zone (Supplementary Fig. 9c). Free ammonia rather than NH_4_^+^ is the substrate of ammonia-oxidizing bacteria. A higher soil pH shifts the equilibrium between NH_4_^+^ and ammonia towards ammonia, thus increasing ammonia availability and ultimately GAN (Fig. 4a and Supplementary Table 4). This significant and positive influence of soil pH on GAN is maintained across different terrestrial ecosystems (Fig. 6c), but it has been shown only in the continental, humid subtropical and Mediterranean regions (Supplementary Fig. 9d). Although the stimulated effect of GNM on GAN is plausible because the mineralization process is the master producer of NH_4_^+^, which is the main substrate for soil nitrifiers^9^, our study showed that GNM was a controlling factor of GAN in forest and croplands but not in grasslands (Fig. 6a). In addition, GNM and GAN were correlated only in the continental and humid subtropical zones (Supplementary Fig. 9b).

Previous studies suggested that GHN in acidic soils contributes to GN^37^. This is consistent with our SEM, which found that soil pH is a negative factor controlling global GHN (Fig. 4a). Lowering soil pH could enhance soil fungal abundance, which in turn stimulates GHN^37^. There were positive relationships between GHN and the abundance of fungi (*R*^2^ = 0.55, *P* < 0.001) and the fungi-to-bacteria ratio (*R*^2^ = 0.54, *P* = 0.002) at the global scale (Supplementary Table 3). Soil GHN is more closely related to fungal activity due to their lower N demand per unit C and their higher acid tolerance than bacteria^6,37,38^. Soils with high organic C and low pH therefore exhibit relatively higher fungal activity. Our study showed that total soil C is positively associated with GHN (*P* < 0.001) and negatively correlated with GAN (*P* = 0.11; Supplementary Tables 3 and 4). Unexpectedly, this inverse relationship between soil pH and GHN existed only in croplands (Fig. 4d) and in humid subtropical zones (Supplementary Fig. 10f), while GHN increased significantly with increasing soil pH in forests (Fig. 5d). Furthermore, we found positive relationships among GHN, total N and GNM rate (*P* < 0.01; Supplementary Table 3), but our SEM (Fig. 4a) revealed that GHN was more closely related to GNM than to total N, indicating the importance of NH_4_^+^ as a substrate for heterotrophic nitrifiers. Our global predictions also confirmed the importance of NH_4_^+^ as a substrate for heterotrophic nitrifiers, as higher GHN rates were observed in the tropics with higher rates of GNM (Fig. 3c). By examining these relationships in different terrestrial ecosystems, we found that GNM plays a central role in controlling GHN only in forests (Fig. 5c), but soil total N controls GHN in croplands and grasslands (Fig. 5e). In contrast, GHN decreased (*R*^2^ = 0.54, *P* = 0.007, *n* = 12; Fig. 5c) with increasing GNM in grasslands. Our analysis also revealed that mean annual temperature (MAT) was a driving factor of GHN globally (Fig. 4a and Supplementary Fig. 5f), which is consistent with the finding of Liu et al.^39^, who reported that high temperatures decrease GHN. Soil fungi, which control GHN (Supplementary Table 3), are more active than bacteria at lower temperatures^40^. Additionally, our SEM (Fig. 4a) revealed that high temperatures reduce soil total N content, which is a substrate for GHN and GNM. The highest rate of GHN was recorded in the continental climate zone, confirming the negative effect of temperature on GHN (Supplementary Table 3 and Fig. 4a). Our global predictions also revealed high rates of GHN in the continental climate zone (Fig. 3c). However, the effect of MAT on GHN was inconsistent across climatic zones. For example, GHN increased significantly with decreasing MAT in the humid subtropical and Mediterranean regions but with increasing MAT in the continental regions (Supplementary Fig. 9h), suggesting that the effect of temperature on GHN is not universal but has a threshold. The highest average GHN (4.00 ± 2.07 mg N kg^−1^ d^−1^, *n* = 24) in our dataset was recorded when MAT was in the range of 11–15 °C. However, our study is inconsistent with other studies that reported that heterotrophic nitrifiers in hot, semiarid grasslands can nitrify best at 40 °C, a value far above the optimal temperature for heterotrophic nitrifying activities (25 °C) in forested environments with a rainy and warm climate^41^. We must not ignore that the GHN rates in our study are often estimated under laboratory incubation conditions.

Global GNM is the key factor driving $I_{{\mathrm{NH}}_4}$ (Fig. 4a and Supplementary Fig. 4b), a relationship that is well established^6,8^, and this relationship is maintained across terrestrial ecosystems and climatic zones (Fig. 5b and Supplementary Fig. 9i). This is also confirmed by the highest rates of $I_{{\mathrm{NH}}_4}$ in tropical forests with higher GNM rates (Supplementary Fig. 5d). Soil GHN and total N were the main stimulators of global $I_{{\mathrm{NO}}_3}$ (Fig. 4a). The stimulating effect of GHN on $I_{{\mathrm{NO}}_3}$ is plausible, as both require high C availability, which is an unfavourable condition for GAN (Fig. 4a and Supplementary Table 4). GHN is thus a major source of NO_3_^−^ under these conditions, stimulating global $I_{{\mathrm{NO}}_3}$. Moreover, soils with a higher total N content often contain more microbial biomass^9^ and exhibit greater $I_{{\mathrm{NO}}_3}$ (ref. 8). Soil microbial biomass stimulates both GNM and GN globally^8,9^, which are positively correlated with $I_{{\mathrm{NO}}_3}$ (*P* < 0.001; Supplementary Fig. 10c,g), as they are responsible for providing a NO_3_^−^ substrate to soil microorganisms. Positive associations of GHN and total soil N with $I_{{\mathrm{NO}}_3}$ were observed in croplands and forests but not in grasslands (Fig. 6d,e). Moreover, GHN controlled $I_{{\mathrm{NO}}_3}$ only in the humid subtropical areas, but soil total N controlled the $I_{{\mathrm{NO}}_3}$ rate in all climatic zones except for the continental regions (Supplementary Fig. 9j). We also found that the DNRA rate is primarily driven by mean annual precipitation (MAP) (Figs. 4a and 6f), which is in line with previous studies^42^ and is shown by the higher DNRA rates in the tropical and subtropical regions in our global predictions (Fig. 2b). Soil oxygen depletion as a result of increased moisture content leads to low redox potential, and then NO_3_^−^ is used as an electron acceptor, facilitating the reduction of NO_3_^−^ to NH_4_^+^ (ref. 42). By testing this relationship across climatic zones, we observed this connection in the marine west coast and tropical wet regions only (Supplementary Fig. 9l), and this was consistent with our global predictions (Fig. 2b). However, we did not observe significant differences in DNRA rates in terrestrial ecosystems across different climatic zones (Supplementary Fig. 5c). In addition, the highest rates of $I_{{\mathrm{NO}}_3}$ and DNRA were reported from humid subtropical zones, which may be due to the high ratio of soil NO_3_^−^ to NH_4_^+^ in this region compared with other regions (Supplementary Fig. 11), as NO_3_^−^ is the substrate for both processes^2^. The high precipitation rate in humid subtropical regions can increase the availability of soil substrate (for example, total N and C) and thus stimulate microbial activity^2,9^. Furthermore, we found that higher net NH_4_^+^ production rates are observed with enhanced GNM and DNRA and are suppressed by increasing $I_{{\mathrm{NH}}_4}$. In contrast, net NO_3_^−^ production rates are stimulated by enhanced GHN, GAN, net NH_4_^+^ production and soil pH and are suppressed by increasing $I_{{\mathrm{NO}}_3}$ (Fig. 4a).

### The implications of this study

A more detailed understanding of the global N cycle in response to various controls is of great interest to a wide readership, as this ultimately determines important processes such as the ecosystem response to climate change (for example, progressive N limitation theory). It is critical to understand the variability of soil gross N cycling rates resulting from the global spatial heterogeneity of climatic and edaphic variables, which is important for estimating the potential risk of N loss. Our study aimed to predict the global spatial variations of soil gross N cycling rates and highlights promising areas for future ^15^N gross transformation studies. The type of data used in this study has also been used in previous meta-studies, the last one almost 20 years ago^8^. Although most gross N transformation rates in our synthesis are possibly not representative of in situ rates (owing to laboratory investigation and the lack of plants), this study provides an overview of the current state of knowledge on gross N rates that goes far beyond previous studies with a limited number of observations^8^. Unlike previous studies, we are able to draw firm conclusions. Our study shows that soil NO_3_^−^ retention is lower overall, with wide ratios of soil NO_3_^−^ to NH_4_^+^ and of soil GAN to $I_{{\mathrm{NH}}_4}$, indicating a leaky N cycle. The global patterns of the soil N cycle change from conservative in forests to leaky in croplands. We also found a difference in the global N cycle across climatic zones (Supplementary Fig. 11). This underlines the importance of forests in the global N cycle and the need for further insights on NO_3_^−^ retention in croplands, as well as the potential effect of climate change on the global soil N cycle, as discussed below.

#### Importance of forests in the global N cycle

Our study revealed that land use was the most important factor affecting the ratios of GAN to $I_{{\mathrm{NH}}_4}$ and of soil NO_3_^−^ to NH_4_^+^ (Fig. 4b,c). We did not observe significant differences in GAN rates across climatic zones, confirming that land use was more important in controlling GAN rates than climate^23^. Land use is thus likely to be the controlling factor of the potential risk of global soil N losses. The low ratios of GAN to $I_{{\mathrm{NH}}_4}$ and of soil NO_3_^−^ to NH_4_^+^ in forests imply that GNM and GI are tightly coupled (Fig. 1b,c), indicating that forests can effectively conserve reactive available N^4,23^. In contrast, nitrification was the main fate of NH_4_^+^ from GNM in croplands (Fig. 1d). Converting croplands to forests would improve soil N retention and minimize N losses to the environment, but this may be difficult to achieve given the need to maintain food security for a rapidly growing population. Instead, we suggest that expanding agroforestry can be a solution that can improve N conservation, among many other benefits. In agroforestry systems, the deep tree rooting can catch and recycle subsoil inorganic N leached below the rooting zone of linked croplands, causing a more efficient interception of the leaked N^43^. Moreover, NH_4_^+^ consumption in tree-based systems is higher, leaving less NH_4_^+^ N for nitrification and thus lowering soil N losses, compared with cropland systems^44^. A recent meta-analysis reported that soil organic C and N storage and available N increased by 21%, 13% and 46%, respectively, under agroforestry compared with crop monocultures^43^. Increased soil organic C content in agroforests compared with cropland makes the soil N cycle more conservative^43,44^ and is therefore an important factor for climate-smart agricultural systems.

#### Understanding NO_3_^−^ retention in croplands

Although we noticed that $I_{{\mathrm{NO}}_3}$ and DNRA occur, they are low and consume only 20% of the total NO_3_^−^ production in croplands, demonstrating a lower NO_3_^−^ retention in croplands than in other land use systems. DNRA and $I_{{\mathrm{NO}}_3}$ are favoured by increasing levels of soil C (Supplementary Tables 7 and 11), a condition that restricts GAN (Supplementary Table 4). A rapid depletion of NH_4_^+^ was observed when C (for example, in the form of crop residues) was added to soil, causing microorganisms to immobilize NO_3_^−^ to maintain their growth, which in turn promotes $I_{{\mathrm{NO}}_3}$ ideally with negligible denitrification N loss^45^. Concurrently, C supplies electrons through respiration or fermentation, which facilities the reduction of NO_3_^−^ to NH_4_^+^, providing energy to DNRA bacteria^46^. Exogenous organic C additions can thus promote $I_{{\mathrm{NO}}_3}$ and DNRA while restricting GAN (Fig. 4a), reducing soil NO_3_^−^ accumulation in croplands.

#### Climatic change may influence global N cycling

Our global analysis revealed that higher temperatures directly reduce GHN and indirectly reduce GNM, GAN, $I_{{\mathrm{NO}}_3}$ and $I_{{\mathrm{NH}}_4}$ via reducing soil total N (Fig. 4a). Soil microbial maintenance costs increase with higher temperatures, causing higher energy requirements and lower microbial C use efficiency, which results in lower microbial biomass and gross N transformation rates^9,47^. Global warming may thus reduce gross N transformation rates in the long run while stimulating it in the short term. Furthermore, the global hydrological cycle is intensifying and will continue to do so in the future, with a global redistribution of precipitation (wet sites become wetter, dry sites drier) and more intense rainfall accompanied by longer and more intense droughts. Soil microbial biomass and N_2_O emissions decrease with increasing drought intensity, while decreasing precipitation significantly increases extractable NH_4_^+^ (ref. 48). Sustained N processing during drought could thus lead to greater N losses during subsequent wetting events. This can also be seen in the increasing soil δ^15^N values, which are due to the effects of drier conditions^49^.

Finally, our SEM showed that 53%, 79%, 87%, 73%, 36% and 90% of the variation in GNM, $I_{{\mathrm{NH}}_4}$, $I_{{\mathrm{NO}}_3}$, GHN, GAN and DNRA, respectively, is still unexplained, which may be because the influence of microbial community structures was not included in the analysis. Although the regression analysis (Supplementary Tables 1, 3 and 4) revealed a vital role of soil microorganisms in controlling soil gross N cycling rates globally, our SEM did not include the effect of soil microorganisms due to the paucity of data. Climate change, N deposition and/or anthropogenic disturbances affect soil microbial community composition^50^, which might affect soil gross N cycling rates and, eventually, soil N availability and loss. Consequently, future studies should focus more on the effects of microbial community composition on soil gross N cycling rates, which will improve the prediction of soil N cycling under future global changes.

### Sources of uncertainty

Although our dataset is much larger than those used in previous syntheses, large uncertainties in our estimate of the global N cycle pattern in terrestrial ecosystems still exists. There are three sources of uncertainties.

#### Influence of controlled laboratory conditions

Most studies included in our dataset were based on laboratory experiments conducted under controlled conditions using disturbed soils and often without plants; these are not necessarily representative of in situ conditions^8,13^. For example, GNM rates were up to five times lower in the laboratory than in the field^14^. Soil disturbance such as sieving can alter soil bulk density, aggregate structure, soil aeration, nutrient availability, and the abundance and activity of microorganisms, with consequences for soil N transformations. This is seen, for instance, in an immediate increase in GNM and $I_{{\mathrm{NH}}_4}$ and suppression of $I_{{\mathrm{NO}}_3}$, but with no effect on GN^13^. Even if GNM is unaffected, a redistribution of substrate may enhance the contact with immobilizers^10^. In addition, the form in which N fertilizers are applied (for example, as granules in the field or as a liquid in the laboratory) affects the availability of N. Laboratory studies are often carried out with only soil. However, the interactions of plants via C rhizodeposits can affect the soil microbial community and consequently the associated N transformations^16^. For example, GNM and GHN were stimulated by the presence of wheat^15^, whereas soil microorganisms switched to assimilate NO_3_^−^ in the presence of NH_4_^+^-preferring plants^16^. Soil moisture and temperature conditions in the laboratory are carefully controlled, but these conditions are variable in the field. In particular, the fluctuating effect of soil moisture due to precipitation creates conditions referred to as hot moments, where N transformations are different compared with controlled conditions^51^, which is also reflected by the high temporal variability of N_2_O emissions^52^. Soil N_2_O emissions also vary significantly with fertilizer application mode^53^, crop type^54^, irrigation pattern^55^ and tillage practice^53^. Since these agricultural factors can influence N_2_O emissions in the field but not in the laboratory, laboratory studies should be interpreted with caution and, if possible, validated under field conditions.

#### Influence of substrate addition

Our dataset contained results from ^15^N pool dilution and tracing techniques. These techniques are the most commonly used methods for measuring gross N cycling rates; however, the ^15^N label addition can increase the size of soil N pools (that is, NH_4_^+^ and NO_3_^−^), which can stimulate the gross N consumption rates^56^. This seems to be less of a problem if only low amounts of ^15^N are applied^56,57^, which is also dependent on the ecosystem (that is, if it is used to only low N amounts)^14^. In temperate grassland soils, for example, larger amounts of highly enriched ^15^N have often been applied^58^, whereas in low-fertility arable soils, smaller N amounts have been used^59^. In forest soils, typically 5% of the initial pool size is applied^60^. Most N additions used in the ^15^N isotopic pool dilution technique in our dataset were small (0.001–5.0 mg N kg^−1^), but our synthesis also included studies that used large additions of N, which probably led to an overestimation of $I_{{\mathrm{NH}}_4}$ and $I_{{\mathrm{NO}}_3}$. The combination of the ^15^N isotopic pool dilution technique with an estimate of the net N turnover in separate samples that do not receive ^15^N additions (that is, the reformed difference approach) can be an improvement^8,57^. Furthermore, ^15^N tracing studies running for long enough that mineral N pools return to background values can evaluate the stimulation effect of N additions^61^. Advanced natural ^15^N abundance techniques can also be useful for studying N dynamics without any N application.

#### Machine-learning-based global maps

Machine learning typically relies on the variance of predictions made by ensembles of models^62^, such as the random forests (RF) algorithm. Each tree in RF is a model of an ensemble, and the variation in predictions between individual trees is utilized to estimate uncertainties. One issue with these approaches is that information for unknown environments is unavailable, because they do not take into account dissimilarities in the predictor space between new and training data^63^. The recent suggestion by Meyer and Pebesma^63^ to add the area of applicability to the modeller’s standard toolkit and to report a map of dissimilarity-index-dependent performance estimates alongside prediction maps may provide improved uncertainty estimates. The area of applicability was not estimated in our study, but our standard deviation maps clearly reveal areas where the models perform poorly or extrapolate with higher standard deviations than the root mean square error (RMSE) (for example, deserts, polar regions and other regions where no observations were available) (Figs. 2 and 3). Our analysis thus highlights promising areas for future ^15^N gross transformation studies—that is, areas characterized by high uncertainties. Generally, the close correlation between modelled and observed gross N transformation (close to the 1:1 line) confirmed the usefulness of the ensemble machine learning (Supplementary Fig. 12).

## Methods

### Data compilation and overview of the dataset

All peer-reviewed publications published before December 2020 that examined soil gross N transformation rates were systematically collected by searching Google Scholar and the Web of Science database. We also searched within these publications for references. Studies that have been included in previous meta-analyses of gross N transformation rates were also included in our synthesis^8^. We used the following search terms: ‘gross nitrogen rates’, ‘soil gross nitrogen transformation’, ‘gross nitrogen mineralization’, ‘gross nitrification’, ‘gross nitrogen immobilization’ and ‘gross dissimilatory nitrate reduction to ammonium’. We followed PRISMA guidelines to conduct the literature search (Supplementary Fig. 13). We employed the following criteria for compiling gross N transformation rate data: (1) soil gross N transformation rates were quantified using the topsoil samples (0–20 cm), (2) most of the incubation periods for gross N transformation rates ranged from 24 to 48 h and (3) the ^15^N isotopic pool dilution technique and/or tracing models were used to measure gross N transformation rates. In total, 398 studies met these criteria (Supplementary Fig. 13 and Supplementary References). The dataset of gross N transformation rates was created by compiling 4,032 observations representing data from isotope tracing assays in different ecosystems. We evaluated a total of 1,065, 434, 413, 437, 240, 171, 903, 233 and 136 observations for GNM, $I_{{\mathrm{NH}}_4}$, $I_{{\mathrm{NO}}_3}$, GI, GAN, GHN, GN, DNRA and N_2_O emission, respectively. The global distribution of study sites for gross N transformation rates included in our study is shown in Supplementary Fig. 1a,b. In large-scale pattern analysis, measurements from organic, mineral and mixed (organic + mineral) soil horizons or from disturbed and intact soils were included; however, we only used data from disturbed mineral soil horizons to compare ecosystem types. Most of the collected studies (312 studies) were conducted under controlled laboratory conditions.

Two authors performed data extraction independently, aiming to extract from the eligible studies the detailed site information such as climatic zone, latitude, longitude, ecosystem type, MAT, MAP and soil chemical (pH, total C and N, C/N ratio, and extractable NH_4_^+^-N and NO_3_^−^-N) and biological (microbial biomass C and N, fungi-to-bacteria ratio and the abundances of bacteria, ammonia-oxidizing bacteria, ammonia-oxidizing archaea and fungi) attributes, along with soil gross N transformation rates (GNM, GAN, GHN, GN, $I_{{\mathrm{NO}}_3}$, $I_{{\mathrm{NH}}_4}$, GI and DNRA). The data on the emission of N_2_O were also collected from the original articles. The ratios of GAN to $I_{{\mathrm{NH}}_4}$, GAN to GNM and NO_3_^−^ to NH_4_^+^ were computed and included in the analysis. We also calculated the net NH_4_^+^ and NO_3_^−^ production rates. GetData (v.2.22) (http://getdata-graph-digitizer.com) was used to extract the data contained in graphs. All geographical regions except Antarctica are represented in our dataset, with a wide range of MAP (266–7,000 mm yr^−1^) and MAT (−4.80 to 28.5 °C). Terrestrial ecosystems in our dataset included forests (58%), grasslands (15%) and croplands (25%). We coded climatic zones as marine west coast, the Mediterranean, tropical wet, continental and humid subtropical according to the Köppen classification system.

### Data analyses

We checked the normality of the data using the Kolmogorov–Smirnov test. If the data did not show a normal distribution, a transformation to the natural logarithm was performed to approximate normality and stabilize the distribution.

#### Global patterns of the N cycle

We calculated the average (±standard error) gross N cycling rates globally and across soil layers and different types of ecosystems. Since there were insufficient data for GAN and GHN in forest organic layers, we used GN to compare mineral and organic soil layers in forests. Differences in gross N cycling rates among soil layers and ecosystem types were tested using analysis of variance with least significant differences for multiple comparisons. Moreover, regression analysis was used to analyse the relationships between soil and climatic variables and gross N transformation rates and between gross N transformation rates and each other (Supplementary Tables 1–10).

#### SEM and mixed-effects meta-regression analysis

The variance inflation factor was used to estimate the collinearities among variables, and variables with a variance inflation factor value of >5 were excluded. We then conducted an SEM using the lavaan package^64^ in R to test how gross N transformation rates (GNM, GHN, GAN, $I_{{\mathrm{NH}}_4}$, $I_{{\mathrm{NO}}_3}$ and DNRA) and net NH_4_^+^ and NO_3_^−^ production rates are impacted by soil variables (for example, pH, total N and C/N ratio) and climatic variables (MAT and MAP) and by each other. The conceptual SEM included the direct impacts of soil properties and climatic variables on gross and net N transformation rates as well as the effects of gross N transformation rates on each other and on net N production rates. It also included the indirect effects of climatic variables on gross N transformation rates via changing soil attributes. To evaluate the conceptual models, we used goodness-of-fit statistics (comparative fit index, 0.94; Tucker–Lewis index, 0.90). Furthermore, we tested the effect of soil variables (for example, pH, total N and C/N ratio) and climatic variables (MAT and MAP) and/or land use on the ratios of GAN to $I_{{\mathrm{NH}}_4}$ and of soil NO_3_^−^ to NH_4_^+^ in a mixed-effects meta-regression model using the glmulti package^65^ in R. We estimated the importance of each variable as the sum of Akaike weights for models that included this variable, which is considered as the overall support for each variable across all models. To explore the most important variables, we set the cut-off to 0.8.

### Global prediction

#### Model development for global prediction

The network of gross N transformation rates was predicted using five machine learning models: RF, support vector machine (svmRadial), generalized boosted regression models (gbm), stepwise regression (leapSeq) and generalized linear models (glmnet). Three of these machine learning methods (RF, gbm and svmRadial) are based on decision trees and boosting approaches, while two are linear regression models (leapSeq and glmnet). The caret^66^ and caretEnsemble^67^ packages were used to combine the five approaches, and the single best prediction model was built from these five base models. We created gross N transformation rate models using environmental variables, including climatic factors, soil attributes (pH, N, C, bulk density and clay content) and land use cover (Supplementary Fig. 3a–h). Recursive feature elimination was utilized to estimate the number of covariates that should be included in the model fit. Once a predetermined number of covariates had been reached, the least significant explanatory variable was gradually eliminated to reduce computational load and ensure that the average resolution of all covariates was equal with a spatial resolution of 1 km. These variables were collected from a worldwide collection of soil and climatic property information. The soil property database, which has a geographical resolution of 1.0 km, was derived from the International Soil Reference and Information Centre’s World Inventory of Soil Emissions database (www.isric.org)^68^. Climatic data with a resolution of 0.5° was obtained using the getdata function from the raster package^69^. The land use system was sourced from the Food and Agriculture Organization of the United Nations^70^. The freely available world base map data were downloaded from the Global Administrative Areas Database (https://gadm.org/index.html). The worldwide distribution maps were created using the ESRI ArcGIS program^71^. All gross N transformation rate predictions in this investigation were made using R v.4.1.1, and the R scripts that were used are available on Figshare^72^.

#### Model validation

We assessed the prediction of gross N transformation rates using tenfold cross-validation with five repeats. The whole database was subsampled into ten subsamples, nine of which served as training data and one as test data. We averaged the test results from each subsample to estimate the model’s performance. The RMSE, the regression coefficients of determination (*R*^2^) and the mean of the absolute value of errors (MAE) are three extensively used validation indicators that were calculated according to the following formulas:

$${\mathrm{MAE}} = \frac{1}{n}\mathop {\sum }\nolimits_{i = 1}^n \left| {P_i - O_i} \right|$$

$${\mathrm{RMSE}} = \sqrt {\frac{1}{n}\mathop {\sum }\limits_{i = 1}^n \left( {P_i - O_i} \right)^2}$$

$$R^2 = \frac{{\mathop {\sum }\nolimits_{i = 1}^n \left( {P_i - \bar O_i} \right)^2}}{{\mathop {\sum }\nolimits_{i = 1}^n \left( {O_i - \bar O_i} \right)^2}}$$

where *n* is the number of samples, and *P*_*i*_*, O*_*i*_ and $\bar O$ are the predicted, observed and mean of observed values, respectively.

The model with the lowest MAE, lowest RMSE and greatest *R*^2^ for each gross N transformation rate was selected as the best (Supplementary Fig. 2a,b). We used the selected model to map the global gross N transformation rates. The *R*^2^ values of the best models are shown in Supplementary Fig. 12. To evaluate the uncertainty of the produced maps, we characterized the distributions of mean, median and quantile values (upper and lower). These four values were computed for each N transformation predicted, and the standard deviation of each map was then derived from these values (mean, median, and upper and lower boundaries). The standard deviation map was used for evaluating the uncertainties of the produced maps. These quantiles were used to express the uncertainties of the global soil map^73^.

### Reporting summary

Further information on research design is available in the Nature Portfolio Reporting Summary linked to this article.

## Supplementary information

Supplementary Information Supplementary Tables 1–10, Figs. 1–13 and References.

Reporting Summary

Supplementary Data PRISMA checklist.

Supplementary Data 1 All the data included in our study.

Supplementary Data 2 A subset of data for sites that measured the full N cycling rates or most variables of soil N processes.

## Data Availability

The data supporting the findings of this study are available in Supplementary Data 1 and 2. The data underlying Figs. 2 and 3 are available on Figshare (10.6084/m9.figshare.21406731.v4). Source data are provided with this paper.

## Source data

Source Data Fig. 1 Statistical source data.
Source Data Fig. 4 Statistical source data.
Source Data Fig. 5 Statistical source data.
Source Data Fig. 6 Statistical source data.

## Code availability

The R (v.4.1.2) code used to generate the results and figures reported in this study is available on Figshare (10.6084/m9.figshare.21406731.v4).
