# Supplementary material for: Expanding agroforestry can increase nitrate retention and mitigate the global impact of a leaky nitrogen cycle in croplands
Source: Nat Food. 2022 Dec 28;4(1):109–21. doi: 10.1038/s43016-022-00657-x (PMC10154242; doi:10.1038/s43016-022-00657-x)
Supplement: Supplementary file 1 — Supplementary Tables 1–10, Figs. 1–13 and References. [file 43016_2022_657_MOESM1_ESM.pdf]

nature food

Analysis

https://doi.org/10.1038/s43016-022-00657-x

Expanding agroforestry can increase nitrate retention and mitigate the global impact of a leaky nitrogen cycle in croplands

In the format provided by the authors and unedited

Table of contents

Table S1. Simple linear regression equations of relationships between gross N mineralization and environmental factors.

Table S2. Simple linear regression equations to describe relationships between gross nitrification and environmental factors.

Table S3. Simple linear regression equations to describe relationships between gross heterotrophic nitrification and environmental factors.

Table S4. Simple linear regression equations to describe relationships between gross autotrophic nitrification and environmental factors.

Table S5. Simple linear regression equations for relationships between gross N immobilization rate and environmental factors.

Table S6. Simple linear regression equations for relationships between gross ammonium immobilization rate and environmental factors.

Table S7. Simple linear regression equations for relationships between gross nitrate immobilization rate and environmental factors.

Table S8. Simple linear regression equations for relationships between gross autotrophic nitrification to ammonium immobilization and environmental factors.

Table S9. Simple linear regression equations for relationships between extractable nitrate to extractable ammonium ratio and environmental factors.

Table S10. Simple linear regression equations for relationships between soil dissimilatory nitrate reduction to ammonium and environmental factors.

Fig. S1. Global distribution of study sites included in our study.

Fig. S2. Results of 10-fold cross-validation of five predictive models for gross N transformation rates.

Fig. S3. The spatial pattern of soil and climatic variables at the global scale.

Fig. S4. Relationship of gross autotrophic nitrification to ammonium immobilization ratio with nitrous oxide emissions at the global scale.

Fig. S5. Changes (means ± SE) of soil gross N mineralization, gross nitrification, ammonium immobilization, and nitrate immobilization with terrestrial ecosystems in different climatic zones.

Fig. S6. Changes (means ± SE) of soil dissimilatory nitrate reduction to ammonium, NO_3^-/NH_4^+ ratio, and soil gross nitrification to ammonium immobilization ratio with terrestrial ecosystems in different climatic zones.

Fig. S7. Changes (means ± SE) of gross N transformation rates in mineral and organic layers of forest soils at the global scale.

Fig. S8. Changes (means ± SE) of gross N cycling in the mineral soil of forests and croplands (subset of data for sites that measured the full N cycling rates or most variables of soil N processes).

Fig. S9. Relationships of gross N transformation rates to each other and to environmental factors across climatic zones.

Fig. S10. Relationships of gross N transformation rates to each other at the global scale.

Fig. S11. Changes (mean± SE) of gross N cycling rates in different climatic zones.

Fig. S12. Relationship between measured and predicted gross N transformation rates at the global scale.

Fig. S13. A RISMA diagram showing the process involved for selection of publications included in this study.

Appendix. List of references containing data used in the analyses.

Table S1. Simple linear regression equations of relationships between gross N mineralization (GNM, mg N kg^{-1} day^{-1}) and environmental factors (all analyses were based on transformed data except for soil pH).

**Variable/process**	**Equation**	R^2	***p***	**N**
Mean annual precipitation (MAP, mm)	Ln GNM = 0.337 × Ln MAP – 1.101	0.11	< 0.0001	823
Mean annual temperature (MAT, ºC)	Ln GNM = 0.272 × Ln MAT + 2.017	<0.01	0.212	822
Soil pH	Ln GNM = – 0.263 × pH + 2.654	0.11	< 0.0001	807
Total carbon (C, g kg^{-1})	Ln GNM = 0.563 × Ln C – 0.838	0.31	< 0.0001	804
Total nitrogen (N, g kg^{-1})	Ln GNM = 0.640 × Ln N + 0.592	0.32	< 0.0001	791
Soil C:N	Ln GNM = 0.554 × Ln C:N + 0.240	0.03	< 0.0001	766
Microbial biomass C (MBC, mg kg^{-1})	Ln GNM = 0.797 × Ln MBC – 3.761	0.51	< 0.0001	280
Microbial biomass N (MBN, mg kg^{-1})	Ln GNM = 0.734 × Ln MBN – 1.776	0.45	< 0.0001	317
Abundance of fungi (F, gene copies g^{-1})	Ln GNM = 0.426 × Ln F –7.243	0.36	< 0.0001	32
Abundance of bacteria (B, gene copies g^{-1})	Ln GNM = 0.353 × Ln B –7.240	0.26	0.002	34
F:B	Ln GNM = – 0.345 × Ln F:B –1.428	0.13	0.007	30
Microbial respiration (R, mg kg^{-1} day^{-1})	Ln GNM = 0.831 × Ln R – 1.366	0.69	< 0.0001	119

Statistical significance (*p* value) was obtained by two-tailed Student's t test. N is the number of observations.

Table S2. Simple linear regression equations to describe relationships between gross nitrification (GN, mg N kg^{-1} day^{-1}) and environmental factors (all analyses were based on transformed data except for soil pH).

**Variable/process**	**Equation**	R^2	***p***	**N**
Mean annual precipitation (MAP, mm)	Ln GN = – 0.089 × Ln MAP + 0.916	<0.01	0.288	800
Mean annual temperature (MAT, ºC)	Ln GN = 0.006 × Ln MAT +3.042	<0.01	0.432	785
Soil pH	Ln GN = 0.317 × pH – 1.319	0.08	<0.0001	751
Total carbon (C, g kg^{-1})	Ln GN = 0.155 × Ln C – 0.231	0.02	0.001	758
Total nitrogen (N, g kg^{-1})	Ln GN = 0.475 × Ln N – 0.090	0.14	<0.0001	747
Soil C:N	Ln GN = –1.864 × Ln C: N + 5.302	0.20	<0.0001	647
Microbial biomass C (MBC, mg kg^{-1})	Ln GN = 0.413 × Ln MBC – 2.299	0.14	<0.0001	218
Microbial biomass N (MBN, mg kg^{-1})	Ln GN = 0.500 × Ln MBN – 1.752	0.17	<0.0001	242
MBC: MBN	Ln GN = – 0.919 × Ln MBC: MBN + 2.299	0.10	<0.0001	179
Microbial respiration (R, mg kg^{-1} day^{-1})	Ln GN = 0.268 × Ln R – 0.198	0.11	0.004	77
Ammonia-oxidizing archaea (AOA; copies g^{-1} soil)	Ln GN = – 0.018 × Ln AOA + 0.834	0.00	0.745	70
Ammonia-oxidizing bacteria (AOB; copies g^{-1} soil)	Ln GN = 0.117 × Ln AOB – 1.051	0.09	0.011	72
Abundance of fungi (F, copies g^{-1} soil)	Ln GN = – 0.176 × Ln F + 3.991	0.11	0.025	45
Abundance of bacteria (B, copies g^{-1} soil)	Ln GN = 0.0005 × Ln B + 0.497	<0.01	0.994	49
F: B	Ln GN = – 0.154 × Ln F:B – 0.488	0.04	0.148	49
Extractable NH_4^+ (mg N kg^{-1})	Ln GN = – 0.027 × Ln NH_4 – 0.433	<0.01	0.562	456
Gross N mineralization (GNM, mg N kg^{-1} day^{-1})	Ln GN = 0.554 × Ln GNM – 0.412	0.20	<0.0001	813

Statistical significance (*p* value) was obtained by two-tailed Student’s t test. N is the number of observations.

Table S3. Simple linear regression equations to describe relationships between gross heterotrophic nitrification (GHN, mg N kg^{-1} day^{-1}) and environmental factors (all analyses were based on transformed data except for soil pH).

**Variable/process**	**Equation**	R^2	***p***	**N**
Mean annual precipitation (MAP, mm)	Ln GHN = – 0.174 × Ln MAP – 0.238	<0.01	0.649	154
Mean annual temperature (MAT, ºC)	Ln GHN = – 2.0 × Ln MAT + 6.712	0.18	< 0.0001	144
Soil pH	Ln GHN = – 0.312 × pH + 0.242	0.02	0.070	153
Total carbon (C, g kg^{-1})	Ln GHN = 0.796 × Ln C – 3.405	0.15	<0.0001	132
Total nitrogen (N, g kg^{-1})	Ln GHN = 0.930× Ln N – 1.409	0.22	<0.0001	134
Soil C: N	Ln GHN = – 0.582 × Ln C: N + 0.154	0.01	0.229	140
Ammonia-oxidizing archaea (AOA; copies g^{-1} soil)	Ln GHN = – 0.354 × Ln AOA + 3.417	0.07	0.123	34
Ammonia-oxidizing bacteria (AOB; copies g^{-1} soil)	Ln GHN = – 0.117 × Ln AOB – 0.219	0.02	0.412	32
Abundance of fungi (F, copies g^{-1} soil)	Ln GHN = 0.645 × Ln F – 14.16	0.55	<0.0001	18
Abundance of bacteria (B, copies g^{-1} soil)	Ln GHN = 0.115 × Ln B – 4.561	0.02	0.570	22
F: B	Ln GHN = 0.248 × Ln F: B + 0.115	0.54	0.002	15
Gross nitrification (GN, mg N kg^{-1} day^{-1})	Ln GHN = 0.673 × Ln GN – 1.444	0.32	<0.0001	156
Gross N mineralization (GNM, mg N kg^{-1} day^{-1})	Ln GHN = 0.513 × Ln GNM – 2.146	0.05	0.008	130
Extractable NH_4^+ (mg N kg^{-1})	Ln GHN = 0.213 × Ln NH_4 – 1.709	0.02	0.159	107

Statistical significance (*p* value) was obtained by two-tailed Student's t test. N is the number of observations.

Table S4. Simple linear regression equations to describe relationships between gross autotrophic nitrification (GAN, mg N kg^{-1} day^{-1}) and environmental factors (all analyses were based on transformed data except for soil pH).

**Variable/process**	**Equation**	R^2	***p***	**N**
Mean annual precipitation (MAP, mm)	Ln GAN = – 1.235 × Ln MAP + 8.394	0.11	<0.0001	217
Mean annual temperature (MAT, ºC)	Ln GAN = – 0.652 × Ln MAT + 1.767	0.01	0.133	214
Soil pH	Ln GAN = 0.977 × pH – 5.454	0.38	<0.0001	214
Total carbon (C, g kg^{-1})	Ln GAN = – 0.251 × Ln C + 0.592	0.01	0.109	211
Total nitrogen (N, g kg^{-1})	Ln GAN = 0.766× Ln N – 0.668	0.09	<0.0001	202
Soil C:N	Ln GAN = – 2.880 × Ln C:N + 7.092	0.31	<0.0001	204
Ammonia-oxidizing archaea (AOA; copies g^{-1} soil)	Ln GAN = 0.045 × Ln AOA – 0.943	0.01	0.668	35
Ammonia-oxidizing bacteria (AOB; copies g^{-1} soil)	Ln GAN = 0.338 × Ln AOB – 4.893	0.31	0.001	32
Abundance of fungi (F, copies g^{-1} soil)	Ln GAN = – 0.620 × Ln F + 11.96	0.55	<0.0001	20
Abundance of bacteria (B, copies g^{-1} soil)	Ln GAN = 0.624 × Ln B – 15.30	0.52	0.001	19
F:B	Ln GAN = – 0.762 × Ln F:B – 3.955	0.82	<0.0001	14
Gross nitrification (GN, mg N $kg^{-1}day^{-1}$)	Ln GAN = 1.123 × Ln GN – 0.680	0.80	<0.0001	214
Gross N mineralization (GNM, mg N kg^{-1} day^{-1})	Ln GAN = 0.513 × Ln GNM – 2.146	0.05	0.008	130
Extractable NH_4^+ (mg N kg^{-1})	Ln GAN = – 0.505 × Ln NH4 + 0.527	0.15	<0.0001	158

Statistical significance (*p* value) was obtained by two-tailed Student’s t test. N is the number of observations.

Table S5. Simple linear regression equations for relationships between gross N immobilization rate (GI, mg N kg^{-1} day^{-1}) and environmental factors (all analyses were based on transformed data except for soil pH).

**Variable/process**	**Equation**	R^2	***p***	**N**
Mean annual precipitation (MAP, mm)	Ln GI = – 0.031 × Ln MAP + 1.380	<0.01	0.722	394
Mean annual temperature (MAT, ºC)	Ln GI = – 0.361 × Ln MAT + 1.831	0.04	<0.0001	333
Soil pH	Ln GI = – 0.297 × pH + 2.750	0.07	<0.0001	333
Total carbon (C, g kg^{-1})	Ln GI = 0.651 × Ln C – 1.363	0.32	<0.0001	345
Total nitrogen (N, g kg^{-1})	Ln GI = 0.812 × Ln N + 0.287	0.39	<0.0001	314
Soil C:N	Ln GI = 0.538 × Ln C: N – 0.278	0.02	0.007	314
Microbial biomass C (MBC, mg kg^{-1})	Ln GI = 0.830 × Ln MBC – 4.059	0.51	<0.0001	125
Microbial biomass N (MBN, mg kg^{-1})	Ln GI = 0.909 × Ln MBN – 2.802	0.53	<0.0001	140
MBC:MBN	Ln GI = 0.666 × Ln MBC:MBN + 0.212	0.03	0.072	100
Microbial respiration (R, mg kg^{-1} day^{-1})	Ln GI = 0.531 × Ln R – 0.673	0.38	<0.0001	71
Extractable NH_4^+ (mg N kg^{-1})	Ln GI = 0.547 × Ln NH_4 + 0.131	0.30	<0.0001	185
Extractable NO_3^- (mg N kg^{-1})	Ln GI = 0.013 × Ln NO_3 + 1.125	<0.01	0.824	185
Gross N mineralization (GNM, mg N kg^{-1} day^{-1})	Ln GI = 0.965 × Ln GNM – 0.088	0.84	<0.0001	421
Gross nitrification (GN, mg N kg^{-1} day^{-1})	Ln GI = 0.353 × Ln GN + 0.992	0.16	<0.0001	328
Gross autotrophic nitrification (GAN, mg N kg^{-1} day^{-1})	Ln GI = 0.167 × Ln GAN + 0.857	0.05	0.234	29
Gross heterotrophic nitrification (GHN, mg N kg^{-1} day^{-1})	Ln GI = 0.220 × Ln GHN + 1.067	0.24	0.009	28

Statistical significance (*p* value) was obtained by two-tailed Student's t test. N is the number of observations.

Table S6. Simple linear regression equations for relationships between gross ammonium immobilization rate (I_{NH4}, mg N kg^{-1} day^{-1}) and environmental factors (all analyses were based on transformed data except for soil pH).

**Variable/process**	**Equation**	**R^2**	***p***	**N**
Mean annual precipitation (MAP, mm)	Ln I_{NH4}= 0.593 × Ln MAP – 3.422	0.04	<0.0001	390
Mean annual temperature (MAT, ºC)	Ln I_{NH4}= 0.069 × Ln MAT + 0.517	<0.01	0.778	392
Soil pH	Ln I_{NH4}= – 0.145 × pH + 1.500	0.01	0.052	382
Total carbon (C, g kg^{-1})	Ln I_{NH4}= 0.702 × Ln C – 1.572	0.23	<0.0001	356
Total nitrogen (N, g kg^{-1})	Ln I_{NH4}= 0.864 × Ln N – 0.141	0.29	<0.0001	358
Soil C:N	Ln I_{NH4}= 0.434 × Ln C:N – 0.420	0.01	0.080	375
Microbial biomass C (MBC, mg kg^{-1})	Ln I_{NH4}= 1.007 × Ln MBC – 5.007	0.68	<0.0001	97
Microbial biomass N (MBN, mg kg^{-1})	Ln I_{NH4}= 1.000 × Ln MBN – 2.833	0.74	<0.0001	111
MBC:MBN	Ln I_{NH4}= – 0.777 × Ln MBC:MBN + 3.243	0.05	0.036	91
Microbial respiration (R, mg kg^{-1} day^{-1})	Ln I_{NH4}= 0.880 × Ln R – 1.744	0.66	<0.0001	23
Extractable NH_4^+ (mg N kg^{-1})	Ln I_{NH4}= 0.449 × Ln NH_4 + 0.023	0.14	<0.0001	253
Extractable NO_3^- (mg N kg^{-1})	Ln I_{NH4}= –0.040 × Ln NO_3 + 0.804	<0.01	0.469	257
Gross N mineralization (GNM, mg N kg^{-1} day^{-1})	Ln I_{NH4}= 1.017 × Ln GNM – 0.412	0.61	<0.0001	410
Gross nitrification (GN, mg N kg^{-1} day^{-1})	Ln I_{NH4}= 0.261 × Ln GN + 0.599	0.05	<0.0001	406
Gross N immobilization (GI, mg N kg^{-1} day^{-1})	Ln I_{NH4}= 0.965 × Ln GI – 0.083	0.86	<0.0001	123
Gross autotrophic nitrification (GAN, mg N kg^{-1} day^{-1})	Ln I_{NH4}= – 0.048 × Ln GAN – 0.050	<0.01	0.540	166
Gross heterotrophic nitrification (GHN, mg N kg^{-1} day^{-1})	Ln I_{NH4}= 0.183 × Ln GHN + 0.382	0.06	0.007	115

Statistical significance (*p* value) was obtained by two-tailed Student's t test. N is the number of observations.

Table S7. Simple linear regression equations for relationships between gross nitrate immobilization rate (I_{NO3}, mg N kg^{-1} day^{-1}) and environmental factors (all analyses were based on transformed data except for soil pH).

**Variable/process**	**Equation**	**R^2**	***p***	**N**
Mean annual precipitation (MAP, mm)	Ln I_{NO3}= 0.171 × Ln MAP – 1.996	<0.01	0.297	354
Mean annual temperature (MAT, ºC)	Ln I_{NO3}= – 0.159 × Ln MAT – 0.407	<0.01	0.344	347
Soil pH	Ln I_{NO3}= – 0.083 × pH – 0.477	<0.01	0.276	342
Total carbon (C, g kg^{-1})	Ln I_{NO3}= 0.771 × Ln C – 3.122	0.18	<0.0001	324
Total nitrogen (N, g kg^{-1})	Ln I_{NO3}= 0.813 × Ln N – 1.469	0.18	<0.0001	322
Soil C:N	Ln I_{NO3}= 0.277 × Ln C:N – 1.672	<0.01	0.294	328
Microbial biomass C (MBC, mg kg^{-1})	Ln I_{NO3}= 0.745 × Ln MBC – 4.860	0.37	<0.0001	89
Microbial biomass N (MBN, mg kg^{-1})	Ln I_{NO3}= 0.650 × Ln MBN – 2.842	0.37	<0.0001	100
MBC:MBN	Ln I_{NO3}= – 1.706 × Ln MBC:MBN + 3.468	0.24	<0.0001	83
Microbial respiration (R, mg kg^{-1} day^{-1})	Ln I_{NO3}= 0.049 × Ln R – 0.066	0.42	0.016	13
Extractable NH_4^+ (mg N kg^{-1})	Ln I_{NO3}= 0.262 × Ln NH_4 – 1.161	0.05	<0.0001	247
Extractable $NO_3^–$ (mg N $kg^{–1}$)	Ln I_{NO3}= – 0.088 × Ln NO_3 – 0.499	0.01	0.132	250
Gross N mineralization (GNM, mg N $kg^{–1}$ $day^{–1}$)	Ln I_{NO3}= 0.801 × Ln GNM – 1.711	0.25	<0.0001	362
Gross nitrification (GN, mg N $kg^{–1}$ $day^{–1}$)	Ln I_{NO3}= 0.478 × Ln GN – 0.816	0.17	<0.0001	361
Gross N immobilization (GI, mg N kg^{-1} day^{-1})	Ln I_{NO3}= 0.761 × Ln GI – 1.278	0.36	<0.0001	131
Gross autotrophic nitrification (GAN, mg N $kg^{–1}$ $day^{–1}$)	Ln I_{NO3}= – 0.213 × Ln GAN – 1.700	0.04	0.014	160
Gross heterotrophic nitrification (GHN, mg N $kg^{–1}$ $day^{–1}$)	Ln I_{NO3}= 0.378 × Ln GHN – 0.682	0.24	<0.0001	106

Statistical significance (*p* value) was obtained by two-tailed Student’s t test. N is the number of observations.

Table S8. Simple linear regression equations for relationships between gross autotrophic nitrification (GAN) to ammonium immobilization (I_{NH4}) and environmental factors (all analyses were based on transformed data except for soil pH).

**Variable/process**	**Equation**	R^2	***p***	**N**
Mean annual precipitation (MAP, mm)	Ln GAN: I_{NH4} = – 1.709 × Ln MAP + 11.435	0.11	<0.0001	157
Mean annual temperature (MAT, ºC)	Ln GAN: I_{NH4} = – 0.209 × Ln MAT – 0.071	<0.001	0.477	152
Soil pH	Ln GAN: I_{NH4} = 0.962 × pH – 5.662	0.18	<0.0001	158
Total carbon (C, g kg^{-1})	Ln GAN: I_{NH4} = – 0.411 × Ln C + 0. 855	0.02	0.090	156
Total nitrogen (N, g kg^{-1})	Ln GAN: I_{NH4}= 0.462 × Ln N – 0.774	0.05	0.046	157
Soil C:N	Ln GAN: I_{NH4} = –2.741 × Ln C:N + 6.610	0.17	<0.0001	155
Gross N mineralization (GNM, mg N kg^{-1} day^{-1})	Ln GAN: I_{NH4} = – 0.694 × Ln GNM + 0.193	0.06	0.001	170

Statistical significance (*p* value) was obtained by two-tailed Student’s t test. N is the number of observations.

Table S9. Simple linear regression equations for relationships between extractable nitrate (NO_3^-) to extractable ammonium (NH_4^+) ratio and environmental factors (all analyses were based on transformed data except for soil pH).

**Variable/process**	**Equation**	**R^2**	***p***	**N**
Mean annual precipitation (MAP, mm)	Ln NO_3^-: NH_4^+ = – 0.227 × Ln MAP + 1.494	0.01	0.09	496
Mean annual temperature (MAT, ºC)	Ln NO_3^-: NH_4^+ = – 0.076 × Ln MAT + 0.153	<0.01	0.56	467
Soil pH	Ln NO_3^-: NH_4^+ = 0.515 × pH – 2.669	0.14	<0.0001	473
Total carbon (C, g kg^{-1})	Ln NO_3^-: NH_4^+ = – 0.645 × Ln C + 2. 360	0.15	<0.0001	465
Total nitrogen (N, g kg^{-1})	Ln NO_3^-: NH_4^+ = – 0.519 × Ln N – 0.585	0.07	<0.0001	476
Soil C:N	Ln NO_3^-: NH_4^+ = –2.135 × Ln C:N + 5.666	0.18	<0.0001	459
Microbial biomass C (MBC, mg kg^{-1})	Ln NO_3^-: NH_4^+ = – 0.660 × Ln MBC + 4.122	0.22	<0.0001	121
Microbial biomass N (MBN, mg kg^{-1})	Ln NO_3^-: NH_4^+ = – 0.580 × Ln MBN + 2.320	0.17	<0.0001	164
MBC:MBN	Ln NO_3^-: NH_4^+ = – 0.915 × Ln MBC:MBN + 1.476	0.05	0.020	106
Ammonia-oxidizing archaea (AOA; copies g^{-1} soil)	Ln NO_3^-: NH_4^+ = 0.410 × Ln AOA – 6.313	0.24	0.002	39
Ammonia-oxidizing bacteria (AOB; copies g^{-1} soil)	Ln NO_3^-: NH_4^+ = 0.294 × Ln AOB – 3.383	0.26	0.002	34
Abundance of fungi (F, copies g^{-1} soil)	Ln NO_3^-: NH_4^+ = 0.272 × Ln F – 6.195	0.19	0.016	30
Abundance of bacteria (B, copies g^{-1} soil)	Ln NO_3^-: NH_4^+ = – 0.282 × Ln B – 5.748	0.17	0.030	28
F:B	Ln NO_3^-: NH_4^+ = – 0.039 × Ln F:B + 0.069	<0.01	0.613	31
Gross N mineralization (GNM, mg N kg^{-1} day^{-1})	Ln NO_3^-: NH_4^+ = – 0.421 × Ln GNM + 0.454	0.07	<0.0001	471
Gross nitrification (GN, mg N kg^{-1} day^{-1})	Ln NO_3^-: NH_4^+ = 0.511 × Ln GN – 0.136	0.15	<0.0001	449
Gross autotrophic nitrification (GAN, mg N kg^{-1} day^{-1})	Ln NO_3^-: NH_4^+ = 0.623 × Ln GAN + 0.678	0.42	<0.0001	160
Gross heterotrophic nitrification (GHN, mg N kg^{-1} day^{-1})	Ln NO_3^-: NH_4^+ = – 0.015 × Ln GHN + 0.406	<0.01	0.849	105
Gross N immobilization (GI, mg N kg^{-1} day^{-1})	Ln NO_3^-: NH_4^+ = – 0.462 × Ln GI + 0.125	0.12	<0.0001	172
Gross NH_4^+ immobilization (I_{NH4}, mg N kg^{-1} day^{-1})	Ln NO_3^-: NH_4^+ = – 0.324 × Ln I_{NH4} + 0.187	0.09	<0.0001	247
Gross NO_3^- immobilization (I_{NO3}, mg N kg^{-1} day^{-1})	Ln NO_3^-: NH_4^+ = – 0.318 × Ln I_{NO3} – 0.167	0.08	<0.0001	236
Dissimilatory nitrate reduction to ammonium (DNRA, mg N kg^{-1} day^{-1})	Ln NO_3^-: NH_4^+ = 0.117 × Ln DNRA + 0.771	0.01	0.200	135

Statistical significance (*p* value) was obtained by two-tailed Student’s t test. N is the number of observations.

Table S10. Simple linear regression equations for relationships between soil dissimilatory nitrate reduction to ammonium (DNRA, mg N kg^{-1} day^{-1}) and environmental factors (all analyses were based on transformed data except for soil pH).

**Variable/process**	**Equation**	R^2	***p***	**N**
Mean annual precipitation (MAP, mm)	Ln DNRA = 1.338 × Ln MAP – 11.989	0.17	<0.0001	171
Mean annual temperature (MAT, ºC)	Ln DNRA = 0.938 × Ln MAT – 4.824	0.05	0.003	172
Soil pH	Ln DNRA = 0.479 × pH – 4.898	0.12	<0.0001	177
Total carbon (C, g $kg^{–1}$)	Ln DNRA = 0.434 × Ln C – 3.703	0.05	0.001	192
Total nitrogen (N, g $kg^{–1}$)	Ln DNRA = 0.706 × Ln N – 2.795	0.15	<0.0001	187
Soil C:N	Ln DNRA = – 0.610 × Ln C:N – 0.849	0.01	0.109	191
Microbial biomass C (MBC, mg kg^{-1})	Ln DNRA = 0.762 × Ln MBC – 6.316	0.12	0.091	24
Microbial biomass N (MBN, mg kg^{-1})	Ln DNRA = 0.641 × Ln MBN – 3.858	0.09	0.106	32

Statistical significance (*p* value) was obtained by two-tailed Student's t test. N is the number of observations.

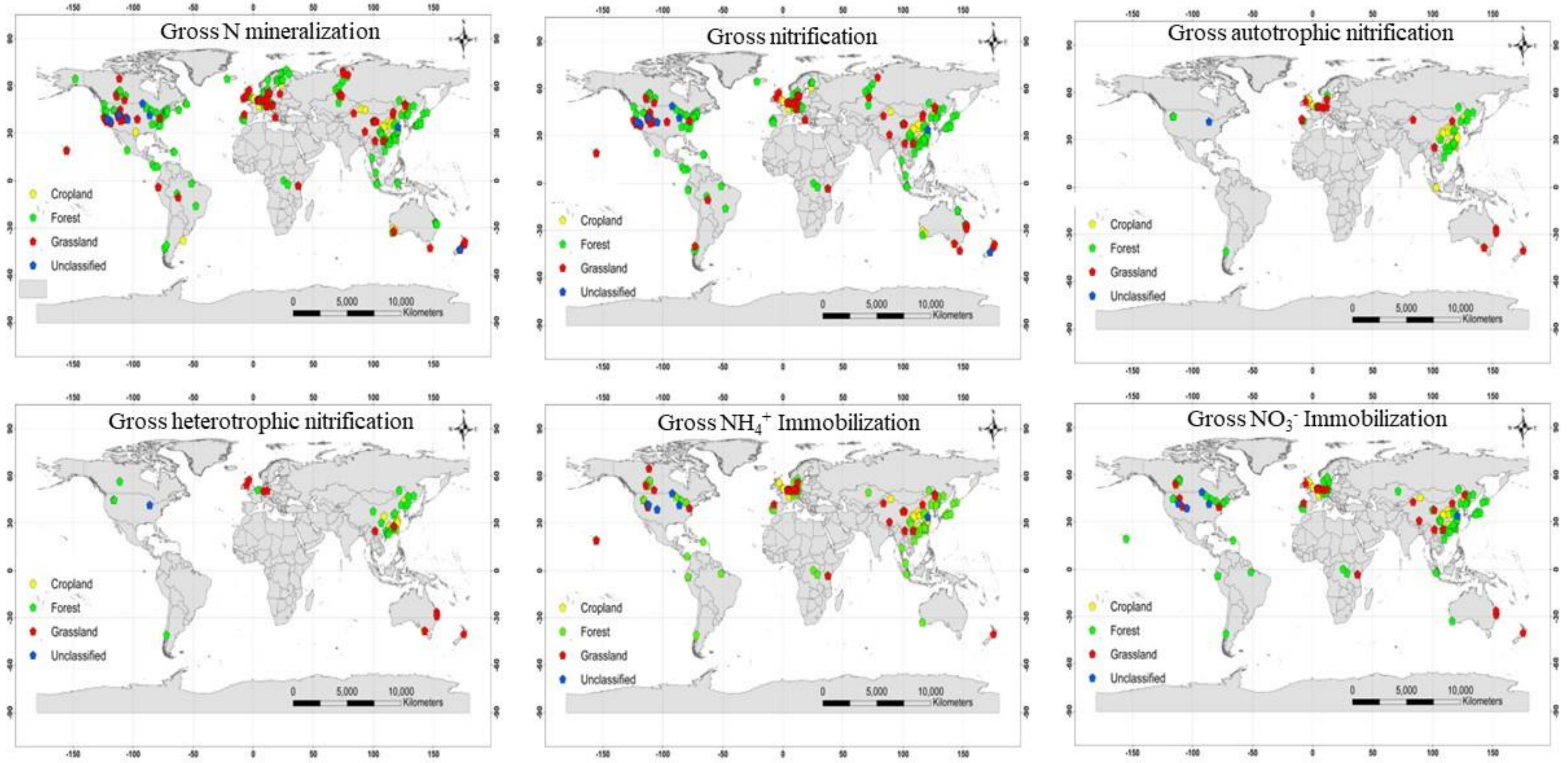

Fig. S1a. Global distribution of study sites included in our study for gross N mineralization, gross nitrification, gross autotrophic nitrification, gross heterotrophic nitrification, gross NH_4^+ immobilization, and gross NO_3^- immobilization.

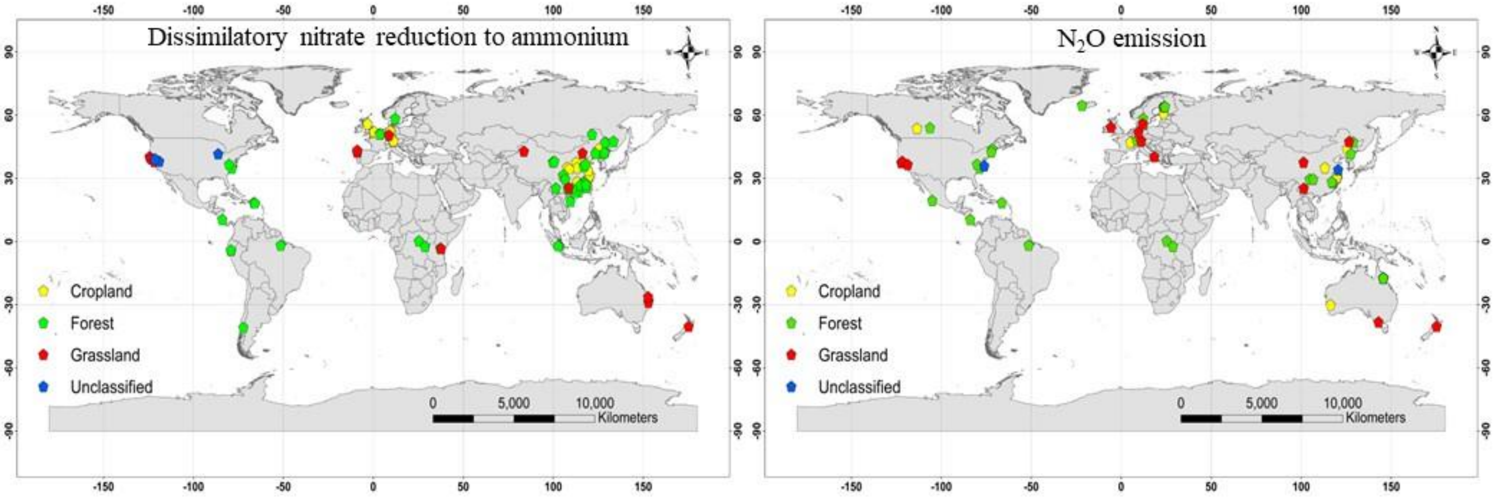

Fig. S1b. Global distribution of study sites included in our study for dissimilatory nitrate reduction to ammonium and N_2O emission.

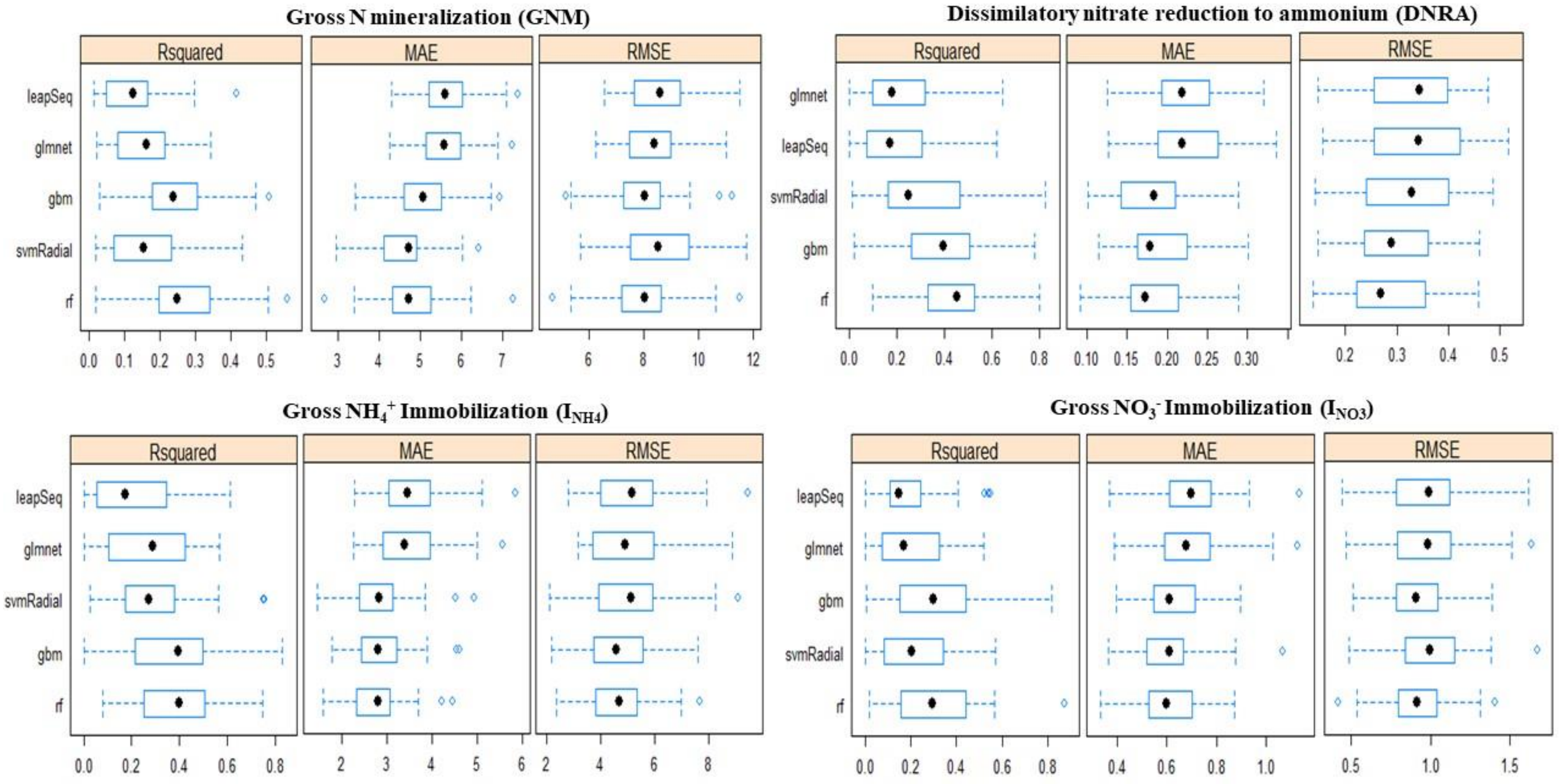

Fig. S2a. Results of 10-fold cross-validation of five predictive models [random forest (rf), generalized boosted regression models (gbm), support vector machine (svmRadial), stepwise regression (leapSeq), and generalized linear models (glmnet)] for gross N mineralization (n = 736), dissimilatory nitrate reduction to ammonium (n = 189), gross NH_4^+ immobilization (n = 356), and gross NO_3^- immobilization (n = 313). The regression coefficients of determination (R^2), the root mean square error (RMSE), and the mean of absolute value of errors (MAE) are three extensively used indicators for validation. The black point represents the average of R square, MAE, or RMSE. The box indicates the upper and lower quartiles, with the vertical lines representing the minimum and maximum values.

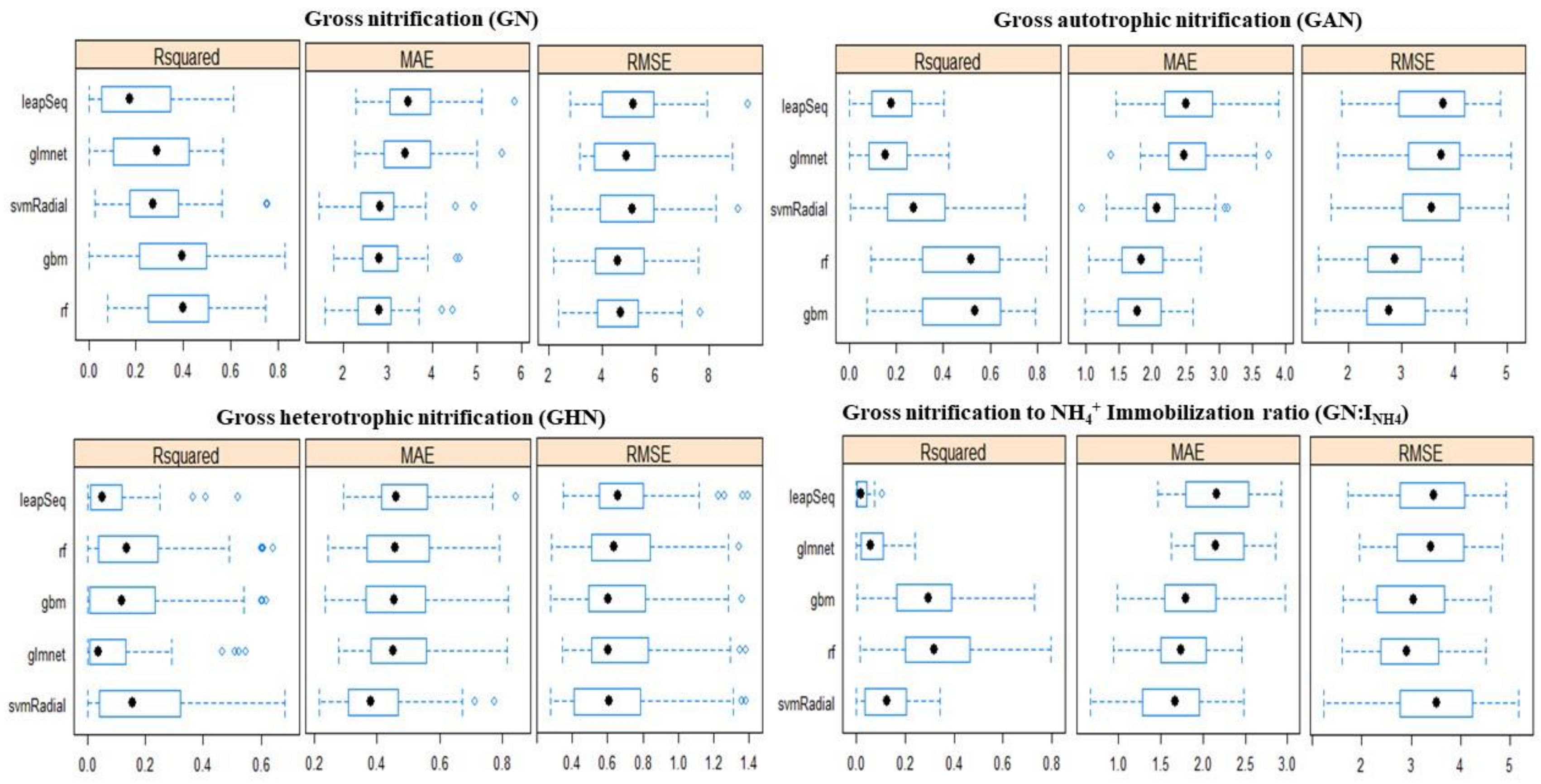

Fig. S2b. Results of 10-fold cross-validation of five predictive models [random forest (rf), generalized boosted regression models (gbm), support vector machine (svmRadial), stepwise regression (leapSeq), and generalized linear models (glmnet)] for gross nitrification (n = 600), gross autotrophic nitrification (n = 194), gross heterotrophic nitrification (n = 150), and gross nitrification to gross NH_4^+ immobilization (n = 332). The regression coefficients of determination (R^2), the root mean square error (RMSE), and the mean of absolute value of errors (MAE) are three extensively used indicators for validation. The black point represents the average of R square, MAE, or RMSE. The box indicates the upper and lower quartiles, with the vertical lines representing the minimum and maximum values.

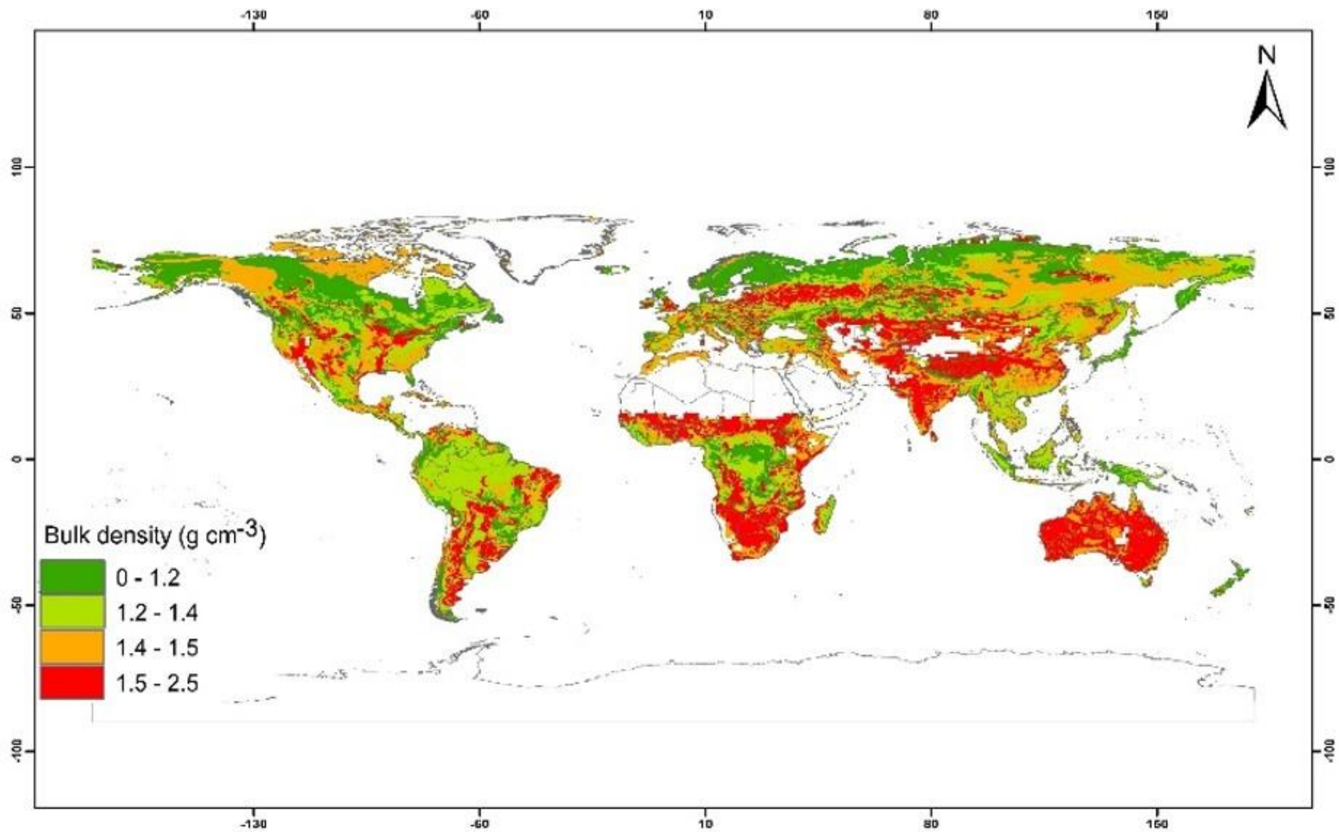

Fig. S3a. The spatial pattern of soil bulk density at the global scale.

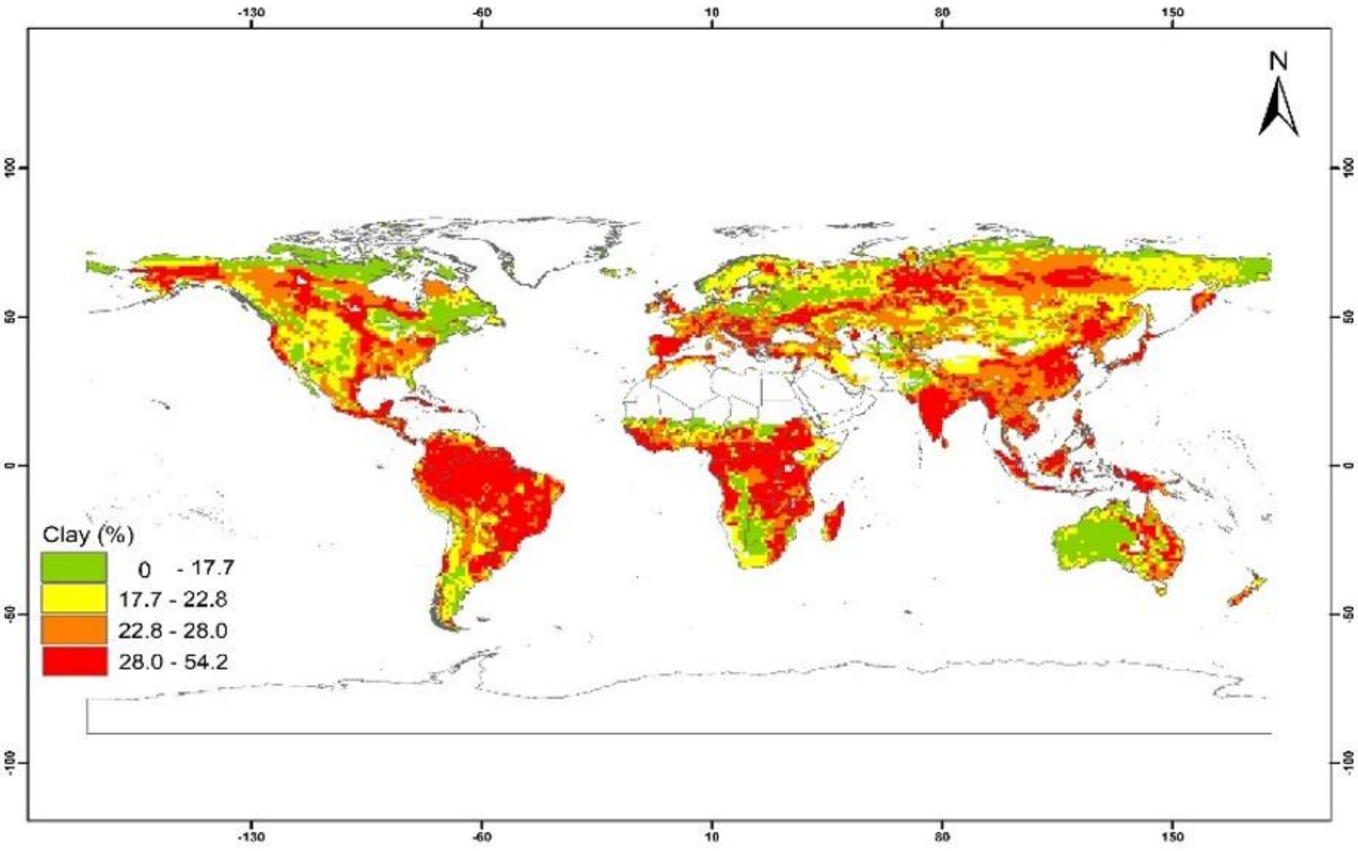

Fig. S3b. The spatial pattern of soil clay content at the global scale.

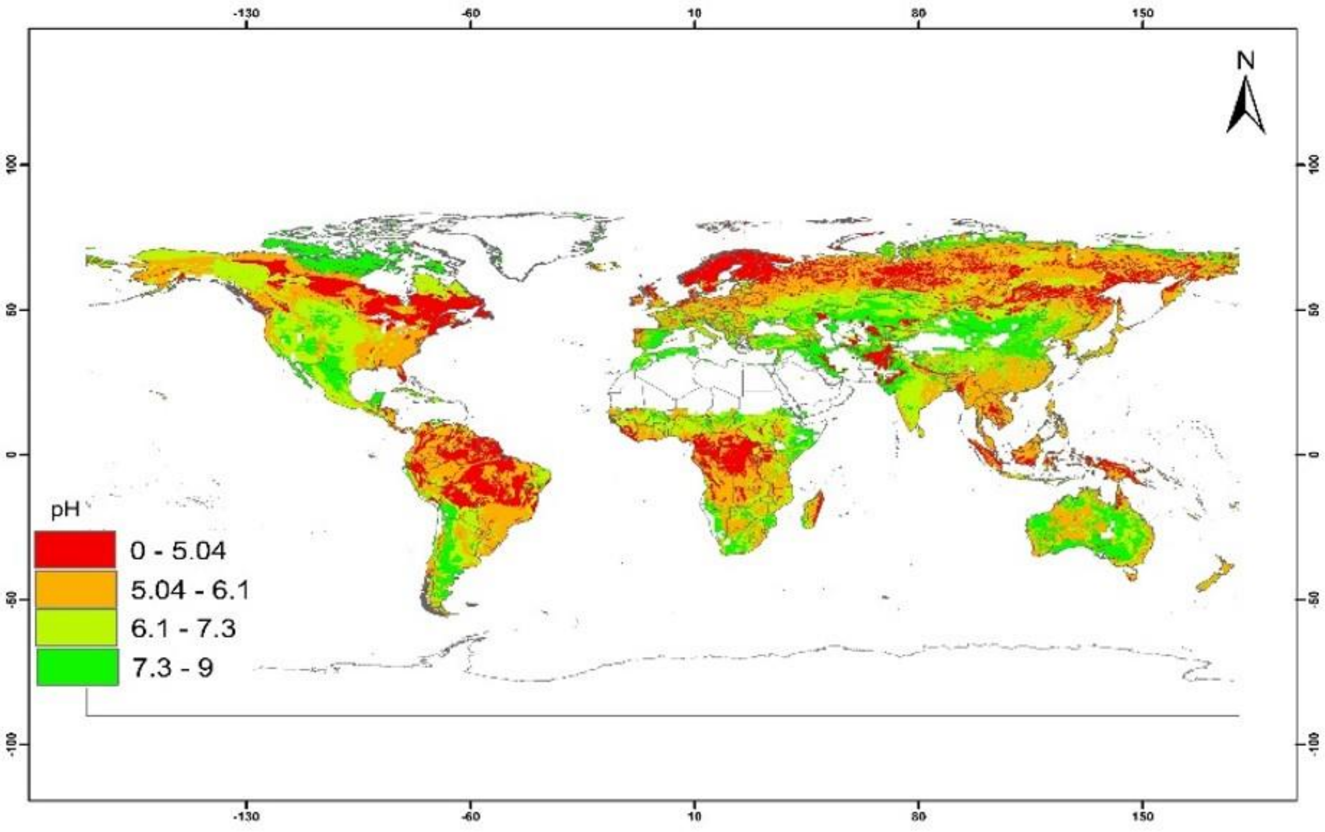

Fig. S3c. The spatial distribution pattern of soil pH at the global scale.

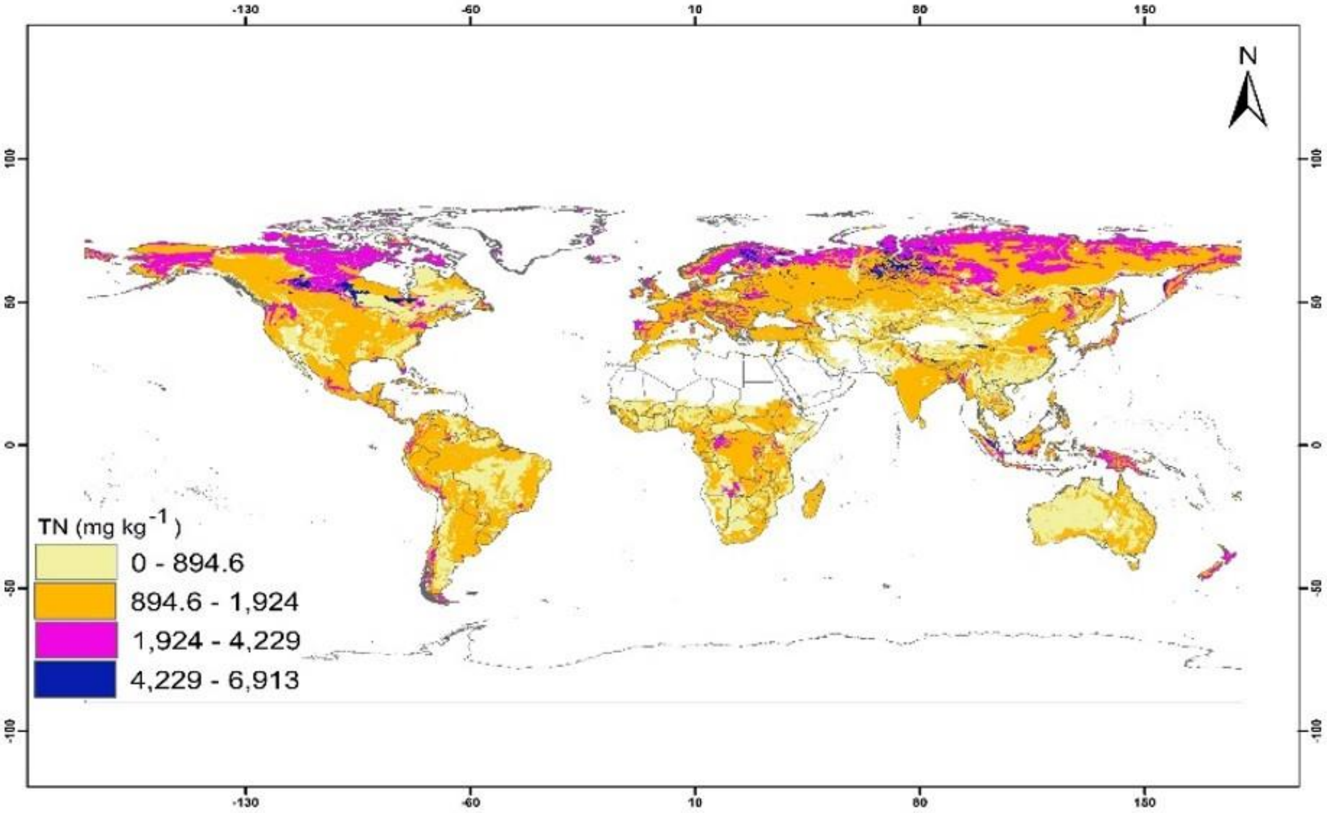

Fig. S3d. The spatial distribution pattern of soil total N at the global scale.

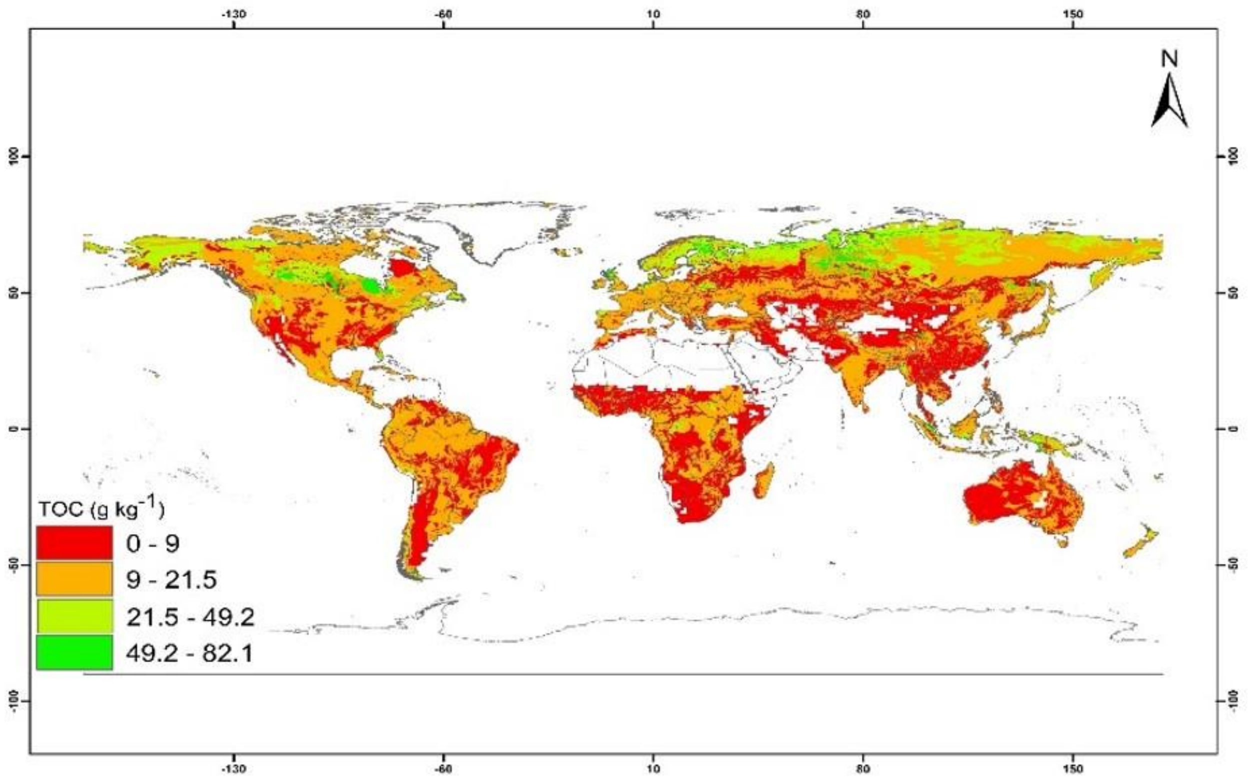

Fig. S3e. The spatial distribution pattern of soil total organic C at the global scale.

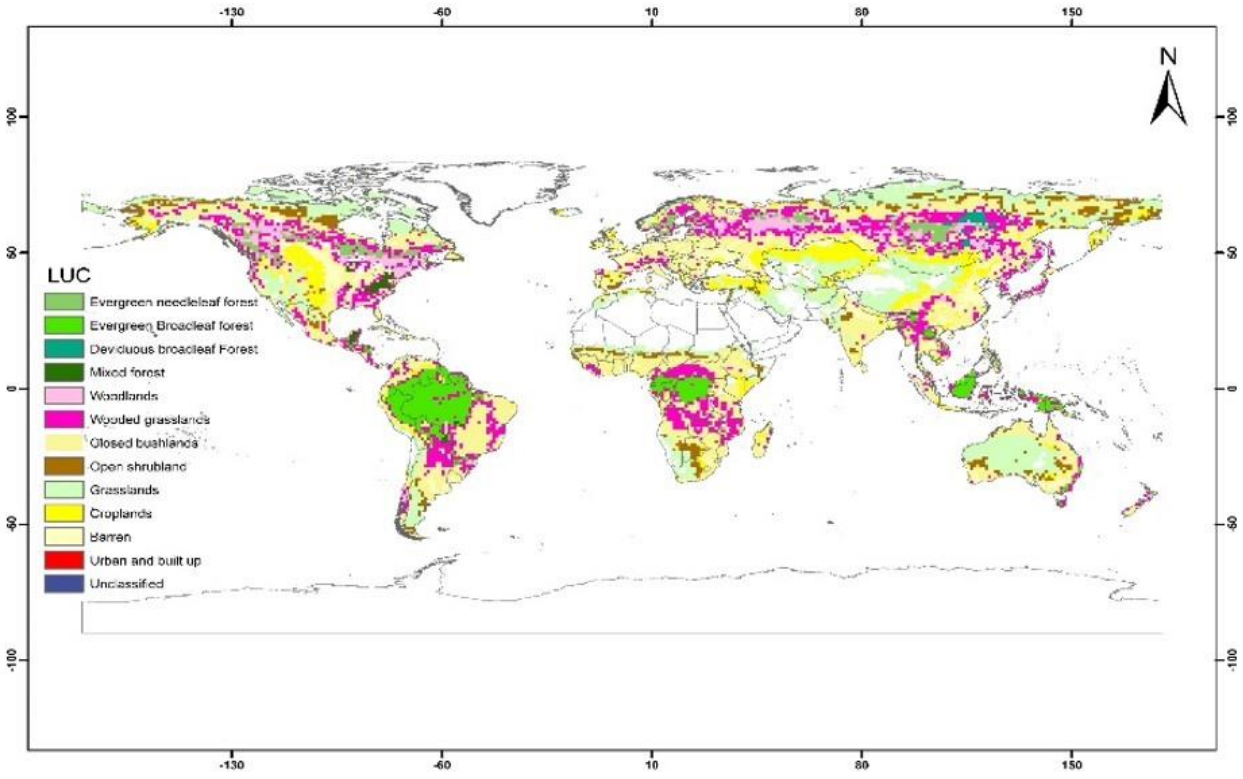

Fig. S3f. The spatial distribution pattern of land use at the global scale.

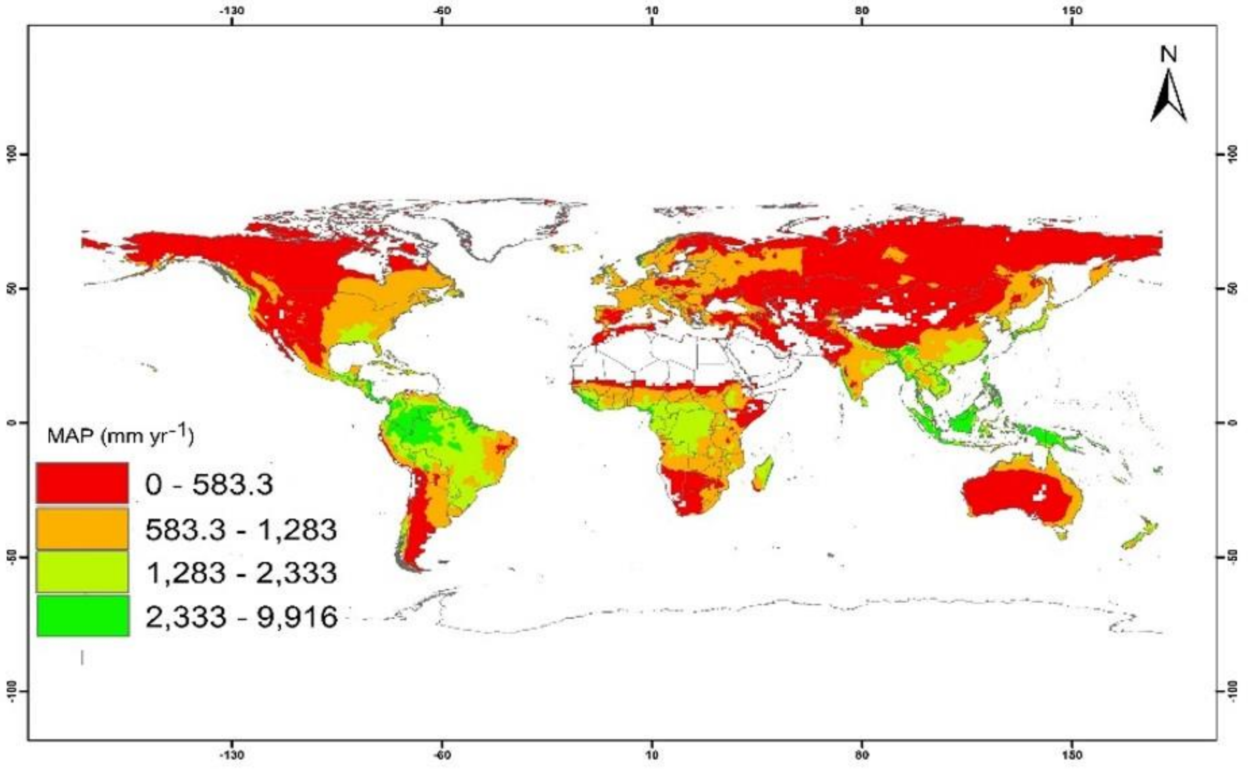

Fig. S3g. The spatial distribution pattern of mean annual precipitation (MAP) at the global scale.

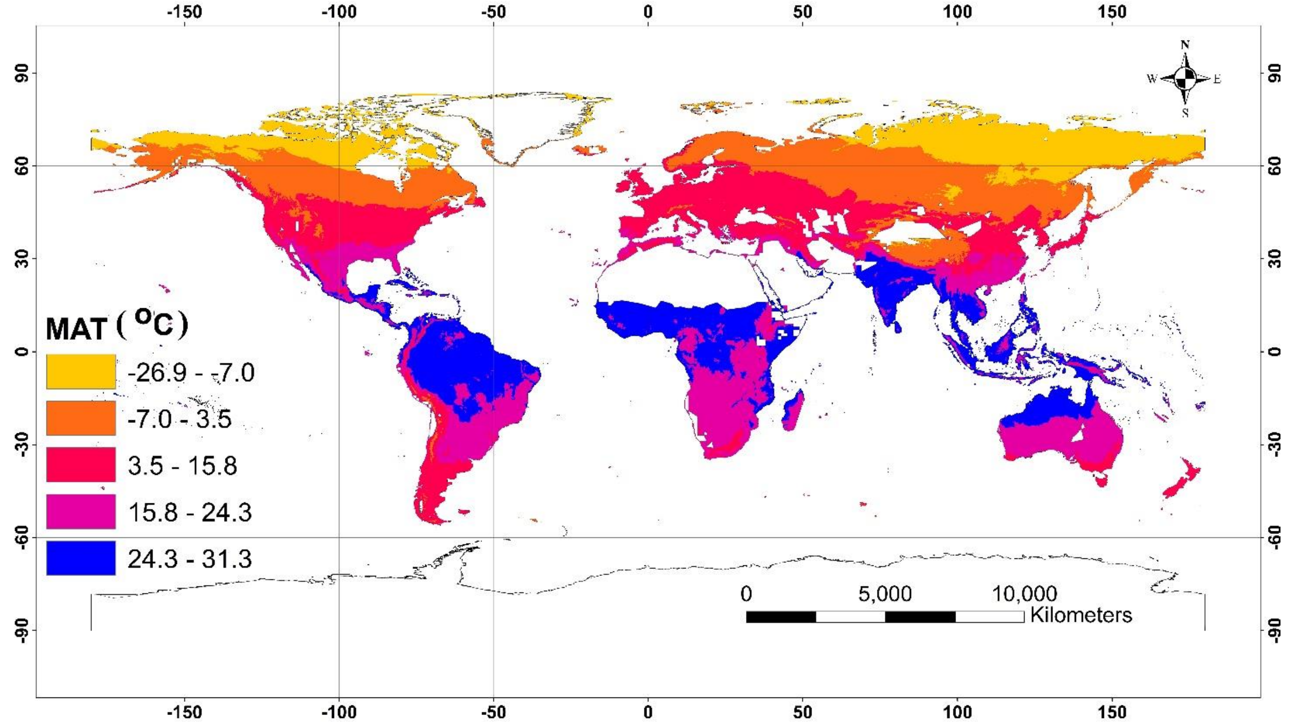

Fig. S3h. The spatial distribution pattern of mean annual temperature (MAT) at the global scale.

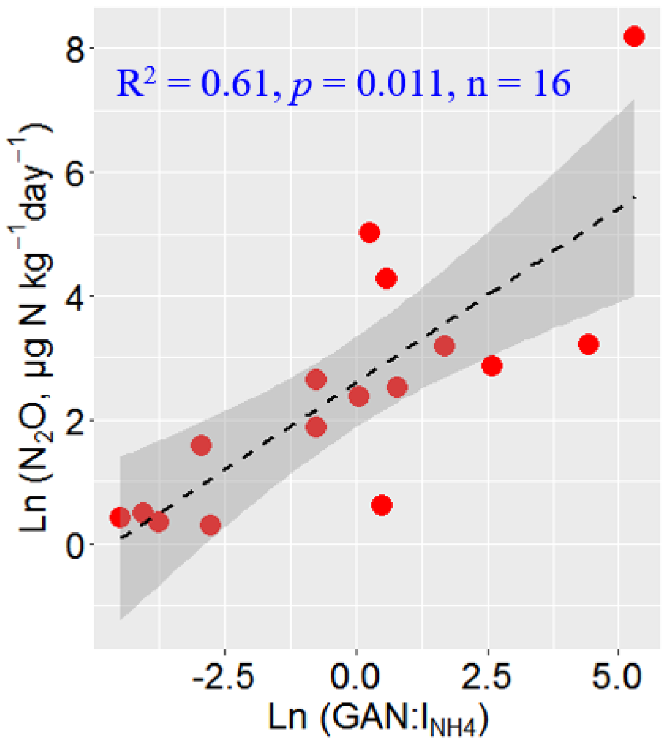

Fig. S4. Relationship of gross autotrophic nitrification to ammonium immobilization ratio (GAN:I_{NH4}) with nitrous oxide (N_2O) emissions at the global scale. The dashed lines are the slopes, and the gray area refers to the 95% confidence interval around the regression line. Statistical significance was obtained by two-tailed Student's t test. n is the number of observations.

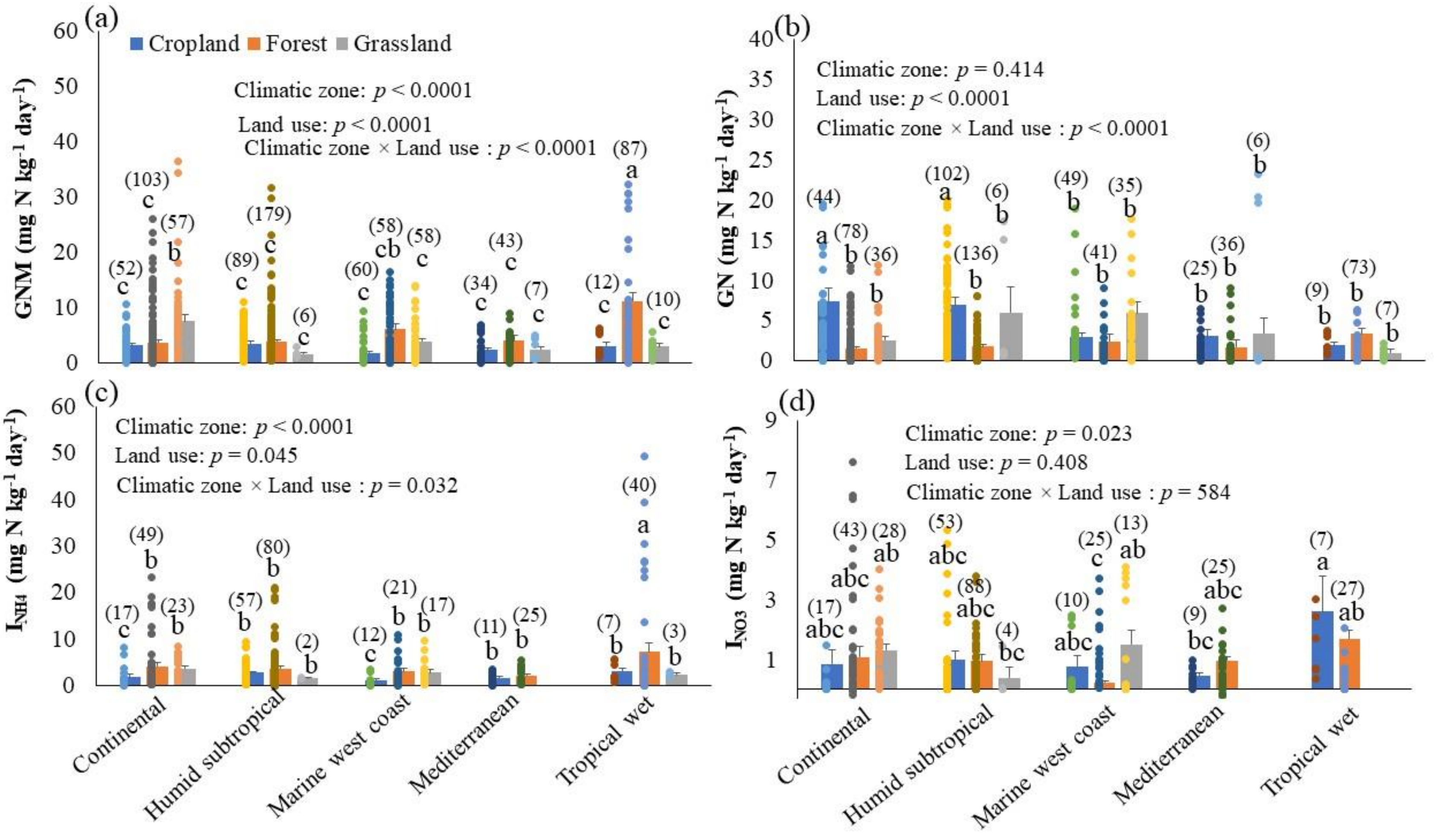

Fig. S5. Changes (means ± SE) of soil GNM (a), GN (b), I_{NH4} (c) , and I_{NO3} (d) with terrestrial ecosystems in different climatic zones. Differences in gross N transformation rates among ecosystem types were tested using two-way analysis of variance (ANOVA), with least significant differences (LSD). The different letters above bars denote significant differences at $p < 0.05$. Error bars represent the standard error of the means. Dot plots represent the corresponding data points. The numbers in parentheses are the number of observations (n). If the data did not show a normal distribution, a transformation to the natural logarithm (ln) was performed to approximate normality and stabilize the distribution. SE, standard error; GNM, gross N mineralization; GN, gross nitrification; I_{NH4}, ammonium immobilization; I_{NO3}, nitrate immobilization.

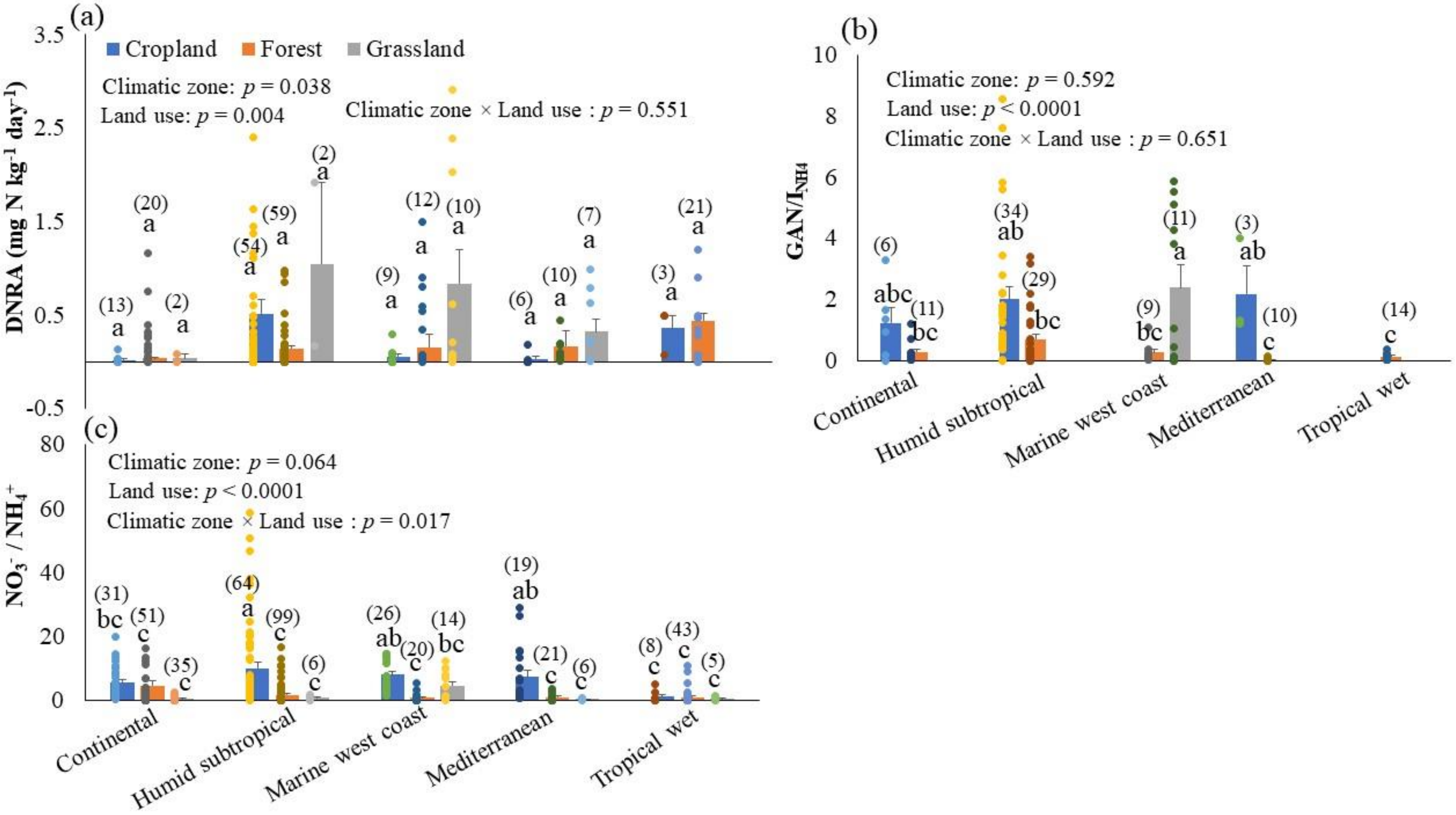

Fig. S6. Changes (means ± SE) of soil DNRA (a), NO_3^-/NH_4^+ ratio (b), and soil GN/I_{NH4} ratio (c) with terrestrial ecosystems in different climatic zones. Differences in DNRA, NO_3^-/NH_4^+ ratio, and soil GN/I_{NH4} ratio among ecosystem types were tested using two-way analysis of variance (ANOVA), with least significant differences (LSD). The different letters above bars denote significant differences at $p < 0.05$. Error bars represent the standard error of the means. Dot plots represent the corresponding data points. The numbers in parentheses are the number of observations (n). If the data did not show a normal distribution, a transformation to the natural logarithm (ln) was performed to approximate normality and stabilize the distribution. SE, standard error; DNRA, dissimilatory nitrate reduction to ammonium; I_{NH4}, ammonium immobilization; GAN, gross autotrophic nitrification.

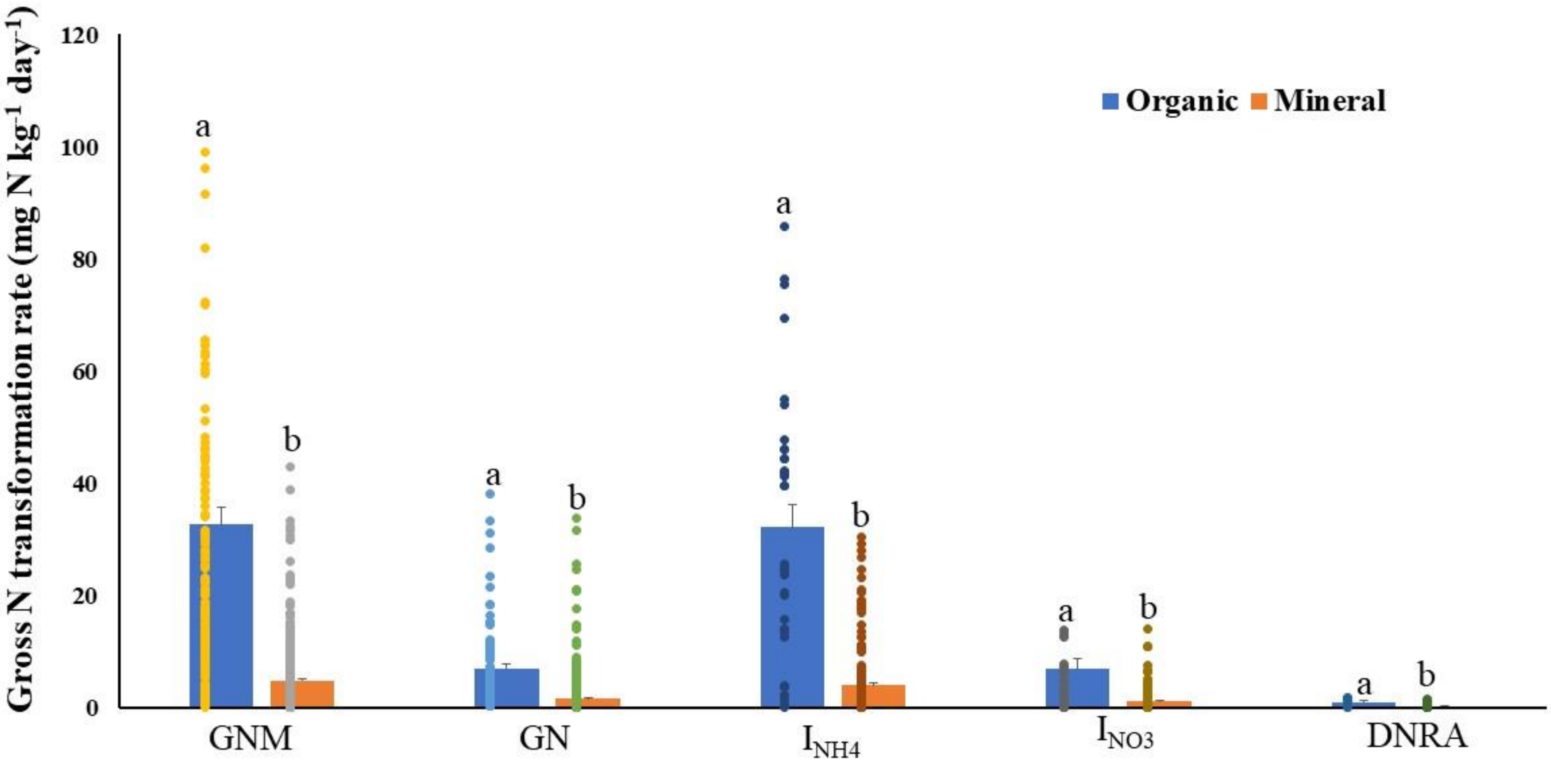

Fig. S7. Changes (means ± SE) of GNM (n = 396 and 137, p < 0.0001), GN (n = 365 and 85, p < 0.0001), I_{NH4} (n = 204 and 33, p < 0.0001), I_{NO3} (n = 198 and 33, p < 0.0001), and DNRA (n = 95 and 6, p < 0.0001) in mineral and organic layers of forest soils at the global scale, respectively. Difference in each gross N transformation rate among soil layers was tested using one-way analysis of variance (ANOVA), with least significant differences (LSD). The different letters above bars denote significant differences at p < 0.05. Error bars represent the standard error of the means. Dot plots represent the corresponding data points. If the data did not show a normal distribution, a transformation to the natural logarithm (ln) was performed to approximate normality and stabilize the distribution. SE, standard error; GNM, gross N mineralization; I_{NH4}, ammonium immobilization; I_{NO3}, nitrate immobilization; GN, gross nitrification; GAN, gross autotrophic nitrification; GHN, gross heterotrophic nitrification; DNRA, dissimilatory nitrate reduction to ammonium.

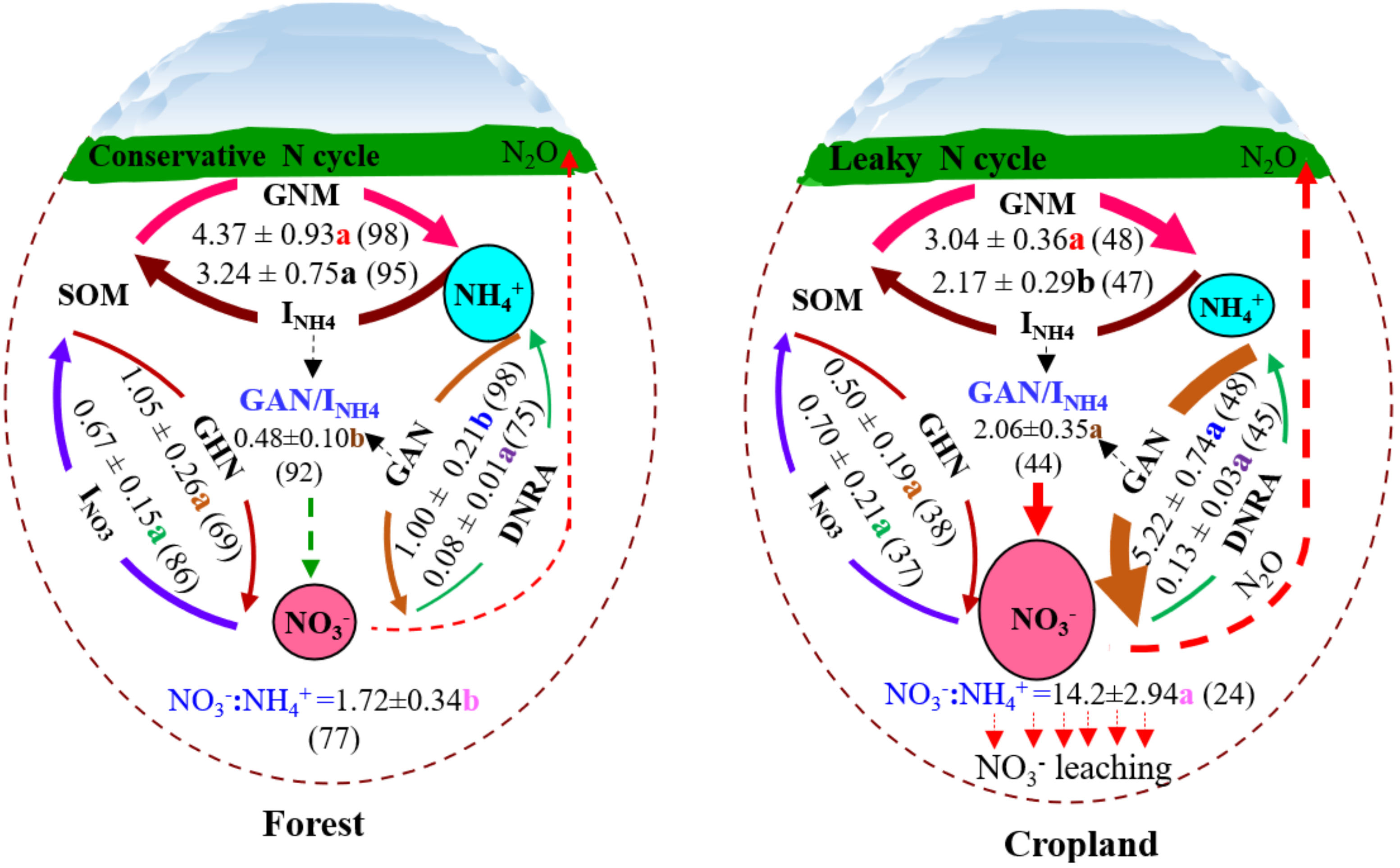

Fig. S8. Changes (means ± SE; mg N kg^{-1} day^{-1}) of gross N cycling in the mineral soil of forests and croplands (subset of data for sites that measured the full N cycling rates or most variables of soil N processes). Difference in each gross N transformation rate among terrestrial ecosystems was tested using one-way analysis of variance (ANOVA), with least significant differences (LSD). The different letters next to numbers indicate significant differences in gross N transformation rates across terrestrial ecosystems at $p < 0.05$, while the values in parentheses are the number of observations. The *p*-values were obtained by two-tailed tests. SE, standard error; GNM, gross N mineralization; I_{NH4}, ammonium immobilization; I_{NO3}, nitrate immobilization; GAN, gross autotrophic nitrification; GHN, gross heterotrophic nitrification; DNRA, dissimilatory nitrate reduction to ammonium; N_2O, nitrous oxide.

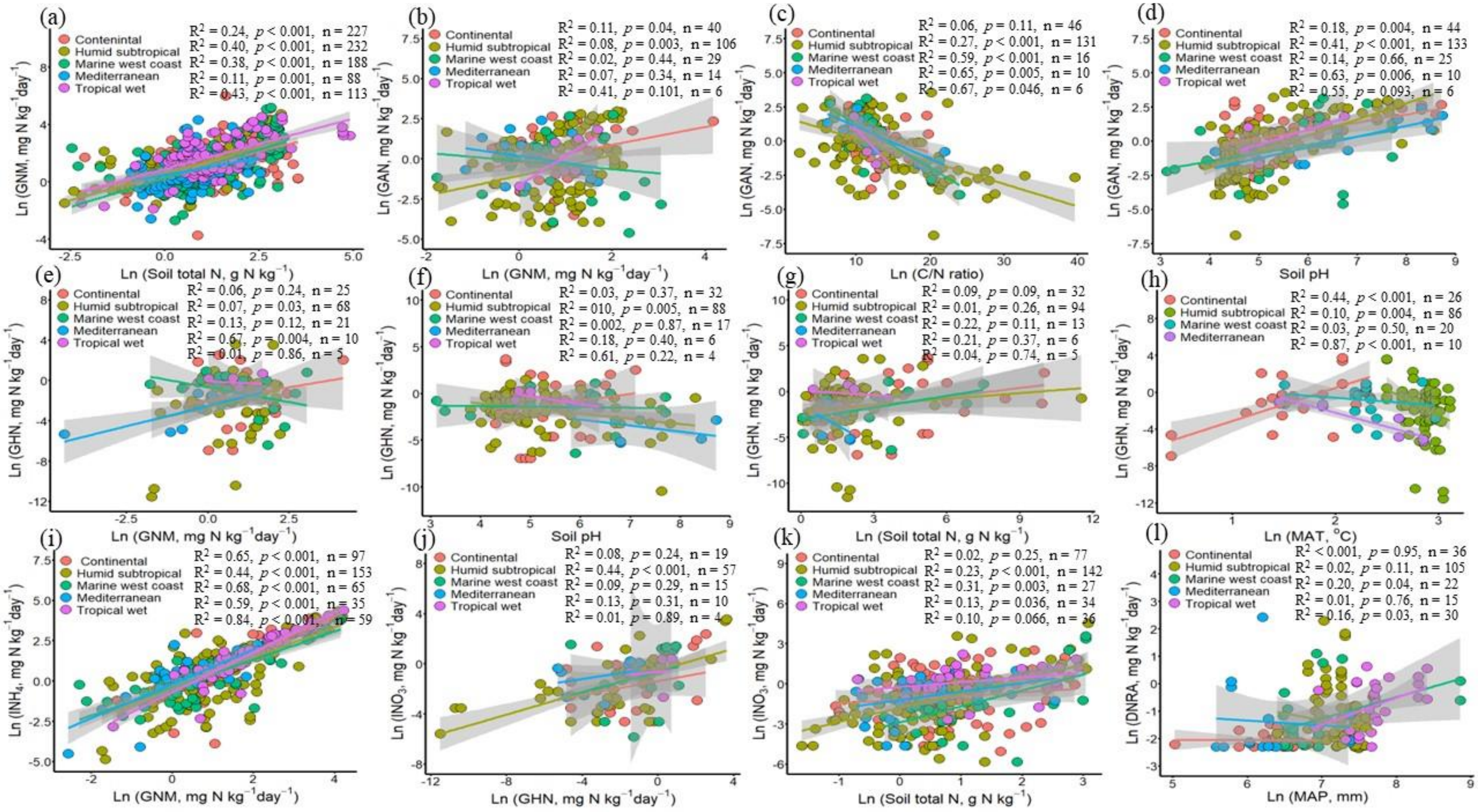

Fig. S9. Relationships of gross N transformation rates to each other and to environmental factors across climatic zones. The regression relationships between GNM and soil total N (a), GAN and GNM (b), GAN and C/N ratio (c), GAN and soil pH (d), GHN and GNM (e), GHN and soil pH (f), GHN and soil total N (g), GHN and MAT (h), I_{NH4} and GNM (i), I_{NO3} and GHN (j), I_{NO3} and soil total N (k), and DNRA and MAP (l). The solid lines are the slopes, and the gray area refers to the 95% confidence interval around the regression line. Statistical significance was obtained by two-tailed Student's t test. n is the number of observations. GNM, gross N mineralization; GI, gross N immobilization; I_{NH4}, ammonium immobilization; I_{NO3}, nitrate immobilization; GN, gross nitrification; GAN, gross autotrophic nitrification; GHN, gross heterotrophic nitrification; DNRA, dissimilatory nitrate reduction to ammonium; MAP, mean annual precipitation; MAT, mean annual temperature

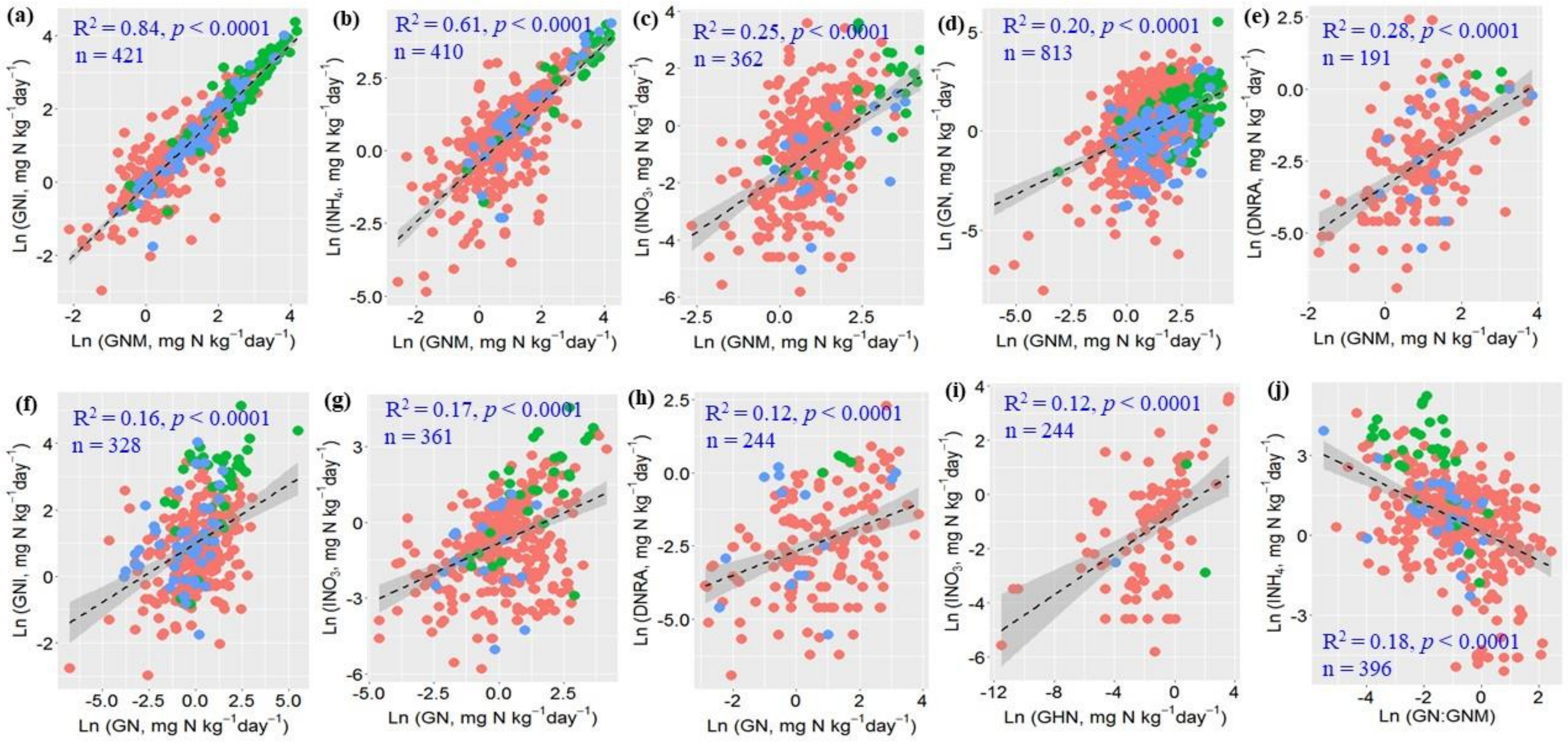

Fig. S10. Relationships of gross N transformation rates to each other at the global scale. **(a-e)** The regression relationship between GNM and GNI (a), I_{NH4} (b), I_{NO3} (c), GN (d), and DNRA (e). **(f-h)** The regression relationship between GN and GNI (f), I_{NO3} (g), and DNRA (h). **(i)** The regression relationship between GHN and I_{NO3}. **(j)** The regression relationship between GN:GNM and I_{NH4}. Data from mineral (red dots), organic (green dots), and mixed (mineral + organic; blue dots) soil layers were used. The dashed lines are the slopes, and the gray area refers to the 95% confidence interval around the regression line. Statistical significance was obtained by two-tailed Student's t test. GNM, gross N mineralization; GNI, gross N immobilization; I_{NH4}, ammonium immobilization; I_{NO3}, nitrate immobilization; GN, gross nitrification; GAN, gross autotrophic nitrification; GHN, gross heterotrophic nitrification; DNRA, dissimilatory nitrate reduction to ammonium.

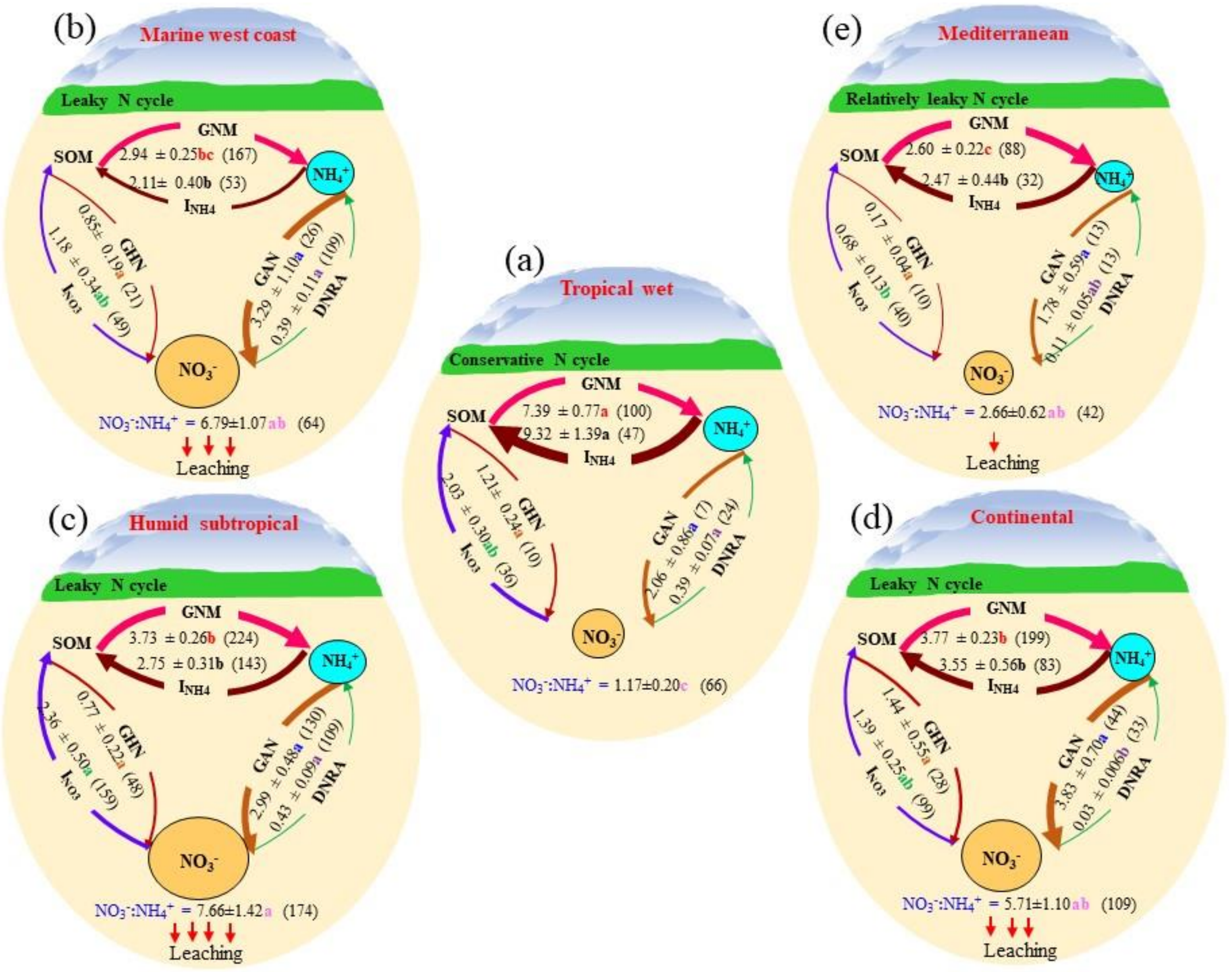

Fig. S11. Changes (mean± SE) of gross N cycling rates (mg N kg^{-1} day^{-1}) in tropical wet (a), marine west coast (b), humid subtropical (c), continental (d), and the Mediterranean (e) zones. Difference in GNM ($p < 0.0001$), I_{NH4} ($p < 0.0001$), I_{NO3} ($p = 0.023$), GAN ($p = 0.437$), GHN ($p = 0.374$), and DNRA ($p = 0.038$) rates among climatic zones was tested using one-way analysis of variance (ANOVA), with least significant differences (LSD). The different letters next to numbers indicate significant differences of gross N rate across climatic zones at $p < 0.05$, while the numbers in parentheses are the number of observations. The *p*-values were obtained by two-tailed tests. The comparisons among climatic zones were confined to mineral soil horizons data. SE, standard error; GNM, gross N mineralization; I_{NH4}, ammonium immobilization; I_{NO3}, nitrate immobilization; GN, gross nitrification; GAN, gross autotrophic nitrification; GHN, gross heterotrophic nitrification; DNRA, dissimilatory nitrate reduction to ammonium.

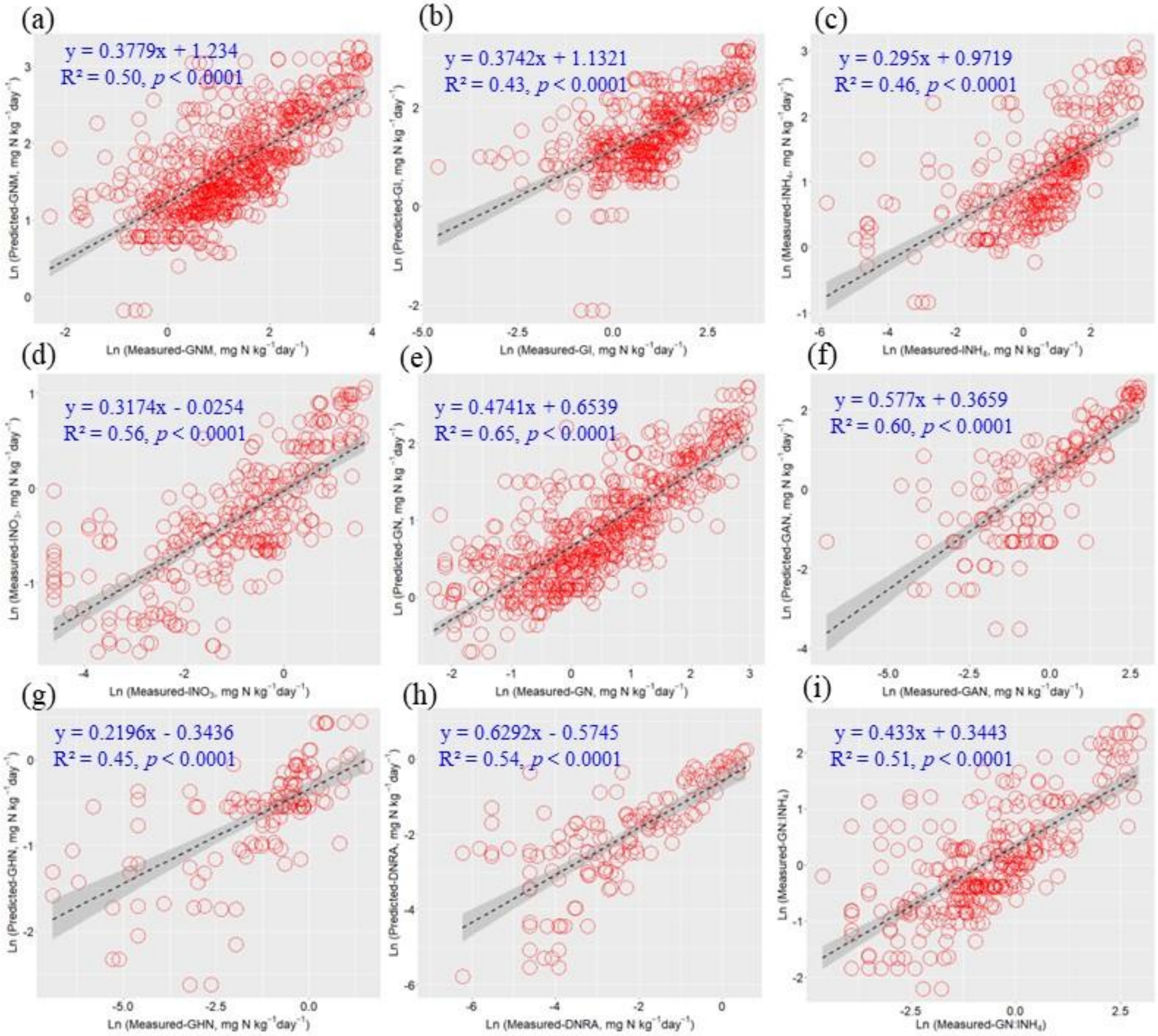

Fig. S12. Relationship between measured and predicted GNM (a, n = 736), GI (b, n = 455), I_{NH4} (c, n = 356), I_{NO3} (d, n = 313), GN (e, n = 600), GAN (f, n = 194), GHN (g, n = 150), DNRA (h, n = 189), and the ratio of GN:I_{NH4} (I, n = 332) at the global scale. The dashed lines are the slopes, and the gray area refers to the 95% confidence interval around the regression line. Statistical significance was obtained by two-tailed Student's t test. GNM, gross N mineralization; GI, gross N immobilization; I_{NH4}, ammonium immobilization; I_{NO3}, nitrate immobilization; GN, gross nitrification; GAN, gross autotrophic nitrification; GHN, gross heterotrophic nitrification; DNRA, dissimilatory nitrate reduction to ammonium.

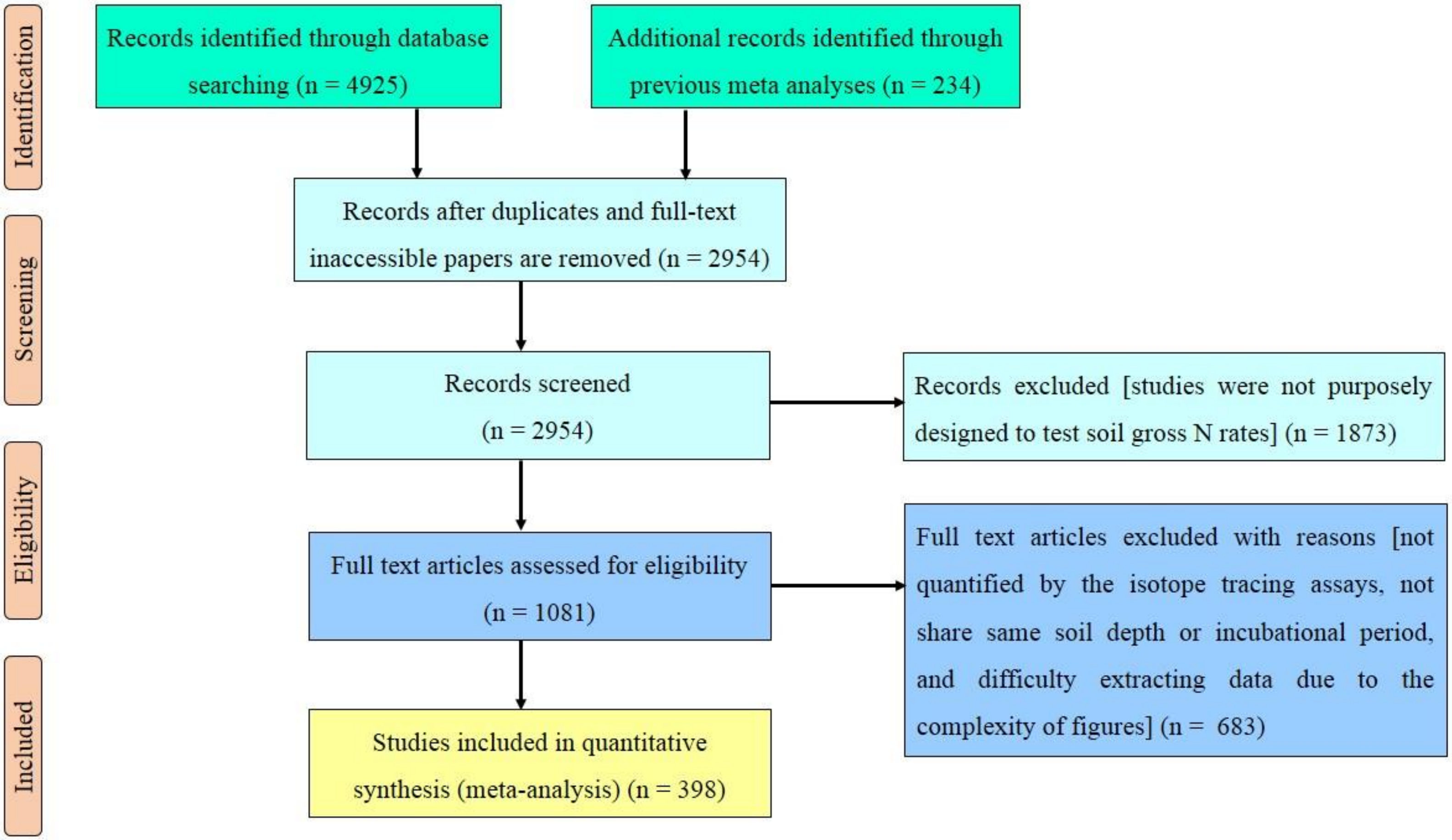

Fig. S13. A RISMA diagram showing the process involved for selection of publications included in this study.

Appendix. List of references containing data used in the analyses.

1. Allen, K., M.D. Corre, A. Tjoa and E. Veldkamp. 2015. Soil nitrogen-cycling responses to conversion of lowland forests to oil palm and rubber plantations in Sumatra, Indonesia. PloS One 10: e0133325.
2. Andersen, M.K and L.S Jensen. 2001. Low soil temperature effects on short-term gross N mineralisation–immobilisation turnover after incorporation of a green manure. Soil Biology and Biochemistry 33 (4-5), 511-521.
3. Anderson, C.I. and Poth, M.A. 1998. Controls on Fluxes of Trace Gases from Brazilian Cerrado Soils. Journal of Environment Quality, 27(5), 1117–1124.
4. Andresen, L., S. Bode, A. Tietema, P. Boeckx and T. Rütting. 2015. Amino acid and N mineralization dynamics in heathland soil after long-term warming and repetitive drought. Soil 1: 341-349.
5. Andresen, L.C., A.-K. Björsne, S. Bodé, L. Klemedtsson, P. Boeckx and T. Rütting. 2016. Simultaneous quantification of depolymerization and mineralization rates by a novel ^{15}N tracing model. Soil 2: 433-442.
6. Andresen, L.C., Y. Carrillo, C.A. Macdonald, L. Castañeda-Gómez, S. Bode and T. Rütting. 2020. Nitrogen dynamics after two years of elevated CO_2 in phosphorus limited Eucalyptus woodland. Biogeochemistry 150: 297-312.
7. Arnold, J., M.D. Corre and E. Veldkamp. 2008. Cold storage and laboratory incubation of intact soil cores do not reflect in-situ nitrogen cycling rates of tropical forest soils. Soil Biology and Biochemistry 40: 2480-2483.
8. Attard, E., X. Le Roux, X. Charrier, O. Delfosse, N. Guillaumaud, G. Lemaire, et al. 2016. Delayed and asymmetric responses of soil C pools and N fluxes to grassland/cropland conversions. Soil Biology and Biochemistry 97: 31-39.
9. Baldos, A.P., M.D. Corre and E. Veldkamp. 2015. Response of N cycling to nutrient inputs in forest soils across a 1000–3000 m elevation gradient in the Ecuadorian Andes. Ecology 96: 749-761.
10. Ball, P., M. MacKenzie, T. DeLuca. and W.H. Montana. 2010. Wildfire and charcoal enhance nitrification and ammonium-oxidizing bacterial abundance in dry Montane forest soils. Journal of Environmental Quality 39(4), 1243-1253 .
11. Banning, N., C. Grant, D. Jones and D. Murphy. 2008. Recovery of soil organic matter, organic matter turnover and nitrogen cycling in a post-mining forest rehabilitation chronosequence. Soil Biology and Biochemistry 40: 2021-2031.
12. Barraclough, D. and G. Puri. 1995. The use of ^{15}N pool dilution and enrichment to separate the heterotrophic and autotrophic pathways of nitrification. Soil Biology and Biochemistry 27: 17-22.
13. Bauters, M., H. Verbeeck, T. Rütting, M. Barthel, B. Bazirake Mujinya, F. Bamba, et al. 2019. Contrasting nitrogen fluxes in African tropical forests of the Congo Basin. Ecological Monographs 89: e01342.
14. Bedard-Haughn, A., L.-P. Comeau and A. Sangster. 2013. Gross nitrogen mineralization in pulse-crop rotations on the Northern Great Plains. Nutrient Cycling in Agroecosystems 95: 159-174.

15. Bedard-Haughn, A., A.L. Matson, and D.J. Pennock. 2006. Land use effects on gross nitrogen mineralization, nitrification, and N_2O emissions in ephemeral wetlands. Soil Biology and Biochemistry 38(12): 3398-3406.
16. Bengtson, P., U., Falkengren-Grerup, G., Bengtsson and H. Setälä. 2005. Relieving Substrate Limitation-Soil Moisture and Temperature Determine Gross N Transformation Rates. Oikos 111(1), 81–90.
17. Bengtsson, G., P. Bengtson and K.F. Månsson. 2003. Gross nitrogen mineralization-, immobilization-, and nitrification rates as a function of soil C/N ratio and microbial activity. Soil Biology and Biochemistry 35: 143-154.
18. Björsne, A.-K., T. Rütting and P. Ambus. 2014. Combined climate factors alleviate changes in gross soil nitrogen dynamics in heathlands. Biogeochemistry 120: 191-201.
19. Booth, M.S., J.M. Stark and M.M. Caldwell. 2003. Inorganic N turnover and availability in annual-and perennial-dominated soils in a northern Utah shrub-steppe ecosystem. Biogeochemistry 66: 311-330.
20. Booth, M.S., J.M. Stark and S.C. Hart. 2006. Soil-mixing effects on inorganic nitrogen production and consumption in forest and shrubland soils. Plant and Soil 289 (1-2), 5–15.
21. Bowles, T.M., P.A. Raab and L.E. Jackson. 2015. Root expression of nitrogen metabolism genes reflects soil nitrogen cycling in an organic agroecosystem. Plant and Soil 392: 175-189.
22. Boyle, S.A., R.R. Yarwood, P.J. Bottomley and D.D. Myrold. 2008. Bacterial and fungal contributions to soil nitrogen cycling under Douglas fir and red alder at two sites in Oregon. Soil Biology and Biochemistry 40: 443-451.
23. Bradley, R., B. Titus and J. Fyles. 1997. Nitrogen acquisition and competitive ability of Kalmia angustifolia L., paper birch (*Betula papyrifera* Marsh.) and black spruce (*Picea mariana* (Mill.) BSP) seedlings grown on different humus forms. Plant and soil 195: 209-220.
24. Bradley, R., B. Titus and C.Preston. 2000. Changes to mineral N cycling and microbial communities in black spruce humus after additions of $(NH_4)_2SO_4$ and condensed tannins extracted from Kalmia angustifolia and balsam fir. Soil Biology and Biochemistry 32: 1227-1240.
25. Braun, J., M. Mooshammer, W. Wanek, J. Prommer, T.W. Walker, T. Rütting, et al. 2018. Full ^{15}N tracer accounting to revisit major assumptions of ^{15}N isotope pool dilution approaches for gross nitrogen mineralization. Soil Biology and Biochemistry 117: 16-26.
26. Breuer, L. and K. Butterbach-Bahl. 2005. Local temperature optimum of N_2O production rates in tropical rain forest soils of Australia. Soil Research 43(6): 689-694.
27. Breuer, L., Kiese, R. and K. Butterbach-Bahl. 2002. Temperature and moisture effects on nitrification rates in tropical rain-forest soils. Soil Science Society of America Journal, 66(3): 834-844.
28. Buckeridge, K.M., E. Zufelt, H. Chu and P. Grogan. 2010. Soil nitrogen cycling rates in low arctic shrub tundra are enhanced by litter feedbacks. Plant and Soil 330: 407-421.
29. Burger, M. and L.E. Jackson. 2003. Microbial immobilization of ammonium and nitrate in relation to ammonification and nitrification rates in organic and conventional cropping systems. Soil Biology and Biochemistry 35: 29-36.
30. Burton, J., C. Chen, Z. Xu and H. Ghadiri. 2007. Gross nitrogen transformations in adjacent native and plantation forests of subtropical Australia. Soil Biology and Biochemistry 39: 426-433.

31. Campbell, J.L. and S.T. Gower. 2000. Detritus production and soil N transformations in old-growth eastern hemlock and sugar maple stands. Ecosystems 3(2): 185-192.
32. Carmosini, N., K. Devito and E. Prepas. 2002. Gross nitrogen transformations in harvested and mature aspen-conifer mixed forest soils from the Boreal Plain. Soil Biology and Biochemistry 34: 1949-1951.
33. Case, S.D., N.P. McNamara, D.S. Reay, A.W. Stott, H.K. Grant and J. Whitaker. 2015. Biochar suppresses N_2O emissions while maintaining N availability in a sandy loam soil. Soil Biology and Biochemistry 81: 178-185.
34. Castellano, M.J., J.P. Kaye, H. Lin and J.P. Schmidt. 2012. Linking carbon saturation concepts to nitrogen saturation and retention. Ecosystems 15: 175-187.
35. Castellano-Hinojosa, A., A.F. Charteris, C. Müller, A. Jansen-Willems, J. González-López, E.J. Bedmar, et al. 2020. Occurrence and ^{15}N-quantification of simultaneous nitrification and denitrification in N-fertilised soils incubated under oxygen-limiting conditions. Soil Biology and Biochemistry 143: 107757.
36. Chen, J. and J.M. Stark. 2000. Plant species effects and carbon and nitrogen cycling in a sagebrush–crested wheatgrass soil. Soil Biology and Biochemistry 32: 47-57.
37. Chen, Y. and P. Högberg. 2006. Gross nitrogen mineralization rates still high 14 years after suspension of N input to a N-saturated forest. Soil Biology and Biochemistry 38: 2001-2003.
38. Chen, Z., J. Zhang, Z. Xiong, G. Pan and C. Müller. 2016. Enhanced gross nitrogen transformation rates and nitrogen supply in paddy field under elevated atmospheric carbon dioxide and temperature. Soil Biology and Biochemistry 94: 80-87.
39. Chen, Z., W. Ding, Y. Xu, C. Müller, T. Rütting, H. Yu, et al. 2015. Importance of heterotrophic nitrification and dissimilatory nitrate reduction to ammonium in a cropland soil: evidences from a ^{15}N tracing study to literature synthesis. Soil Biology and Biochemistry 91: 65-75.
40. Cheng, Y., J. Wang, B. Mary, J.-b. Zhang, Z.-c. Cai and S.X. Chang. 2013a. Soil pH has contrasting effects on gross and net nitrogen mineralizations in adjacent forest and grassland soils in central Alberta, Canada. Soil Biology and Biochemistry 57: 848-857.
41. Cheng, Y., J. Wang, J.-B. Zhang, B. Mary and Z.-C. Cai. 2014c. The mechanisms behind reduced NH_4^+ and NO_3^- accumulation due to litter decomposition in the acidic soil of subtropical forest. Plant and Soil 378: 295-308.
42. Cheng, Y., J. Wang, J.-B. Zhang, S.-Q. Wang and Z.-C. Cai. 2015a. The different temperature sensitivity of gross N transformations between the coniferous and broad-leaved forests in subtropical China. Soil Science and Plant Nutrition 61: 506-515.
43. Cheng, Y., J. Wang, N. Sun, M. Xu, J. Zhang, Z. Cai, et al. 2018. Phosphorus addition enhances gross microbial N cycling in phosphorus-poor soils: a ^{15}N study from two long-term fertilization experiments. Biology and Fertility of Soils 54: 783-789.
44. Cheng, Y., J. Wang, S.-Q. Wang, J.-B. Zhang and Z.-C. Cai. 2014d. Effects of soil moisture on gross N transformations and N_2O emission in acid subtropical forest soils. Biology and Fertility of Soils 50: 1099-1108.
45. Cheng, Y., J. Zhang, J. Zhu, G. Liu, C. Zhu and S. Wang. 2016a. Ten years of elevated atmospheric CO_2 does not alter soil nitrogen availability in a rice paddy. Soil Biology and Biochemistry 98: 99-108.

46. Cheng, Y., J.-B. Zhang, C. Müller and S.-Q. Wang. 2015b. ^{15}N tracing study to understand the N supply associated with organic amendments in a vineyard soil. Biology and Fertility of Soils 51: 983-993.
47. Cheng, Y., J. Wang, J.-B. Zhang, C. Müller and S.-Q. Wang. 2015c. Mechanistic insights into the effects of N fertilizer application on N_2O-emission pathways in acidic soil of a tea plantation. Plant and Soil 389(1-2): 45-57.
48. Cheng, Y., Y. Cai and S.-q. Wang. 2016. Yak and Tibetan sheep dung return enhance soil N supply and retention in two alpine grasslands in the Qinghai-Tibetan Plateau. Biology and Fertility of Soils 52: 413-422.
49. Cheng, Y., Z. Cai, S.X. Chang, J. Wang and J. Zhang. 2013b. Effects of soil pH and salt on N_2O production in adjacent forest and grassland soils in central Alberta, Canada. Journal of Soils and Sediments 13: 863-868.
50. Cheng, Y., Z.-c. Cai, J.-b. Zhang and S.X. Chang. 2011. Gross N transformations were little affected by 4 years of simulated N and S depositions in an aspen-white spruce dominated boreal forest in Alberta, Canada. Forest Ecology and Management 262: 571-578.
51. Cheng, Y., Z.-c. Cai, J.-b. Zhang, M. Lang, B. Mary and S.X. Chang. 2012a. Soil moisture effects on gross nitrification differ between adjacent grassland and forested soils in central Alberta, Canada. Plant and Soil 352: 289-301.
52. Cheng, Y., Z.-c. Cai, S.X. Chang, J. Wang and J.-b. Zhang. 2012b. Wheat straw and its biochar have contrasting effects on inorganic N retention and N_2O production in a cultivated Black Chernozem. Biology and Fertility of Soils 48: 941-946.
53. Christenson, L., G. Lovett, K. Weathers and M. Arthur. 2009. The influence of tree species, nitrogen fertilization, and soil C to N ratio on gross soil nitrogen transformations. Soil Science Society of America Journal 73: 638-646.
54. Clark, D.R., B.A. McKew, L.F. Dong, G. Leung, A.J. Dumbrell, A. Stott, et al. 2020. Mineralization and nitrification: Archaea dominate ammonia-oxidising communities in grassland soils. Soil Biology and Biochemistry 143: 107725.
55. Coban, O., Kuschk, P., Kappelmeyer, U., Spott, O., Martienssen, M., Jetten, M.S. and Knoeller, K., 2015. Nitrogen transforming community in a horizontal subsurface-flow constructed wetland. Water Research 74: 203-212.
56. Compton, J.E. and R.D. Boone. 2002. Soil nitrogen transformations and the role of light fraction organic matter in forest soils. Soil Biology and Biochemistry 34: 933-943.
57. Cookson, W., I. Cornforth and J. Rowarth. 2002. Winter soil temperature (2–15 ºC) effects on nitrogen transformations in clover green manure amended or unamended soils; a laboratory and field study. Soil Biology and Biochemistry 34: 1401-1415.
58. Cookson, W., M. Osman, P. Marschner, D. Abaye, I. Clark, D. Murphy, et al. 2007. Controls on soil nitrogen cycling and microbial community composition across land use and incubation temperature. Soil Biology and Biochemistry 39: 744-756.
59. Cookson, W., P. Marschner, I. Clark, N. Milton, M. Smirk, D. Murphy, et al. 2006. The influence of season, agricultural management, and soil properties on gross nitrogen transformations and bacterial community structure. Soil Research 44: 453-465.
60. Cookson, William R., et al. 2005. The contribution of soil organic matter fractions to carbon and nitrogen mineralization and microbial community size and structure. Soil Biology and Biochemistry 37(9): 1726-1737.

61. Corre, M.D., E. Veldkamp, J. Arnold and S.J. Wright. 2010. Impact of elevated N input on soil N cycling and losses in old-growth lowland and montane forests in Panama. Ecology 91: 1715-1729.
62. Corre, M.D., F.O. Beese and R. Brumme. 2003. Soil nitrogen cycle in high nitrogen deposition forest: changes under nitrogen saturation and liming. Ecological Applications 13: 287-298.
63. Corre, M.D., G. Dechert and E. Veldkamp. 2006. Soil nitrogen cycling following montane forest conversion in central Sulawesi, Indonesia. Soil Science Society of America Journal 70: 359-366.
64. Corre, M.D., J.P. Sueta and E. Veldkamp. 2014. Nitrogen-oxide emissions from tropical forest soils exposed to elevated nitrogen input strongly interact with rainfall quantity and seasonality. Biogeochemistry 118: 103-120.
65. Corre, M.D., R. Brumme, E. Veldkamp and F.O. Beese. 2007. Changes in nitrogen cycling and retention processes in soils under spruce forests along a nitrogen enrichment gradient in Germany. Global Change Biology 13: 1509-1527.
66. Corre, M.D., R.R. Schnabel and W.L. Stout. 2002. Spatial and seasonal variation of gross nitrogen transformations and microbial biomass in a Northeastern US grassland. Soil Biology and Biochemistry 34(4): 445-457.
67. Daebeler, A., P.L. Bodelier, M.M. Hefting, T. Rütting, Z. Jia and H.J. Laanbroek. 2017. Soil warming and fertilization altered rates of nitrogen transformation processes and selected for adapted ammonia-oxidizing archaea in sub-arctic grassland soil. Soil Biology and Biochemistry 107: 114-124.
68. Dan, X., Z. Chen, S. Dai, X. He, Z. Cai, J. Zhang, et al. 2019. Effects of Changing Temperature on Gross N Transformation Rates in Acidic Subtropical Forest Soils. Forests 10: 894.
69. Dannenmann, M., G. Willibald, S. Sippel and K. Butterbach-Bahl. 2011. Nitrogen dynamics at undisturbed and burned Mediterranean shrublands of Salento Peninsula, Southern Italy. Plant and Soil 343: 5-15.
70. Dannenmann, M., R. Gasche and H. Papen. 2007. Nitrogen turnover and N_2O production in the forest floor of beech stands as influenced by forest management. Journal of Plant Nutrition and Soil Science 170: 134-144.
71. Dannenmann, M., R. Gasche, A. Ledebuhr and H. Papen. 2006. Effects of forest management on soil N cycling in beech forests stocking on calcareous soils. Plant and Soil 287: 279-300.
72. Darby, I., C.-Y. Xu, H.M. Wallace, S. Joseph, B. Pace and S.H. Bai. 2016. Short-term dynamics of carbon and nitrogen using compost, compost-biochar mixture and organo-mineral biochar. Environmental Science and Pollution Research 23(11): 11267-11278.
73. Davidson, E. A., J. M. Stark and M. K. Firestone. 1990. Microbial production and consumption of nitrate in an annual grassland. Ecology 71(5): 1968-1975.
74. Davidson, E., P. Matson, P. Vitousek, R. Riley, K. Dunkin, G. Garcia-Mendez, et al. 1993. Processes Regulating Soil Emissions of NO and N_2O in a Seasonally Dry Tropical Forest. Ecology 74: 130-139.
75. Davidson, E.A., S.C. Hart and M.K. Firestone. 1992. Internal cycling of nitrate in soils of a mature coniferous forest. Ecology 73: 1148-1156.

76. Decock, C. and J. Six. 2013. An assessment of N-cycling and sources of N_2O during a simulated rain event using natural abundance ^{15}N. Agriculture, Ecosystems & Environment 165: 141-150.
77. DeLuca, T. and D. Keeney. 1995. Short-term transformation of applied $^{15}NO_3^-$ prairie and cultivated soils. Applied Soil Ecology 2(2): 131-135.
78. DeLuca, T., M. MacKenzie, M. Gundale and W. Holben. 2006. Wildfire-produced charcoal directly influences nitrogen cycling in ponderosa pine forests. Soil Science Society of America Journal 70(2): 448-453.
79. Demey, A., T. Rütting, D. Huygens, J. Staelens, M. Hermy, K. Verheyen, et al. 2014. Hemiparasitic litter additions alter gross nitrogen turnover in temperate semi-natural grassland soils. Soil Biology and Biochemistry 68: 419-428.
80. Deppe, M., R. Well, A. Giesemann, O. Spott and H. Flessa. 2017. Soil N_2O fluxes and related processes in laboratory incubations simulating ammonium fertilizer depots. Soil Biology and Biochemistry 104: 68-80.
81. DeVries, S.L., K.A. Block, M. Loving, L. Logozzo and P. Zhang. 2020. The effects of trace narasin on the biogeochemical N-cycle in a cultivated sandy loam. Science of The Total Environment 716: 137031.
82. Dong, W., C. Hu, Y. Zhang and D. Wu. 2012. Gross mineralization, nitrification and N_2O emission under different tillage in the North China Plain. Nutrient Cycling in Agroecosystems 94: 237-247.
83. Duan, M., J. House, Y. Liu and S.X. Chang. 2018. Contrasting responses of gross and net nitrogen transformations to salinity in a reclaimed boreal forest soil. Biology and Fertility of Soils 54: 385-395.
84. Fernández-Fernández, M., T. Rütting and S. González-Prieto. 2017. Effects of a high-severity wildfire and post-fire straw mulching on gross nitrogen dynamics in Mediterranean shrubland soil. Geoderma 305: 328-335.
85. Fierer, N., J.P. Schimel, R.G. Cates and J. Zou. 2001. Influence of balsam poplar tannin fractions on carbon and nitrogen dynamics in Alaskan taiga floodplain soils. Soil Biology and Biochemistry 33: 1827-1839.
86. Figueiredo, V., A. Enrich-Prast and T. Rütting. 2019. Evolution of nitrogen cycling in regrowing Amazonian rainforest. Scientific Reports 9: 8538.
87. Finzi, A.C. and W.H. Schlesinger. 2003. Soil–nitrogen cycling in a pine forest exposed to 5 years of elevated carbon dioxide. Ecosystems 6: 444-456.
88. Fisk, L., L. Barton, D. Jones, H. Glanville and D. Murphy. 2015. Root exudate carbon mitigates nitrogen loss in a semi-arid soil. Soil Biology and Biochemistry 88: 380-389.
89. Fisk, M.C. and T.J. Fahey. 2001. Microbial biomass and nitrogen cycling responses to fertilization and litter removal in young northern hardwood forests. Biogeochemistry 53: 201-223.
90. Fisk, M.C., D.R. Zak and T.R. Crow. 2002. Nitrogen storage and cycling in old-and second-growth northern hardwood forests. Ecology 83: 73-87.
91. Fisk, M.C., S.K. Schmidt and T.R. Seastedt. 1998. Topographic patterns of above-and belowground production and nitrogen cycling in alpine tundra. Ecology 79: 2253-2266.
92. Fitzhugh, R.D., G.M. Lovett and, R.T. Venterea. 2003. Biotic and abiotic immobilization of ammonium, nitrite, and nitrate in soils developed under different tree species in the Catskill Mountains, New York, USA. Global Change Biology 9(11): 1591-1601.

93. Flavel, T.C. and D.V. Murphy. 2006. Carbon and nitrogen mineralization rates after application of organic amendments to soil. Journal of Environmental Quality 35: 183-193.
94. Friedl, J., C. Scheer, D.W. Rowlings, E. Deltedesco, M. Gorfer, D. De Rosa, et al. 2020. Effect of the nitrification inhibitor 3, 4-dimethylpyrazole phosphate (DMPP) on N-turnover, the N_2O reductase-gene nosZ and N_2O: N_2 partitioning from agricultural soils. Scientific Reports 10: 1-11.
95. Friedl, J., D. De Rosa, D.W. Rowlings, P.R. Grace, C. Müller and, C. Scheer. 2018. Dissimilatory nitrate reduction to ammonium (DNRA), not denitrification dominates nitrate reduction in subtropical pasture soils upon rewetting. Soil Biology and Biochemistry 125: 340-349.
96. Fuchslueger, L., B. Wild, M. Mooshammer, M. Takriti, S. Kienzl, A. Knoltsch, et al. 2019. Microbial carbon and nitrogen cycling responses to drought and temperature in differently managed mountain grasslands. Soil Biology and Biochemistry 135: 144-153.
97. Fuchslueger, L., E.-M. Kastl, F. Bauer, S. Kienzl, R. Hasibeder, T. Ladreiter-Knauss, et al. 2014. Effects of drought on nitrogen turnover and abundances of ammonia-oxidizers in mountain grassland. Biogeosciences 11: 6003-6015.
98. Fungo, B., Z. Chen, K. Butterbach-Bahl, J. Lehmannn, G. Saiz, V. Braojos, et al. 2019. Nitrogen turnover and N_2O/N_2 ratio of three contrasting tropical soils amended with biochar. Geoderma 348: 12-20.
99. Gao, W., L. Kou, H. Yang, J. Zhang, C. Müller and S. Li. 2016b. Are nitrate production and retention processes in subtropical acidic forest soils responsive to ammonium deposition? Soil Biology and Biochemistry 100: 102-109.
100. Gao, W., L. Kou, J. Zhang, C. Müller, H. Wang, H. Yang, et al. 2016a. Enhanced deposition of nitrate alters microbial cycling of N in a subtropical forest soil. Biology and Fertility of Soils 52: 977-986.
101. Gao, W., L. Kou, J. Zhang, C. Müller, H. Yang and S. Li. 2016c. Ammonium fertilization causes a decoupling of ammonium cycling in a boreal forest. Soil Biology and Biochemistry 101: 114-123.
102. Garcia-Montiel, D. and D. Binkley. 1998. Effect of Eucalyptus saligna and Albizia falcataria on soil processes and nitrogen supply in Hawaii. Oecologia 113: 547-556.
103. Geisseler, D., R.G. Joergensen and B. Ludwig. 2012. Temporal effect of straw addition on amino acid utilization by soil microorganisms. European Journal of Soil Biology 53: 107-113.
104. Gerschlauer, F., M. Dannenmann, A. Kühnel, R. Meier, A. Kolar, K. Butterbach-Bahl, et al. 2016. Gross nitrogen turnover of natural and managed tropical ecosystems at Mt. Kilimanjaro, Tanzania. Ecosystems 19: 1271-1288.
105. Geßler, A., K. Jung, R. Gasche, H. Papen, A. Heidenfelder, E. Börner, et al. 2005. Climate and forest management influence nitrogen balance of European beech forests: microbial N transformations and inorganic N net uptake capacity of mycorrhizal roots. European Journal of Forest Research 124: 95-111.
106. Göbel, L.C. 2020. Nutrient Response Efficiencies, Leaching Losses and Soil-N Cycling in Temperate Grassland Agroforestry and Open Grassland Management Systems. PhD Thesis .
107. Gómez-Muñoz, B., J. Magid and L.S. Jensen. 2017. Nitrogen turnover, crop use efficiency and soil fertility in a long-term field experiment amended with different qualities of urban and agricultural waste. Agriculture, Ecosystems & Environment 240: 300-313.

108. Gómez-Rey, M., A. Couto-Vázquez and S. González-Prieto. 2012. Nitrogen transformation rates and nutrient availability under conventional plough and conservation tillage. Soil and Tillage Research 124: 144-152.
109. Gómez-Rey, M.X. and S.J. González-Prieto. 2015. Soil gross N transformation rates after a wildfire and straw mulch application for burned soil emergency stabilisation. Biology and Fertility of Soils 51: 493-505.
110. Gómez-Rey, M.X., M. Madeira, S.J. Gonzalez-Prieto and J. Coutinho. 2010. Soil C and N dynamics within a precipitation gradient in Mediterranean eucalypt plantations. Plant and Soil 336: 157-171.
111. Gómez-Rey, M.X., M. Madeira, S.J. Gonzalez-Prieto and J. Coutinho. 2013. Soil C and N dynamics in a Mediterranean oak woodland with shrub encroachment. Plant and Soil 371: 339-354.
112. Griffin, T. 2007. Estimates of gross transformation rates of dairy manure N using ^{15}N pool dilution. Communications in Soil Science and Plant Analysis 38: 1451-1465.
113. Groffman, P.M., M.C. Fisk, C.T. Driscoll, G.E. Likens, T.J. Fahey, C. Eagar, et al. 2006. Calcium additions and microbial nitrogen cycle processes in a northern hardwood forest. Ecosystems 9: 1289-1305.
114. Guckland, A., M.D. Corre and H. Flessa. 2010. Variability of soil N cycling and N_2O emission in a mixed deciduous forest with different abundance of beech. Plant and soil 336: 25-38.
115. Gundersen, P., B.A. Emmett, O.J. Kjønaas, C.J. Koopmans and A. Tietema. 1998. Impact of nitrogen deposition on nitrogen cycling in forests: a synthesis of NITREX data. Forest Ecology and Management 101: 37-55.
116. Gütlein, A., M. Dannenmann and R. Kiese. 2016. Gross nitrogen turnover rates of a tropical lower montane forest soil: Impacts of sample preparation and storage. Soil Biology and Biochemistry 95: 8-10.
117. Gütlein, A., M. Zistl-Schlingmann, J.N. Becker, N.S. Cornejo, F. Detsch, M. Dannenmann, et al. 2017. Nitrogen turnover and greenhouse gas emissions in a tropical alpine ecosystem, Mt. Kilimanjaro, Tanzania. Plant and Soil 411: 243-259.
118. Habteselassie, M.Y., J.M. Stark, B.E. Miller, S.G. Thacker and J.M. Norton. 2006. Gross nitrogen transformations in an agricultural soil after repeated dairy-waste application. Soil Science Society of America Journal 70: 1338-1348.
119. Hall, S.J. and P.A. Matson. 1999. Nitrogen oxide emissions after nitrogen additions in tropical forests. Nature 400: 152-155.
120. Hall, S.J. and P.A. Matson. 2003. Nutrient status of tropical rain forests influences soil N dynamics after N additions. Ecological Monographs 73: 107-129.
121. Hamer, U. and F. Makeschin. 2009. Rhizosphere soil microbial community structure and microbial activity in set-aside and intensively managed arable land. Plant and Soil 316: 57-69.
122. Hamer, U., F. Makeschin, J. Stadler and S. Klotz. 2008. Soil organic matter and microbial community structure in set-aside and intensively managed arable soils in NE-Saxony, Germany. Applied Soil Ecology 40: 465-475.
123. Hamer, U., F. Makeschin, S. An and F. Zheng. 2009. Microbial activity and community structure in degraded soils on the Loess Plateau of China. Journal of Plant Nutrition and Soil Science 172: 118-126.

124. Hamilton, R.L., M. Trimmer, C. Bradley and G. Pinay. 2016. Deforestation for oil palm alters the fundamental balance of the soil N cycle. Soil Biology and Biochemistry 95: 223-232.
125. Han, W.-Y., Xu, J.-M., Yi, X.-Y. and Lin, Y.-D., 2012. Net and gross nitrification in tea soils of varying productivity and their adjacent forest and vegetable soils. Soil Science and Plant Nutrition, 58(2): 173-182.
126. Han, X., W. Shen, J. Zhang and C. Müller. 2018. Microbial adaptation to long-term N supply prevents large responses in N dynamics and N losses of a subtropical forest. Science of the Total Environment 626: 1175-1187.
127. Hao, T., Y. Zhang, J. Zhang, C. Müller, K. Li, K. Zhang, et al. 2020. Chronic nitrogen addition differentially affects gross nitrogen transformations in alpine and temperate grassland soils. Soil Biology and Biochemistry 149: 107962.
128. Hart, S.C., D. Binkley and D.A. Perry. 1997. Influence of red alder on soil nitrogen transformations in two conifer forests of contrasting productivity. Soil Biology and Biochemistry 29: 1111-1123.
129. Hart, S.C., G. Nason, D.D. Myrold and D.A. Perry. 1994. Dynamics of gross nitrogen transformations in an old-growth forest: The carbon connection. Ecology 75: 880-891.
130. Hatch, D., R. Lovell, R. Antil, S. Jarvis and P. Owen. 2000. Nitrogen mineralization and microbial activity in permanent pastures amended with nitrogen fertilizer or dung. Biology and Fertility of Soils 30: 288-293.
131. Hatch, D.J., M.S. Sprosen, S.C. Jarvis and S.F. Ledgard. 2002. Use of labelled nitrogen to measure gross and net rates of mineralization and microbial activity in permanent pastures following fertilizer applications at different time intervals. Rapid Communications in Mass Spectrometry 16: 2172-2178.
132. He, Xiaoxiang; Chi, Qiaodong; Cai, Zucong; Cheng, Yi; Zhang, Jinbo; Müller, Christoph (2020). 15N tracing studies including plant N uptake processes provide new insights on gross N transformations in soil-plant systems. Soil Biology and Biochemistry, 141, 107666–.
133. Herrmann, A.M. and E. Coucheney. 2019. Temperature responses of nitrogen processes cannot be inferred from carbon turnover in a boreal Norway spruce forest. Journal of Plant Nutrition and Soil Science 182: 934-944.
134. Herrmann, A.M., E. Witter and T. Kätterer. 2007. Use of acetylene as a nitrification inhibitor to reduce biases in gross N transformation rates in a soil showing rapid disappearance of added ammonium. Soil Biology and Biochemistry 39: 2390-2400.
135. Hirobe, M., K. Koba and N. Tokuchi. 2003. Dynamics of the internal soil nitrogen cycles under moder and mull forest floor types on a slope in a Cryptomeria japonica D. Don plantation. Ecological Research 18: 53-64.
136. Hoeft, I., A. Keuter, C.M. Quiñones, P. Schmidt-Walter, E. Veldkamp and M.D. Corre 2014. Nitrogen retention efficiency and nitrogen losses of a managed and phytodiverse temperate grassland. Basic and Applied Ecology 15(3): 207-218.
137. Holmes, W.E. and D.R. Zak. 1999. Soil microbial control of nitrogen loss following clear-cut harvest in northern hardwood ecosystems. Ecological Applications 9: 202-215.
138. Holmes, W.E., D.R. Zak, K.S. Pregitzer and J.S. King. 2003. Soil nitrogen transformations under Populus tremuloides, Betula papyrifera and Acer saccharum following 3 years exposure to elevated CO_2 and O_3. Global Change Biology 9: 1743-1750.

139. Holub, S.M., K. Lajtha, J.D. Spears, J.A. Tóth, S.E. Crow, B.A. Caldwell, et al. 2005. Organic matter manipulations have little effect on gross and net nitrogen transformations in two temperate forest mineral soils in the USA and central Europe. Forest Ecology and Management 214: 320-330.
140. Holz, M., M. Aurangojeb, Å. Kasimir, P. Boeckx, Y. Kuzyakov, L. Klemedtsson, et al. 2016. Gross nitrogen dynamics in the mycorrhizosphere of an organic forest soil. Ecosystems 19: 284-295.
141. Hooker, T.D. and J.M. Stark. 2008. Soil C and N cycling in three semiarid vegetation types: response to an in situ pulse of plant detritus. Soil Biology and Biochemistry 40: 2678-2685.
142. Hoyle, F. and D. Murphy. 2006. Seasonal changes in microbial function and diversity associated with stubble retention versus burning. Soil Research 44: 407-423.
143. Hoyle, F., D. Murphy and I. Fillery. 2006. Temperature and stubble management influence microbial CO_2–C evolution and gross N transformation rates. Soil Biology and Biochemistry 38: 71-80.
144. Hu, X., C. Liu, X. Zheng, M. Dannenmann, K. Butterbach-Bahl, Z. Yao, W. Zhang, R. Wang and G. Cao. 2019. Annual dynamics of soil gross nitrogen turnover and nitrous oxide emissions in an alpine shrub meadow. Soil Biology and Biochemistry 138: 107576.
145. Hu, Y.-L., F.-P. Wu, D.-H. Zeng and S.X. Chang. 2014. Wheat straw and its biochar had contrasting effects on soil C and N cycling two growing seasons after addition to a Black Chernozemic soil planted to barley. Biology and Fertility of Soils 50: 1291-1299.
146. Huang, X., X. Zhu-Barker, W.R. Horwath, S.J. Faeflen, H. Luo, X. Xin, et al. 2016. Effect of iron oxide on nitrification in two agricultural soils with different pH. Biogeosciences 13: 5609-5617.
147. Huang, Z., Z. Xu and C. Chen. 2008. Effect of mulching on labile soil organic matter pools, microbial community functional diversity and nitrogen transformations in two hardwood plantations of subtropical Australia. Applied Soil Ecology 40: 229-239.
148. Hungate, B.A., P. Dijkstra, D.W. Johnson, C.R. Hinkle and B.G. Drake. 1999. Elevated CO_2 increases nitrogen fixation and decreases soil nitrogen mineralization in Florida scrub oak. Global Change Biology 5: 781-789.
149. Huygens, D., J. Schouppe, D. Roobroeck, M. Alvarez, O. Balocchi, E. Valenzuela, D. Pinochet and P. Boeckx. 2011. Drying–rewetting effects on N cycling in grassland soils of varying microbial community composition and management intensity in south central Chile. Applied Soil Ecology, 48(3): 270-279.
150. Huygens, D., T. Rütting, P. Boeckx, O. Van Cleemput, R. Godoy and C. Müller. 2007. Soil nitrogen conservation mechanisms in a pristine south Chilean Nothofagus forest ecosystem. Soil Biology and Biochemistry 39: 2448-2458.
151. Huygens, D.; P. Boeckx, P. Templer, L. Paulino O. Van Cleemput, C. Oyarzún, C. Müller, R. Godoy. 2008. Mechanisms for retention of bioavailable nitrogen in volcanic rainforest soils. Nature Geoscience 1(8): 543–548.
152. Inselsbacher, E., N.H.-N. Umana, F.C. Stange, M. Gorfer, E. Schüller, K. Ripka, et al. 2010. Short-term competition between crop plants and soil microbes for inorganic N fertilizer. Soil Biology and Biochemistry 42: 360-372.
153. Inselsbacher, E., K. Ripka, S. Klaubauf, D. Fedosoyenko, E. Hackl, M. Gorfer, et al. 2009. A cost-effective high-throughput microcosm system for studying nitrogen dynamics at the plant-microbe-soil interface. Plant and Soil 317(1-2): 293-307.

154. Inselsbacher, E., W. Wanek, J. Strauss, S. Zechmeister-Boltenstern and C. Müller. 2013. A novel ^{15}N tracer model reveals: plant nitrate uptake governs nitrogen transformation rates in agricultural soils. Soil Biology and Biochemistry 57: 301-310.
155. Isobe, K., H. Oka, T. Watanabe, R. Tateno, R. Urakawa, C. Liang, et al. 2018. High soil microbial activity in the winter season enhances nitrogen cycling in a cool-temperate deciduous forest. Soil Biology and Biochemistry 124: 90-100.
156. Isobe, K., Ohte, N., Oda, T., Murabayashi, S., Wei, W., Senoo, K., Tokuchi, N. and Tateno, R., 2015. Microbial regulation of nitrogen dynamics along the hillslope of a natural forest. Frontiers in Environmental Science 2: 63.
157. Iversen, C.M., T. Hooker, A.T. Classen and R.J. Norby. 2011. Net mineralization of N at deeper soil depths as a potential mechanism for sustained forest production under elevated [CO_2]. Global Change Biology 17: 1130-1139.
158. Jahangir, M., O. Fenton, R. Carolan, R. Harrington, P. Johnston, M. Zaman, et al. 2020. Application of ^{15}N tracing for estimating nitrogen cycle processes in soils of a constructed wetland. Water Research 183: 116062.
159. Jamieson, N., D. Barraclough, M. Unkovich and R. Monaghan. 1998. Soil N dynamics in a natural calcareous grassland under a changing climate. Biology and Fertility of Soils 27: 267-273.
160. Jiang, X., X. Xin, S. Li, J. Zhou, T. Zhu, C. Müller, et al. 2015. Effects of Fe oxide on N transformations in subtropical acid soils. Scientific Reports 5: 8615.
161. Jin, X., J. Huang and Y. Zhou. 2012. Impact of coastal wetland cultivation on microbial biomass, ammonia-oxidizing bacteria, gross N transformation and N_2O and NO potential production. Biology and Fertility of Soils 48: 363-369.
162. Joy S. Clein; Joshua P. Schimel (1995). Nitrogen turnover and availability during succession from alder to poplar in Alaskan taiga forests. Soil Biology and Biochemistry, 27(6), 0–752.
163. Kaiser, C., H. Meyer, C. Biasi, O. Rusalimova, P. Barsukov and A. Richter. 2005. Storage and mineralization of carbon and nitrogen in soils of a frost-boil tundra ecosystem in Siberia. Applied Soil Ecology 29: 173-183.
164. Kaiser, C., H. Meyer, C. Biasi, O. Rusalimova, P. Barsukov and A. Richter. 2007. Conservation of soil organic matter through cryoturbation in arctic soils in Siberia. Journal of Geophysical Research: Biogeosciences 112.
165. Kaiser, C., L. Fuchslueger, M. Koranda, M. Gorfer, C.F. Stange, B. Kitzler, et al. 2011. Plants control the seasonal dynamics of microbial N cycling in a beech forest soil by belowground C allocation. Ecology 92: 1036-1051.
166. Kaye, J.P. and S.C. Hart. 1998. Ecological restoration alters nitrogen transformations in a ponderosa pine–bunchgrass ecosystem. Ecological Applications 8: 1052-1060.
167. Kellman, L., S. Kumar and A. Diochon. 2014. Soil nitrogen dynamics within profiles of a managed moist temperate forest chronosequence consistent with long-term harvesting-induced losses. Journal of Geophysical Research: Biogeosciences 119: 1309-1321.
168. Kiese, R., B.Hewett and K. Butterbach-Bahl. 2008. Seasonal dynamic of gross nitrification and N_2O emission at two tropical rainforest sites in Queensland, Australia. Plant and Soil 309(1-2): 105-117.
169. Koranda, M., J. Schnecker, C. Kaiser, L. Fuchslueger, B. Kitzler, C.F. Stange, et al. 2011. Microbial processes and community composition in the rhizosphere of European beech–the influence of plant C exudates. Soil Biology and Biochemistry 43: 551-558.

170. Koranda, M., C. Kaiser, L. Fuchslueger, B. Kitzler, A. Sessitsch, S. Zechmeister-Boltenstern and A. Richter. 2013. Seasonal variation in functional properties of microbial communities in beech forest soil. Soil Biology and Biochemistry 60: 95-104.
171. Koyama, A., K.L. Kavanagh and K. Stephan. 2010. Wildfire effects on soil gross nitrogen transformation rates in coniferous forests of central Idaho, USA. Ecosystems 13: 1112-1126.
172. Koyama, A., K. Stephan and K.L. Kavanagh. 2012. Fire effects on gross inorganic N transformation in riparian soils in coniferous forests of central Idaho, USA: wildfires v. prescribed fires. International Journal of Wildland Fire 21(1): 69-78.
173. Kuroiwa, M., K. Koba, K. Isobe, R. Tateno, A. Nakanishi, Y. Inagaki, et al. 2011. Gross nitrification rates in four Japanese forest soils: heterotrophic versus autotrophic and the regulation factors for the nitrification. Journal of Forest Research 16: 363.
174. Kwak, J.-H., M.A. Naeth and S.X. Chang. 2018. Microbial Activities and Gross Nitrogen Transformation Unaffected by Ten-Year Nitrogen and Sulfur Addition. Soil Science Society of America Journal 82: 362-370.
175. Lai, T.V., R. Farquharson and M.D. Denton. 2019. High soil temperatures alter the rates of nitrification, denitrification and associated N_2O emissions. Journal of Soils and Sediments 19(5): 2176-2189.
176. Laine, M., T. Rütting, L. Alakukku, A. Palojärvi and R. Strömmer. 2018. Process rates of nitrogen cycle in uppermost topsoil after harvesting in no-tilled and ploughed agricultural clay soil. Nutrient Cycling in Agroecosystems 110: 39-49.
177. Lama, S., T. Kuhn, M.F. Lehmann C. Müller, O. Gonzalez N. Eisenhauer, et al. 2020. The biodiversity-N cycle relationship: a ^{15}N tracer experiment with soil from plant mixtures of varying diversity to model N pool sizes and transformation rates. Biology and Fertility of Soils 56(7): 1047-1061.
178. Lan, T., R. Liu, H. Suter, O. Deng, X. Gao, L. Luo, S. Yuan, C. Wang and D. Chen. 2020. Stimulation of heterotrophic nitrification and N_2O production, inhibition of autotrophic nitrification in soil by adding readily degradable carbon. Journal of Soils and Sediments 20(1): 81-90.
179. Lan, T., Y. Han, M. Roelcke, R. Nieder and Z. Cai. 2014. Temperature dependence of gross N transformation rates in two Chinese paddy soils under aerobic condition. Biology and Fertility of Soils 50: 949-959.
180. Landi, L., D. Barraclough, L. Badalucco, A. Gelsomino and P. Nannipieri. 1999. l-Methionine-sulphoximine affects N mineralisation-immobilisation in soil. Soil Biology and Biochemistry 31: 253-259.
181. Landi, L., F. Valori, J. Ascher, G. Renella, L. Falchini and P. Nannipieri. 2006. Root exudate effects on the bacterial communities, CO_2 evolution, nitrogen transformations and ATP content of rhizosphere and bulk soils. Soil Biology and Biochemistry 38: 509-516.
182. Lang, M.; P. Li, X. Han, Y. Qiao, S. Miao. 2016. Gross nitrogen transformations in black soil under different land uses and management systems. Biology and Fertility of Soils 52(2): 233–241.
183. Lang, M., P. Li, C.Ti, S. Zhu, X. Yan and S.X. Chang. 2019. Soil gross nitrogen transformations are related to land-uses in two agroforestry systems. Ecological Engineering 127: 431–439.
184. Larsen, K.S., L.C. Andresen, C. Beier, S. Jonasson, K.R. Albert, P. Ambus, et al. 2011. Reduced N cycling in response to elevated CO_2, warming, and drought in a Danish

heathland: synthesizing results of the CLIMAITE project after two years of treatments. Global Change Biology 17: 1884-1899.
185. Laughlin, R., T. Rütting, C. Müller, C. Watson and R. Stevens. 2009. Effect of acetate on soil respiration, N_2O emissions and gross N transformations related to fungi and bacteria in a grassland soil. Applied Soil Ecology 42: 25-30.
186. Laungani, R. and J.M. Knops. 2012. Microbial immobilization drives nitrogen cycling differences among plant species. Oikos 121: 1840-1848.
187. LeDuc, S.D. and D.E. Rothstein. 2007. Initial recovery of soil carbon and nitrogen pools and dynamics following disturbance in jack pine forests: a comparison of wildfire and clearcut harvesting. Soil Biology and Biochemistry 39: 2865-2876.
188. Lewis, D.B. and J.P. Kaye. 2012. Inorganic nitrogen immobilization in live and sterile soil of old-growth conifer and hardwood forests: implications for ecosystem nitrogen retention. Biogeochemistry 111(1-3): 169-186.
189. Li, D., J. Liu, H. Chen, L. Zheng and K. Wang. 2018. Soil gross nitrogen transformations in responses to land use conversion in a subtropical karst region. Journal of Environmental Management 212: 1-7.
190. Li, D., Y. Yang, H. Chen, K. Xiao, T. Song and K. Wang. 2017. Soil gross nitrogen transformations in typical karst and nonkarst forests, southwest China. Journal of Geophysical Research: Biogeosciences 122: 2831-2840.
191. Li, F., X. Liang, S. He, M. Li, Y. Cao, J. Zhang and G. Tian. 2020. Biochar slows gross nitrification and gasses N emission via lower autotrophic nitrification in paddy soils. Journal of Soils and Sediments 20(2): 629-640.
192. Li, G., F. Meng, P. Zhu, C. Lu, W. Yu and C. Müller. 2018. Fertilizer type and organic amendments affect gross N dynamics in a Chinese Chernozem. European Journal of Soil Science 69: 1117-1125.
193. Li, P. and M. Lang. 2014. Gross nitrogen transformations and related N_2O emissions in uncultivated and cultivated black soil. Biology and Fertility of Soils 50: 197-206.
194. Li, Z., S. Xia, R. Zhang, R. Zhang, F. Chen and Y. Liu. 2020. N_2O emissions and product ratios of nitrification and denitrification are altered by K fertilizer in acidic agricultural soils. Environmental Pollution 265: 115065.
195. Liimatainen, M., C. Voigt, P.J. Martikainen, J. Hytönen, K. Regina, H. Oskarsson, et al. 2018. Factors controlling nitrous oxide emissions from managed northern peat soils with low carbon to nitrogen ratio. Soil Biology and Biochemistry 122: 186-195.
196. Liu, H., Y. Ding, Q. Zhang, X. Liu, J. Xu, Y. Li and H. Di. 2019. Heterotrophic nitrification and denitrification are the main sources of nitrous oxide in two paddy soils. Plant and Soil 445(1): 39-53.
197. Liu, R., H. Suter, J. He, H. Hayden and D. Chen. 2015. Influence of temperature and moisture on the relative contributions of heterotrophic and autotrophic nitrification to gross nitrification in an acid cropping soil. Journal of Soils and Sediments 15(11): 2304-2309.
198. Liu, S., Q. Chi, J. Shan, B. Zhu, X. Zhang, Y. Cheng, et al. 2020. Evaluation of the effectiveness of N process inhibitors in paddy rice via ^{15}N tracing approach. Soil Biology and Biochemistry 147:107855.
199. Liu, S., Q. Chi, Y. Cheng, B. Zhu, W. Li, X. Zhang, et al. 2019. Importance of matching soil N transformations, crop N form preference, and climate to enhance crop yield and reducing N loss. Science of The Total Environment 657: 1265-1273.

200. Liu, S., X. Zhang, A. Liang, J. Zhang, C. Müller and Z. Cai. 2018. Ridge tillage is likely better than no tillage for 14-year field experiment in black soils: Insights from a ^{15}N-tracing study. Soil and Tillage Research 179: 38-46.
201. Liu, S., X. Zhang, J. Zhao, J. Zhang, C. Müller and Z. Cai. 2017. Effects of long-term no tillage treatment on gross soil N transformations in black soil in Northeast China. Geoderma 301: 42-46.
202. Liu, X., C. Guo, S. He, H. Zhu, J. Li, Z. Yu, et al. 2020. Divergent gross nitrogen transformation paths in the topsoil and subsoil between abandoned and agricultural cultivation land in irrigated areas. Science of The Total Environment 716: 137148.
203. Lteif, A., J.K. Whalen, R.L. Bradley and C. Camiré. 2010. Nitrogen transformations revealed by isotope dilution in an organically fertilized hybrid poplar plantation. Plant and Soil 333: 105-116.
204. Luxhøi, J., I. Fillery, D. Murphy, S. Bruun, L.S. Jensen and S. Recous. 2008a. Distribution and controls on gross N mineralization-immobilization-turnover in soil subjected to zero tillage. European Journal of Soil Science 59: 190-197.
205. Luxhøi, J., I. Fillery, S. Recous and L.S. Jensen. 2008b. Carbon and N turnover in moist sandy soil following short exposure to a range of high soil temperature regimes. Soil Research 46: 710-718.
206. Luxhøi, J., N. Nielsen and L. Jensen. 2003. Influence of $^{15}NH_4^+$-application on gross N turnover rates in soil. Soil Biology and Biochemistry 35: 603-606.
207. Luxhøi, J., S. Bruun, B. Stenberg, T.A. Breland and L.S. Jensen. 2006. Prediction of gross and net nitrogen mineralization-immobilization-turnover from respiration. Soil Science Society of America Journal 70: 1121-1128.
208. Ma, Q., Y. Wen, D. Wang, X. Sun, P.W. Hill, A. Macdonald, et al. 2020. Farmyard manure applications stimulate soil carbon and nitrogen cycling by boosting microbial biomass rather than changing its community composition. Soil Biology and Biochemistry: 107760.
209. Mack, M.C. and C.M. D'Antonio. 2003. Exotic grasses alter controls over soil nitrogen dynamics in a Hawaiian woodland. Ecological Applications 13: 154-166.
210. Maestrini, B., A.M. Herrmann, P. Nannipieri, M.W. Schmidt and S. Abiven. 2014. Ryegrass-derived pyrogenic organic matter changes organic carbon and nitrogen mineralization in a temperate forest soil. Soil Biology and Biochemistry 69: 291-301.
211. Mao, C., D. Kou, G. Wang, Y. Peng, G. Yang, F. Liu, et al. 2019. Trajectory of topsoil nitrogen transformations along a thermo-erosion gully on the Tibetan Plateau. Journal of Geophysical Research: Biogeosciences 124: 1342-1354.
212. Masse, J., C.E. Prescott, C. Müller and S.J. Grayston. 2016. Gross nitrogen transformation rates differ in reconstructed oil-sand soils from natural boreal-forest soils as revealed using a 15N tracing method. Geoderma 282: 37-48.
213. Matejek, B., C. Huber, M. Dannenmann, M. Kohlpaintner, R. Gasche and H. Papen. 2010a. Microbial N turnover processes in three forest soil layers following clear cutting of an N saturated mature spruce stand. Plant and Soil 337: 93-110.
214. Matejek, B., C. Huber, M. Dannenmann, M. Kohlpaintner, R. Gasche, A. Göttlein, et al. 2010b. Microbial nitrogen-turnover processes within the soil profile of a nitrogen-saturated spruce forest and their relation to the small-scale pattern of seepage-water nitrate. Journal of Plant Nutrition and Soil Science 173: 224-236.

215. Mathieu, O., C. Hénault, J. Lévêque, E. Baujard, M.-J. Milloux and F. Andreux. 2006. Quantifying the contribution of nitrification and denitrification to the nitrous oxide flux using ^{15}N tracers. Environmental Pollution 144: 933-940.
216. Matson, A., D. Pennock and A. Bedard-Haughn. 2009. Methane and nitrous oxide emissions from mature forest stands in the boreal forest, Saskatchewan, Canada. Forest Ecology and Management 258: 1073-1083.
217. Matson, A.L., M.D. Corre and E. Veldkamp. 2014. Nitrogen cycling in canopy soils of tropical montane forests responds rapidly to indirect N and P fertilization. Global Change Biology 20(12): 3802-3813.
218. McGeough, K., C. Watson, C. Müller, R. Laughlin and D. Chadwick. 2016. Evidence that the efficacy of the nitrification inhibitor dicyandiamide (DCD) is affected by soil properties in UK soils. Soil Biology and Biochemistry 94: 222-232.
219. McKinley, D.C., C.W. Rice and J.M. Blair. 2008. Conversion of grassland to coniferous woodland has limited effects on soil nitrogen cycle processes. Soil Biology and Biochemistry 40: 2627-2633.
220. McMillan, R., S. Quideau, M. MacKenzie and O. Biryukova. 2007. Nitrogen mineralization and microbial activity in oil sands reclaimed boreal forest soils. Journal of Environmental Quality 36: 1470-1478.
221. Merilä, P., A. Smolander and R. Strömmer. 2002. Soil nitrogen transformations along a primary succession transect on the land-uplift coast in western Finland. Soil Biology and Biochemistry 34(3): 373-385.
222. Meyer, H., C.Kaiser, C. Biasi, R. Hämmerle, O. Rusalimova, N. Lashchinsky, et al. 2006. Soil carbon and nitrogen dynamics along a latitudinal transect in Western Siberia, Russia. Biogeochemistry 81(2): 239-252.
223. Miller, A. E., J.P. Schimel, J.O., Sickman, K. Skeen, T. Meixner and J.M. Melack. 2009. Seasonal variation in nitrogen uptake and turnover in two high-elevation soils: mineralization responses are site-dependent. Biogeochemistry 93(3): 253–270.
224. Minick, K., C. Pandey, T. Fox and S. Subedi. 2016. Dissimilatory nitrate reduction to ammonium and N_2O flux: effect of soil redox potential and N fertilization in loblolly pine forests. Biology and Fertility of Soils 52: 601-614.
225. Minick, K.J., B.D. Strahm, T.R. Fox, E.B. Sucre and Z.H. Leggett. 2015. Microbial nitrogen cycling response to forest-based bioenergy production. Ecological Applications 25: 2366-2381.
226. Mishra, S., H. Di, K. Cameron, R. Monaghan and A. Carran. 2005. Gross nitrogen mineralisation rates in pastural soils and their relationships with organic nitrogen fractions, microbial biomass and protease activity under glasshouse conditions. Biology and Fertility of Soils 42: 45-53.
227. Morse, J.L. and E.S. Bernhardt 2013. Using ^{15}N tracers to estimate N_2O and N_2 emissions from nitrification and denitrification in coastal plain wetlands under contrasting land-uses. Soil Biology and Biochemistry 57: 635-643.
228. Müller, C., R.J. Laughlin, O. Spott and T. Rütting. 2014. Quantification of N_2O emission pathways via a ^{15}N tracing model. Soil Biology and Biochemistry 72: 44-54.
229. Müller, C., R.J. Laughlin, P. Christie and C.J. Watson. 2011. Effects of repeated fertilizer and cattle slurry applications over 38 years on N dynamics in a temperate grassland soil. Soil Biology and Biochemistry 43: 1362-1371.

230. Münchmeyer, U., R. Russow and J. Augustin. 2000. Net and gross nitrogen mineralization in drained and reflooded fen soils. Isotopes in Environmental and Health Studies 36: 79-98.
231. Murphy, C.J., E.M. Baggs, N. Morley, D.P. Wall and E. Paterson. 2015. Rhizosphere priming can promote mobilisation of N-rich compounds from soil organic matter. Soil Biology and Biochemistry 81: 236–243.
232. Murphy, D., A. Bhogal, M. Shepherd, K. Goulding, S. Jarvis, D. Barraclough, et al. 1999. Comparison of ^{15}N labelling methods to measure gross nitrogen mineralisation. Soil Biology and Biochemistry 31: 2015-2024.
233. Murphy, D., G. Sparling and I. Fillery. 1998. Seasonal fluctuations in gross N mineralisation, ammonium consumption, and microbial biomass ina Western Australian soil under different land uses. Australian Journal of Agricultural Research 49: 523-536.
234. Muruganandam, S., D.W. Israel and W.P. Robarge. 2010. Nitrogen transformations and microbial communities in soil aggregates from three tillage systems. Soil Science Society of America Journal 74: 120-129.
235. Myrold, D.D. and N.R. Posavatz 2007. Potential importance of bacteria and fungi in nitrate assimilation in soil. Soil Biology and Biochemistry 39(7): 1737-1743.
236. Neill, C., M.C. Piccolo, J.M. Melillo, P.A. Steudler and C.C. Cerri. 1999. Nitrogen dynamics in Amazon forest and pasture soils measured by ^{15}N pool dilution. Soil Biology and Biochemistry 31: 567-572.
237. Nelissen, V., T. Rütting, D. Huygens, G. Ruysschaert and P. Boeckx. 2015. Temporal evolution of biochar's impact on soil nitrogen processes–a ^{15}N tracing study. GCB Bioenergy 7: 635-645.
238. Nelissen, V., T. Rütting, D. Huygens, J. Staelens, G. Ruysschaert and P. Boeckx. 2012. Maize biochars accelerate short-term soil nitrogen dynamics in a loamy sand soil. Soil Biology and Biochemistry 55: 20-27.
239. Niboyet, A., L. Barthes, B.A. Hungate, X. Le Roux, J.M. Bloor, A. Ambroise, et al. 2010. Responses of soil nitrogen cycling to the interactive effects of elevated CO_2 and inorganic N supply. Plant and Soil 327: 35-47.
240. Nira, R. 2003. Microbial, physical, and chemical factors causing higher gross rate and lower net rate of nitrogen mineralization in volcanic ash soils than in alluvial soils in the Tokachi District. Soil Science and Plant Nutrition 49: 417-423.
241. Nira, R. and T. Hashimoto. 1997. Depressed gross mineralization and immobilization of nitrogen and changes in microbial population in soil after heat treatment. Soil Science and Plant Nutrition 43: 451-455.
242. Nira, R., T. Hashimoto, M. Matsuzaki and A. Nishimune. 1996. Nitrogen transformations and availability in soils with application of fumigants. Soil Science and Plant Nutrition 42: 261-268.
243. Nishio, T. 1994. Estimating nitrogen transformation rates in surface aerobic soil of a paddy field. Soil Biology and Biochemistry 26(9): 1273-1280.
244. Nishio, T., T. Kanamori and T. Fujimoto. 1985. Nitrogen transformations in an aerobic soil as determined by a $^{15}NH_4^+$ dilution technique. Soil Biology and Biochemistry 17: 149-154.
245. Norton, J.M. and M.K. Firestone. 1996. N dynamics in the rhizosphere of Pinus ponderosa seedlings. Soil Biology and Biochemistry 28: 351-362.

246. Pantelopoulos, A., J. Magid and L.S. Jensen. 2016. Net and gross nitrogen turnover in soil amended with acidified and differently dried solids from biogas digestate. Soil Science Society of America Journal 80: 943-953.
247. Parfitt, R., N. Scott, D. Ross, G. Salt and K. Tate. 2003. Land-use change effects on soil C and N transformations in soils of high N status: comparisons under indigenous forest, pasture and pine plantation. Biogeochemistry 66: 203-221.
248. Perakis, S.S. and L.O. Hedin. 2001. Fluxes and fates of nitrogen in soil of an unpolluted old-growth temperate forest, southern Chile. Ecology 82: 2245-2260.
249. Piper, C.L., E.G. Lamb and S.D. Siciliano. 2015. Smooth brome changes gross soil nitrogen cycling processes during invasion of a rough fescue grassland. Plant Ecology 216: 235-246.
250. Portier, E., W.L. Silver and W.H. Yang. 2019. Invasive perennial forb effects on gross soil nitrogen cycling and nitrous oxide fluxes depend on phenology. Ecology 100: e02716.
251. Potthast, K., U. Hamer and F. Makeschin. 2012a. Land-use change in a tropical mountain rainforest region of southern Ecuador affects soil microorganisms and nutrient cycling. Biogeochemistry 111: 151-167.
252. Potthast, K., U. Hamer and F. Makeschin. 2012b. In an Ecuadorian pasture soil the growth of Setaria sphacelata, but not of soil microorganisms, is co-limited by N and P. Applied Soil Ecology 62: 103-114.
253. Pröll, G., S. Dullinger, T. Dirnböck, C. Kaiser, A. Richter. 2011. Effects of nitrogen on tree recruitment in a temperate montane forest as analysed by measured variables and Ellenberg indicator values. Preslia 83:111–127.
254. Prommer, J., W. Wanek, F. Hofhansl, D. Trojan, P. Offre, T. Urich, et al. 2014. Biochar decelerates soil organic nitrogen cycling but stimulates soil nitrification in a temperate arable field trial. PloS One 9: e86388.
255. Puri, G. and M. Ashman. 1998. Relationship between soil microbial biomass and gross N mineralisation. Soil Biology and Biochemistry 30: 251-256.
256. Rath, K.M., A. Maheshwari, P. Bengtson and J. Rousk. 2016. Comparative toxicities of salts on microbial processes in soil. Applied and Environmental Microbiology 82: 2012-2020.
257. Recous, S., C. Aita and B. Mary. 1998. In situ changes in gross N transformations in bare soil after addition of straw. Soil Biology and Biochemistry 31: 119-133.
258. Regehr, A., M. Oelbermann, C. Videla and L. Echarte. 2015. Gross nitrogen mineralization and immobilization in temperate maize-soybean intercrops. Plant and Soil 391: 353-365.
259. Remy, E., K. Wuyts, K. Verheyen, P. Gundersen and P. Boeckx. 2018. Altered microbial communities and nitrogen availability in temperate forest edges. Soil Biology and Biochemistry 116: 179-188.
260. Ribbons, R.R., D.J. Levy-Booth, J. Masse, S.J. Grayston, M.A. McDonald, L. Vesterdal, et al. 2016. Linking microbial communities, functional genes and nitrogen-cycling processes in forest floors under four tree species. Soil Biology and Biochemistry 103: 181-191.
261. Rosenkranz, P., M. Dannenmann, N. Brüggemann, H. Papen, U. Berger, E. Zumbusch, et al. 2010. Gross rates of ammonification and nitrification at a nitrogen-saturated spruce (Picea abies (L.) Karst.) stand in southern Germany. European Journal of Soil Science 61: 745-758.

262. Ross, D.J., N.A. Scott, K.R. Tate, N.J. Rodda and J.A. Townsend. 2001. Root effects on soil carbon and nitrogen cycling in a Pinus radiata D. Don plantation on a coastal sand. Soil Research 39: 1027-1039.
263. Ross, D.S., G.B. Lawrence and G. Fredriksen. 2004. Mineralization and nitrification patterns at eight northeastern USA forested research sites. Forest Ecology and Management 188: 317-335.
264. Rousk, K., J. Rousk, D.L. Jones, O. Zackrisson and T.H. DeLuca. 2013. Feather moss nitrogen acquisition across natural fertility gradients in boreal forests. Soil Biology and Biochemistry 61: 86-95.
265. Rütting, T. and C. Müller. 2008. Process-specific analysis of nitrite dynamics in a permanent grassland soil by using a Monte Carlo sampling technique. European Journal of Soil Science 59: 208-215.
266. Rütting, T. and M.J. Hovenden. 2020. Soil nitrogen cycle unresponsive to decadal long climate change in a Tasmanian grassland. Biogeochemistry 147: 99-107.
267. Rütting, T., T.J. Clough, C. Mueller, M. Lieffering and P.C. Newton. 2010. Ten years of elevated atmospheric carbon dioxide alters soil nitrogen transformations in a sheep-grazed pasture. Global Change Biology 16: 2530-2542.
268. Schaeffer, S.M., S.E. Ziegler, J. Belnap, R.D. Evans. 2012. Effects ofBromus tectoruminvasion on microbial carbon and nitrogen cycling in two adjacent undisturbed arid grassland communities. Biogeochemistry 111(1-3), 427–441.
269. Schimel, D.S. 1986. Carbon and nitrogen turnover in adjacent grassland and cropland ecosystems. Biogeochemistry 2: 345-357.
270. Scott, N.A., R.L. Parfitt, D.J. Ross and G.J. Salt. 1998. Carbon and nitrogen transformations in New Zealand plantation forest soils from sites with different N status. Canadian Journal of Forest Research 28: 967-976.
271. Sharma, N. and S. Kumar. 2020a. Gross nitrogen transformation rates in semiarid tropical soils under different salinity and vegetation conditions. Ecosphere 11: e03034.
272. Sharma, N. and S. Kumar. 2020b. Gross rates of nitrogen transformation in soils of a global biodiversity hotspot (Western Ghats, India). Journal of Plant Nutrition and Soil Science 183: 579-591.
273. Shaw, M.R. and J. Harte. 2001. Response of nitrogen cycling to simulated climate change: differential responses along a subalpine ecotone. Global Change Biology 7: 193-210.
274. Shi, X., H.-W. Hu, C. Müller, J.-Z. He, D. Chen and H.C. Suter. 2016. Effects of the nitrification inhibitor 3, 4-dimethylpyrazole phosphate on nitrification and nitrifiers in two contrasting agricultural soils. Applied and Environmental Microbiology 82: 5236-5248.
275. Shi, X., J. Wang, C. Müller, H.-W. Hu, J.-Z. He, J. Wang, et al. 2020. Dissimilatory nitrate reduction to ammonium dominates soil nitrate retention capacity in subtropical forests. Biology and Fertility of Soils 56(6): 785-797.
276. Shindo, H. and T. Nishio. 2005. Immobilization and remineralization of N following addition of wheat straw into soil: determination of gross N transformation rates by ^{15}N-ammonium isotope dilution technique. Soil Biology and Biochemistry 37: 425-432.
277. Silver, W.L., A. Thompson, A. Reich, J.J. Ewel and M. Firestone. 2005. Nitrogen cycling in tropical plantation forests: potential controls on nitrogen retention. Ecological Applications 15: 1604-1614.
278. Silver, W.L., D.J. Herman and M.K. Firestone. 2001. Dissimilatory nitrate reduction to ammonium in upland tropical forest soils. Ecology 82: 2410-2416.

279. Smart, D.R., J.M. Stark and V. Diego. 1999. Resource limitations to nitric oxide emissions from a sagebrush-steppe ecosystem. Biogeochemistry 47: 63-86.
280. Smithwick, E.A., M.G. Turner, K.L. Metzger and T.C. Balser. 2005. Variation in NH_4^+ mineralization and microbial communities with stand age in lodgepole pine (*Pinus contorta*) forests, Yellowstone National Park (USA). Soil Biology and Biochemistry 37: 1546-1559.
281. Smithwick, E.A., K.J. Naithani, T.C. Balser, W.H. Romme and M.G. Turner. 2012. Post-fire spatial patterns of soil nitrogen mineralization and microbial abundance. PloS One 7(11): e50597.
282. Song, L., J. Zhang, C. Müller and G. Jin. 2019a. Responses of soil N transformations and N loss to three years of simulated N deposition in a temperate Korean pine plantation in northeast China. Applied Soil Ecology 137: 49-56.
283. Song, L., P. Tian, J. Zhang and G. Jin. 2017. Effects of three years of simulated nitrogen deposition on soil nitrogen dynamics and greenhouse gas emissions in a Korean pine plantation of northeast China. Science of the Total Environment, 609: 1303-1311.
284. Song, M., T. He, H. Chen, K. Wang and D. Li. 2019b. Dynamics of soil gross nitrogen transformations during post-agricultural succession in a subtropical karst region. Geoderma 341: 1-9.
285. Sørensen, P. 2001. Short-term nitrogen transformations in soil amended with animal manure. Soil Biology and Biochemistry 33: 1211-1216.
286. Sotta, E.D., M.D. Corre and E. Veldkamp. 2008. Differing N status and N retention processes of soils under old-growth lowland forest in Eastern Amazonia, Caxiuanã, Brazil. Soil Biology and Biochemistry 40: 740-750.
287. Staelens, J., T. Rütting, D. Huygens, A. De Schrijver, C. Müller, K. Verheyen, et al. 2012. In situ gross nitrogen transformations differ between temperate deciduous and coniferous forest soils. Biogeochemistry 108: 259-277.
288. Stark, C.H., L.M. Condron, M. O'Callaghan, A. Stewart and H.J. Di. 2008. Differences in soil enzyme activities, microbial community structure and short-term nitrogen mineralisation resulting from farm management history and organic matter amendments. Soil Biology and Biochemistry 40: 1352-1363.
289. Stark, S., D.A. Wardle, R. Ohtonen, T. Helle and G.W. Yeates. 2000. The effect of reindeer grazing on decomposition, mineralization and soil biota in a dry oligotrophic Scots pine forest. Oikos 90: 301-310.
290. Stark, S., R. Strömmer and J. Tuomi. 2002. Reindeer grazing and soil microbial processes in two suboceanic and two subcontinental tundra heaths. Oikos 97: 69-78.
291. Sterngren, A.E., S. Hallin and P. Bengtson. 2015. Archaeal ammonia oxidizers dominate in numbers, but bacteria drive gross nitrification in N-amended grassland soil. Frontiers in Microbiology 6: 1350.
292. Stockdale, E., D. Hatch, D. Murphy, S. Ledgard and C. Watson. 2002. Verifying the nitrification to immobilisation ratio (N/I) as a key determinant of potential nitrate loss in grassland and arable soils Agronomie 22(7-8): 831-838.
293. Sun, J., B. Peng, W. Li, G. Qu, W. Dai, G. Dai, et al. 2016. Effects of nitrogen addition on potential soil nitrogen-cycling processes in a temperate forest ecosystem. Soil Science 181: 29-38.

294. Sun, J., W. Dai, B. Peng, J. Liu, T. He, P. Jiang, et al. 2018. Does the accelerated soil N cycling sustain N demand of Quercus mongolica after decade-long elevated CO_2 treatment? Biogeochemistry 139: 197-213.
295. Sun, L., C. Sang, C. Wang, Z. Fan, B. Peng, P. Jiang, et al. 2019a. N_2O production in the organic and mineral horizons of soil had different responses to increasing temperature. Journal of Soils and Sediments 19: 3499-3511.
296. Sun, L., Z. Xia, C. Sang, X. Wang, B. Peng, C. Wang, et al. 2019b. Soil resource status affects the responses of nitrogen processes to changes in temperature and moisture. Biology and Fertility of Soils 55: 629-641.
297. Sun, X., B. Liang, J. Wang, Y. Cheng, S.X. Chang, Z.-C. Cai, et al. 2020. Soil N transformation rates are not linked to fertilizer N losses in vegetable soils with high N input. Soil and Tillage Research 202: 104651.
298. Sun, Y., P.-M. Schleuss, J. Pausch, X. Xu and Y. Kuzyakov. 2018. Nitrogen pools and cycles in Tibetan Kobresia pastures depending on grazing. Biology and Fertility of Soils 54: 569-581.
299. Tahovská, K., M. Choma, E. Kaštovská, F. Oulehle, J. Bárta, H. Šantrůčková, et al. 2020. Positive response of soil microbes to long-term nitrogen input in spruce forest: Results from Gårdsjön whole-catchment N-addition experiment. Soil Biology and Biochemistry 143: 107732.
300. Templer, P.H., W.L. Silver, J. Pett-Ridge, K. M. DeAngelis and M.K. Firestone. 2008. Plant and microbial controls on nitrogen retention and loss in a humid tropical forest. Ecology 89: 3030-3040.
301. Tian, P., J. Zhang, C. Müller, Z. Cai and G. Jin. 2018. Effects of six years of simulated N deposition on gross soil N transformation rates in an old-growth temperate forest. Journal of Forestry Research 29: 647-656.
302. Tietema, A. and W.W. Wessel. 1992. Gross nitrogen transformations in the organic layer of acid forest ecosystems subjected to increased atmospheric nitrogen input. Soil Biology and Biochemistry 24: 943-950.
303. Ting, L., H. Yong and C. Zucong. 2017. Comparison of gross N transformation rates in two paddy soils under aerobic condition. Pedosphere 27: 112-120.
304. Tokuchi, N., S. Yoneda, N. Ohte, N. Usui, K. Koba, M. Kuroiwa, et al. 2014. Seasonal changes and controlling factors of gross N transformation in an evergreen plantation forest in central Japan. Journal of Forest Research 19: 77-85.
305. Troxler, T.G. and D.L. Childers. 2010. Biogeochemical contributions of tree islands to Everglades wetland landscape nitrogen cycling during seasonal inundation. Ecosystems 13: 75-89.
306. Turner, M.G., E.A. Smithwick, K.L. Metzger, D.B. Tinker and W.H. Romme. 2007. Inorganic nitrogen availability after severe stand-replacing fire in the Greater Yellowstone ecosystem. Proceedings of the National Academy of Sciences 104: 4782-4789.
307. Ueda, M.U., P. Kachina, D. Marod, T. Nakashizuka and H. Kurokawa. 2017. Soil properties and gross nitrogen dynamics in old growth and secondary forest in four types of tropical forest in Thailand. Forest Ecology and Management 398: 130-139.
308. Unkovich, M., N. Jamieson, R. Monaghan and D. Barraclough. 1998. Nitrogen mineralisation and plant nitrogen acquisition in a nitrogen-limited calcareous grassland. Environmental and Experimental Botany 40: 209-219.

309. Urakawa, R., H. Shibata, M. Kuroiwa, Y. Inagaki, R. Tateno, T. Hishi, et al. 2014. Effects of freeze–thaw cycles resulting from winter climate change on soil nitrogen cycling in ten temperate forest ecosystems throughout the Japanese archipelago. Soil Biology and Biochemistry 74: 82-94.
310. Urakawa, R., N. Ohte, H. Shibata, K. Isobe, R. Tateno, T. Oda, T. Hishi, K. Fukushima, Y. Inagaki and K. Hirai. 2016. Factors contributing to soil nitrogen mineralization and nitrification rates of forest soils in the Japanese archipelago. Forest Ecology and Management 361: 382-396.
311. Vázquez, E., M. Benito, M. Navas, R. Espejo, E. Díaz-Pinés and N. Teutscherova. 2019. The interactive effect of no-tillage and liming on gross N transformation rates during the summer fallow in an acid Mediterranean soil. Soil and Tillage Research 194: 104297.
312. Venterea, R.T., P.M. Groffman, L.V. Verchot, A.H. Magill and J.D. Aber. 2004. Gross nitrogen process rates in temperate forest soils exhibiting symptoms of nitrogen saturation. Forest Ecology and Management 196(1): 129-142.
313. Verchot, L., Z. Holmes, L. Mulon, P. Groffman and G. Lovett. 2001. Gross vs net rates of N mineralization and nitrification as indicators of functional differences between forest types. Soil Biology and Biochemistry 33: 1889-1901.
314. Verchot, L.V., P.M. Groffman and D.A. Frank. 2002. Landscape versus ungulate control of gross mineralization and gross nitrification in semi-arid grasslands of Yellowstone National Park. Soil Biology and Biochemistry 34: 1691-1699.
315. Vervaet, H., P. Boeckx, A. Boko, O. Van Cleemput and G. Hofman. 2004. The role of gross and net N transformation processes and NH_4^+ and NO_3^- immobilization in controlling the mineral N pool of a temperate mixed deciduous forest soil. Plant and Soil 264: 349-357.
316. Vieites-Blanco, C. and S.J. González-Prieto. 2018. Effects of Carpobrotus edulis invasion on soil gross N fluxes in rocky coastal habitats. Science of the Total Environment 619: 966-976.
317. Wan, Y., X. Ju, J. Ingwersen, U. Schwarz, C.F. Stange, F. Zhang, et al. 2009. Gross nitrogen transformations and related nitrous oxide emissions in an intensively used calcareous soil. Soil Science Society of America Journal 73: 102-112.
318. Wang, C., N. Wang, J. Zhu, Y. Liu, X. Xu, S. Niu, et al. 2018. Soil gross N ammonification and nitrification from tropical to temperate forests in eastern China. Functional Ecology 32: 83-94.
319. Wang, C., Z. Chen, S. Unteregelsbacher, H. Lu, S. Gschwendtner, R. Gasche, et al. 2016. Climate change amplifies gross nitrogen turnover in montane grasslands of Central Europe in both summer and winter seasons. Global Change Biology 22(9): 2963-2978.
320. Wang, J., Q. Liu, J. Zhang and Z. Cai. 2015. Conversion of forest to agricultural land affects the relative contribution of bacteria and fungi to nitrification in humid subtropical soils. Acta Agriculturae Scandinavica, Section B—Soil & Plant Science 65(1): 83-88.
321. Wang, J., N. Sun, M. Xu, S. Wang, J. Zhang, Z. Cai and Y. Cheng. 2019. The influence of long-term animal manure and crop residue application on abiotic and biotic N immobilization in an acidified agricultural soil. Geoderma 337: 710-717.
322. Wang, J., Y. Cheng, J.-b. Zhang, C. Müller and Z.-c. Cai. 2016. Soil gross nitrogen transformations along a secondary succession transect in the north subtropical forest ecosystem of southwest China. Geoderma 280: 88-95.

323. Wang, J., Y. Cheng, Y. Jiang, B. Sun, J. Fan, J. Zhang, et al. 2017b. Effects of 14 years of repeated pig manure application on gross nitrogen transformation in an upland red soil in China. Plant and Soil 415: 161-173.
324. Wang, J., B. Zhang, Y. Tian, H. Zhang, Y. Cheng and J. Zhang. 2018. A soil management strategy for ameliorating soil acidification and reducing nitrification in tea plantations. European Journal of Soil Biology 88: 36-40.
325. Wang, J., J. Zhang, C. Müller and Z. Cai. 2017a. The Mechanisms of High N_2O Emissions from Greenhouse Vegetable Field Soils. CLEAN–Soil, Air, Water, 45(10): 1600210.
326. Wang, J., J. Zhang, C. Müller, Z. Cai. 2017c. Temperature sensitivity of gross N transformation rates in an alpine meadow on the Qinghai-Tibetan Plateau. Journal of Soils and Sediments, 17(2): 423–431.
327. Wang, X., Huang, D. and Wang, Q., 2020. Organic farming promotes selective uptake of glycine over nitrate uptake by pakchoi. Soil Science and Plant Nutrition 66(3): 438-448.
328. Wang, Y., Z. Xu, J. Zheng, K.M. Abdullah and Q. Zhou. 2015. $\delta^{15}N$ of soil nitrogen pools and their dynamics under decomposing leaf litters in a suburban native forest subject to repeated prescribed burning in southeast Queensland, Australia. Journal of Soils and Sediments 15: 1063-1074.
329. Watson, C., G. Travers, D. Kilpatrick, A. Laidlaw and E. O'Riordan. 2000. Overestimation of gross N transformation rates in grassland soils due to non-uniform exploitation of applied and native pools. Soil Biology and Biochemistry 32: 2019-2030.
330. Weitzman, J.N. and J.P. Kaye. 2016. Variability in soil nitrogen retention across forest, urban, and agricultural land uses. Ecosystems 19: 1345-1361.
331. Weitzman, J. N., P. M. Groffman, J. L. Campbell, C. T. Driscoll, R. T. Fahey, T. J. Fahey, et al. 2020. Ecosystem nitrogen response to a simulated ice storm in a northern hardwood forest. Ecosystems: 23(6), 1186-1205.
332. Wei-Wei, L., S. Riya, Z. Sheng, M. Hosomi, H.-L. Zhang and S. Wei-Ming. 2012. In situ dissimilatory nitrate reduction to ammonium in a paddy soil fertilized with liquid cattle waste. Pedosphere: 22(3): 314-321.
333. Wertz, S., C. Goyer, D.L. Burton, B.J. Zebarth and M.H. Chantigny. 2018. Processes contributing to nitrite accumulation and concomitant N_2O emissions in frozen soils. Soil Biology and Biochemistry 126: 31-39.
334. Wessel, W.W. and A. Tietema. 1992. Calculating gross N transformation rates of ^{15}N pool dilution experiments with acid forest litter: analytical and numerical approaches. Soil Biology and Biochemistry 24: 931-942.
335. West, J.B., S.E. Hobbie and P.B. Reich. 2006. Effects of plant species diversity, atmospheric [CO_2], and N addition on gross rates of inorganic N release from soil organic matter. Global Change Biology 12: 1400-1408.
336. Westbrook, C.J. and K.J. Devito. 2004. Gross nitrogen transformations in soils from uncut and cut boreal upland and peatland coniferous forest stands. Biogeochemistry 68: 33-50.
337. Wild, B., J. Schnecker, A. Knoltsch, M. Takriti, M. Mooshammer, N. Gentsch, et al. 2015. Microbial nitrogen dynamics in organic and mineral soil horizons along a latitudinal transect in western Siberia. Global Biogeochemical Cycles 29: 567-582.
338. Wild, B., P. Ambus, S. Reinsch and A. Richter. 2018. Resistance of soil protein depolymerization rates to eight years of elevated CO_2, warming, and summer drought in a temperate heathland. Biogeochemistry 140: 255-267.

339. Wild, B., S. Alaei, P. Bengtson, S. Bodé, P. Boeckx, J. Schnecker, et al. 2017. Short-term carbon input increases microbial nitrogen demand, but not microbial nitrogen mining, in a set of boreal forest soils. Biogeochemistry 136: 261-278.
340. Williams, M.A., C.W. Rice and C.E. Owensby. 2001. Nitrogen competition in a tallgrass prairie ecosystem exposed to elevated carbon dioxide. Soil Science Society of America Journal 65: 340-346.
341. Wu, H., M. Dannenmann, B. Wolf, X. Han, X. Zheng and K. Butterbach-Bahl. 2012. Seasonality of soil microbial nitrogen turnover in continental steppe soils of Inner Mongolia. Ecosphere 3: 1-18.
342. Wu, H., Q. Li, C. Lu, L. Zhang, J. Zhu, F.A. Dijkstra, et al. 2016. Elevated ozone effects on soil nitrogen cycling differ among wheat cultivars. Applied Soil Ecology 108: 187-194.
343. Xia, Q., L. Chen, W. Xiang, S. Ouyang, H. Wu, P. Lei, et al. 2021. Increase of soil nitrogen availability and recycling with stand age of Chinese-fir plantations. Forest Ecology and Management 480: 118643.
344. Xie, Y., J. Zhang, L. Meng, C. Müller and Z. Cai. 2015. Variations of soil N transformation and N_2O emissions in tropical secondary forests along an aridity gradient. Journal of Soils and Sediments 15: 1538-1548.
345. Xie, Y., L. Yang, T. Zhu, H. Yang, J. Zhang, J. Yang, et al. 2018. Rapid recovery of nitrogen retention capacity in a subtropical acidic soil following afforestation. Soil Biology and Biochemistry 120: 171-180.
346. Xu, Y., J. Li and X. Yin. 2020. Continuous Cropping Affects Gross Nitrogen Transformations in Subtropical Acidic Soils Under Greenhouse Cultivation. Journal of Soil Science and Plant Nutrition: 1-9.
347. Xu, Y., W. Qiu, J. Sun, C. Müller and B. Lei. 2019. Effects of wheat/faba bean intercropping on soil nitrogen transformation processes. Journal of Soils and Sediments 19: 1724-1734.
348. Xu, Yongbo and Xu Zhihong (2015). Effects of land use change on soil gross nitrogen transformation rates in subtropical acid soils of Southwest China. Environmental Science and Pollution Research 22(14): 10850–10860.
349. Yang, W.H., R.A. Ryals, D.F. Cusack and W.L. Silver. 2017. Cross-biome assessment of gross soil nitrogen cycling in California ecosystems. Soil Biology and Biochemistry 107: 144-155.
350. Yokobe, T., F. Hyodo and N. Tokuchi. 2018. Seasonal effects on microbial community structure and nitrogen dynamics in temperate forest soil. Forests 9: 153.
351. Yokobe, T., F. Hyodo and N. Tokuchi. 2020. Volcanic deposits affect soil nitrogen dynamics and fungal–bacterial dominance in temperate forests. Soil Biology and Biochemistry 150: 108011.
352. Yu, L., R. Kang, J. Mulder, J. Zhu and P. Dörsch. 2017. Distinct fates of atmogenic NH_4^+ and NO_3^- in subtropical, N-saturated forest soils. Biogeochemistry 133: 279-294.
353. Zak, D.R., W.E. Holmes, M.J. Tomlinson, K.S. Pregitzer and A.J. Burton. 2006. Microbial cycling of C and N in northern hardwood forests receiving chronic atmospheric NO_3^- deposition. Ecosystems 9: 242-253.
354. Zaman, M. and S. Chang. 2004. Substrate type, temperature, and moisture content affect gross and net N mineralization and nitrification rates in agroforestry systems. Biology and Fertility of Soils 39: 269-279.

355. Zaman, M., H. Di and K. Cameron. 1999a. A field study of gross rates of N mineralization and nitrification and their relationships to microbial biomass and enzyme activities in soils treated with dairy effluent and ammonium fertilizer. Soil Use and Management 15: 188-194.
356. Zaman, M., H. Di, K. Cameron and C. Frampton. 1999b. Gross nitrogen mineralization and nitrification rates and their relationships to enzyme activities and the soil microbial biomass in soils treated with dairy shed effluent and ammonium fertilizer at different water potentials. Biology and Fertility of Soils 29: 178-186.
357. Zaman, M., M. Matsushima, S. Chang, K. Inubushi, L. Nguyen, S. Goto, et al. 2004. Nitrogen mineralization, N_2O production and soil microbiological properties as affected by long-term applications of sewage sludge composts. Biology and Fertility of Soils 40: 101-109.
358. Zeller, B., S. Recous, M. Kunze, J. Moukoumi, M. Colin-Belgrand, S. Bienaimé, et al. 2007. Influence of tree species on gross and net N transformations in forest soils. Annals of Forest Science 64: 151-158.
359. Zeng, Y., W. Xiang, X. Deng, X. Fang, C. Liu and C. Peng. 2014. Soil N forms and gross transformation rates in Chinese subtropical forests dominated by different tree species. Plant and Soil 384: 231-242.
360. Zhang, J., J. Wang, C. Müller and Z. Cai. 2016a. Ecological and practical significances of crop species preferential N uptake matching with soil N dynamics. Soil Biology and Biochemistry 103: 63-70.
361. Zhang, J., T. Lan, C. Müller and Z. Cai. 2015. Dissimilatory nitrate reduction to ammonium (DNRA) plays an important role in soil nitrogen conservation in neutral and alkaline but not acidic rice soil. Journal of Soils and Sediments 15(3): 523-531.
362. Zhang, J., C. Müller, T. Zhu, Y. Cheng and Z. Cai. 2011a. Heterotrophic nitrification is the predominant NO_3^- production mechanism in coniferous but not broad-leaf acid forest soil in subtropical China. Biology and Fertility of Soils 47(5): 533.
363. Zhang, J., P. Tian, J. Tang, L. Yuan, Y. Ke, Z. Cai, et al. 2016b. The characteristics of soil N transformations regulate the composition of hydrologic N export from terrestrial ecosystem. Journal of Geophysical Research: Biogeosciences 121: 1409-1419.
364. Zhang, J., T. Zhu, Z. Cai and C. Müller. 2011b. Nitrogen cycling in forest soils across climate gradients in Eastern China. Plant and Soil 342: 419-432.
365. Zhang, J., T. Zhu, Z. Cai, S. Qin and C. Müller. 2012a. Effects of long-term repeated mineral and organic fertilizer applications on soil nitrogen transformations. European Journal of Soil Science 63: 75-85.
366. Zhang, J., Z. Cai, W. Yang, T. Zhu, Y. Yu, X. Yan, et al. 2012b. Long-term field fertilization affects soil nitrogen transformations in a rice-wheat-rotation cropping system. Journal of Plant Nutrition and Soil Science 175: 939-946.
367. Zhang, J., T. Zhu, T. Meng, Y. Zhang, J. Yang, W. Yang, C. Müller and Z. Cai. 2013b. Agricultural land use affects nitrate production and conservation in humid subtropical soils in China. Soil Biology and Biochemistry 62: 107-114.
368. Zhang, J.-b., Z.-c. Cai, T.-b. Zhu, W.-y. Yang and C. Müller. 2013a. Mechanisms for the retention of inorganic N in acidic forest soils of southern China. Scientific Reports 3: 2342.
369. Zhang, S., Q. Zheng, L. Noll, Y. Hu and W. Wanek. 2019. Environmental effects on soil microbial nitrogen use efficiency are controlled by allocation of organic nitrogen to

microbial growth and regulate gross N mineralization. Soil Biology and Biochemistry 135: 304-315.

370. Zhang, Y., F. Wang, J. Zhang, T. Zhu, C. Lin, C. Müller, et al. 2015. Cattle manure and straw have contrasting effects on organic nitrogen mineralization pathways in a subtropical paddy soil. Acta Agriculturae Scandinavica, Section B—Soil & Plant Science 65: 619-628.
371. Zhang, Y., H. Ding, X. Zheng, Z. Cai, T. Misselbrook, A. Carswell, et al. 2018a. Soil N transformation mechanisms can effectively conserve N in soil under saturated conditions compared to unsaturated conditions in subtropical China. Biology and Fertility of Soils 54: 495-507.
372. Zhang, Y., J. Zhang, T. Zhu, C. Müller and Z. Cai. 2015. Effect of orchard age on soil nitrogen transformation in subtropical China and implications. Journal of Environmental Sciences 34: 10-19.
373. Zhang, Y., S. Liu, Y. Cheng, Z. Cai, C. Müller and J. Zhang. 2019a. Composition of soil recalcitrant C regulates nitrification rates in acidic soils. Geoderma 337: 965-972.
374. Zhang, Y., S. Dai, X. Huang, Y. Zhao, J. Zhao, Y. Cheng, et al. 2020a. pH-induced changes in fungal abundance and composition affects soil heterotrophic nitrification after 30 days of artificial pH manipulation. Geoderma 366: 114255.
375. Zhang, Y., J. Zhang, T. Meng, T. Zhu, C. Müller and Z. Cai. 2013. Heterotrophic nitrification is the predominant NO_3^- production pathway in acid coniferous forest soil in subtropical China. Biology and Fertility of Soils 49(7): 955-957.
376. Zhang, Y., W. Zhao, Z. Cai, C. Müller and J. Zhang. 2018b. Heterotrophic nitrification is responsible for large rates of N_2O emission from subtropical acid forest soil in China. European Journal of Soil Science 69(4): 646-654.
377. Zhang, Y., X. Zheng, B. Guo, J. Yu, A. Carswell, T. Misselbrook, et al. 2020b. Mechanisms behind the inhibition of autotrophic nitrification following rice-straw incorporation in a subtropical acid soil. Soil and Tillage Research 196: 104436.
378. Zhang, Y., X. Zheng, X. Ren, J. Zhang, T. Misselbrook, L. Cardenas, et al. 2019b. Land-use type affects nitrate production and consumption pathways in subtropical acidic soils. Geoderma 337: 22-31.
379. Zhao, N. and X.G. Li. 2017. Effects of aspect–vegetation complex on soil nitrogen mineralization and microbial activity on the Tibetan Plateau. Catena 155: 1-9.
380. Zhao, W., J. Zhang, C. Müller and Z. Cai. 2017. Mechanisms behind the stimulation of nitrification by N input in subtropical acid forest soil. Journal of Soils and Sediments 17: 2338-2345.
381. Zhao, W., J.-b. Zhang, C. Müller and Z.-c. Cai. 2018. Effects of pH and mineralisation on nitrification in a subtropical acid forest soil. Soil Research 56: 275-283.
382. Zhao, Y., J. Zhang, C. Müller and Z. Cai. 2018b. Temporal variations of crop residue effects on soil N transformation depend on soil properties as well as residue qualities. Biology and Fertility of Soils 54: 659-669.
383. Zhao, Y.; Wang, Jing; Cai, Zucong; Müller, Christoph; Zhang, Jinbo. 2018a. Short-term effects of nitrapyrin, rice straw and its biochar application on N transformation in soils of humid subtropical China. Acta Agriculturae Scandinavica, Section B — Soil & Plant Science 68(5): 448-456
384. Zheng, X., C. Lin, B. Guo, J. Yu, H. Ding, S. Peng, et al. 2020. Mechanisms behind soil N dynamics following cover restoration in degraded land in subtropical China. Journal of Soils and Sediments 20: 1897-1905.

385. Zhou, M., B. Zhu, N. Brüggemann, M. Dannenmann, Y. Wang and K. Butterbach-Bahl. 2018. Sustaining crop productivity while reducing environmental nitrogen losses in the subtropical wheat-maize cropping systems: A comprehensive case study of nitrogen cycling and balance. Agriculture, Ecosystems & Environment 231: 1-14.
386. Zhu, B., J.L. Gutknecht, D.J. Herman, D.C. Keck, M.K. Firestone and W. Cheng. 2014. Rhizosphere priming effects on soil carbon and nitrogen mineralization. Soil Biology and Biochemistry 76: 183-192.
387. Zhu, G., X. Ju, J. Zhang, C. Müller, R.M. Rees, R.E. Thorman, et al. 2019a. Effects of the nitrification inhibitor DMPP (3, 4-dimethylpyrazole phosphate) on gross N transformation rates and N_2O emissions. Biology and Fertility of Soils 55: 603-615.
388. Zhu, G., X. Song, X. Ju, J. Zhang, C. Müller, R. Sylvester-Bradley, et al. 2019b. Gross N transformation rates and related N_2O emissions in Chinese and UK agricultural soils. Science of the Total Environment 666: 176-186.
389. Zhu, J., J. Mulder, L. Bakken and P. Dörsch. 2013. The importance of denitrification for N_2O emissions from an N-saturated forest in SW China: results from in situ ^{15}N labeling experiments. Biogeochemistry 116(1-3): 103-117.
390. Zhu, K., S. Bruun and L.S. Jensen. 2016. Nitrogen transformations in and N_2O emissions from soil amended with manure solids and nitrification inhibitor. European Journal of Soil Science 67(6): 792-803.
391. Zhu, T., C. Yang, J. Wang, S. Zeng, M. Liu, J. Yang, et al. 2018. Bacterivore nematodes stimulate soil gross N transformation rates depending on their species. Biology and Fertility of Soils 54: 107-118.
392. Zhu, T., J. Zhang, T. Meng, Y. Zhang, J. Yang, C. Müller, et al. 2014. Tea plantation destroys soil retention of NO_3^- and increases N_2O emissions in subtropical China. Soil Biology and Biochemistry 73: 106-114.
393. Zhu, T., J. Zhang, Z. Cai and C. Müller. 2011. The N transformation mechanisms for rapid nitrate accumulation in soils under intensive vegetable cultivation. Journal of Soils and Sediments 11: 1178.
394. Zhu, T., T. Meng, J. Zhang, W. Zhong, C. Müller and Z. Cai. 2015. Fungi-dominant heterotrophic nitrification in a subtropical forest soil of China. Journal of Soils and Sediments 15(3): 705-709.
395. Zhu, T., Q. Dang, J. Zhang, C. Müller and Z. Cai. 2014. Reductive soil disinfestation (RSD) alters gross N transformation rates and reduces NO and N_2O emissions in degraded vegetable soils. Plant and Soil 382: 269-280.
396. Zhu, T., T. Meng, J. Zhang, Y. Yin, Z. Cai, W. Yang, et al. 2013. Nitrogen mineralization, immobilization turnover, heterotrophic nitrification, and microbial groups in acid forest soils of subtropical China. Biology and Fertility of Soils 49: 323-331.
397. Zhu, T. S. Zeng, H. Qin, K. Zhou, H. Yang, Lan, F. Huang, J. Cao and C. Müller. 2016. Low nitrate retention capacity in calcareous F., soil under woodland in the karst region of southwestern China. Soil Biology and Biochemistry 97: 99-101.
398. Zhu, T., J. Zhang, W.Yang and Z. Cai. 2013. Effects of organic material amendment and water content on NO, N_2O, and N_2 emissions in a nitrate-rich vegetable soil. Biology and Fertility of Soils 49(2): 153-163.
