# Supplementary material for: Expanding agroforestry can increase nitrate retention and mitigate the global impact of a leaky nitrogen cycle in croplands
Source: Nat Food. 2022 Dec 28;4(1):109–21. doi: 10.1038/s43016-022-00657-x (PMC10154242; doi:10.1038/s43016-022-00657-x)
Supplement: Supplementary file 2 — Reporting Summary [file 43016_2022_657_MOESM2_ESM.pdf]

nature research

Corresponding author(s): Prof. Yi Cheng
School of Geography, Nanjing Normal University, Nanjing 210023, China
ycheng@njnu.edu.cn

Last updated by author(s): Oct 29, 2022

Reporting Summary

Nature Research wishes to improve the reproducibility of the work that we publish. This form provides structure for consistency and transparency in reporting. For further information on Nature Research policies, see our Editorial Policies and the Editorial Policy Checklist.

Statistics

For all statistical analyses, confirm that the following items are present in the figure legend, table legend, main text, or Methods section.

n/a	Confirmed	
☐	☒	The exact sample size (*n*) for each experimental group/condition, given as a discrete number and unit of measurement
☐	☒	A statement on whether measurements were taken from distinct samples or whether the same sample was measured repeatedly
☐	☒	The statistical test(s) used AND whether they are one- or two-sided<br>*Only common tests should be described solely by name; describe more complex techniques in the Methods section.*
☒	☐	A description of all covariates tested
☐	☒	A description of any assumptions or corrections, such as tests of normality and adjustment for multiple comparisons
☐	☒	A full description of the statistical parameters including central tendency (e.g. means) or other basic estimates (e.g. regression coefficient) AND variation (e.g. standard deviation) or associated estimates of uncertainty (e.g. confidence intervals)
☐	☒	For null hypothesis testing, the test statistic (e.g. *F*, *t*, *r*) with confidence intervals, effect sizes, degrees of freedom and *P* value noted<br>*Give P values as exact values whenever suitable.*
☒	☐	For Bayesian analysis, information on the choice of priors and Markov chain Monte Carlo settings
☒	☐	For hierarchical and complex designs, identification of the appropriate level for tests and full reporting of outcomes
☒	☐	Estimates of effect sizes (e.g. Cohen's *d*, Pearson's *r*), indicating how they were calculated

Our web collection on statistics for biologists contains articles on many of the points above.

Software and code

Policy information about availability of computer code

Data collection	The results contained in graphs were generated by using GetData (version 2.22).
Data analysis	The R (R-4.1.2) codes used to generate the results and figures reported in this study are available at Figshare (https://doi.org/10.6084/m9.figshare.21406731.v4).

For manuscripts utilizing custom algorithms or software that are central to the research but not yet described in published literature, software must be made available to editors and reviewers. We strongly encourage code deposition in a community repository (e.g. GitHub). See the Nature Research guidelines for submitting code & software for further information.

Data

Policy information about availability of data

All manuscripts must include a data availability statement. This statement should provide the following information, where applicable:

- Accession codes, unique identifiers, or web links for publicly available datasets
- A list of figures that have associated raw data
- A description of any restrictions on data availability

The data supporting the findings of this study are available in Supplementary Dataset 1 and 2. The source data underlying Figs. 1, 4, 5, and 6 are provided as a Source Data file, however the source data underlying Figs. 2 and 3 are available at Figshare (https://doi.org/10.6084/m9.figshare.21406731.v4).

Field-specific reporting

Please select the one below that is the best fit for your research. If you are not sure, read the appropriate sections before making your selection.

☐ Life sciences ☐ Behavioural & social sciences ☒ Ecological, evolutionary & environmental sciences

For a reference copy of the document with all sections, see nature.com/documents/nr-reporting-summary-flat.pdf

Ecological, evolutionary & environmental sciences study design

All studies must disclose on these points even when the disclosure is negative.

Study description

We compiled 4032 observations from 398 published 115N pool dilution and tracing studies to predict soil internal soil N cycle patterns and their environmental consequences. Detailed site such as longitude, latitude, climatic zone, ecosystem type, mean annual temperature (MAT), mean annual precipitation (MAP), total C, total N, C:N, soil pH, microbial biomass C and N, the abundances of bacteria, ammonia-oxidizing archaea, ammonia-oxidizing bacteria, and fungi, fungi to bacteria ratio, and extractable ammonium N and Nitrate N were collected along with soil gross N transformation rates gross N mineralization, gross nitrification, gross autotrophic nitrification, gross heterotrophic nitrification, gross N immobilization, gross ammonium immobilization, gross nitrate immobilization, and dissimilatory nitrate reduction to ammonium). The data on the emission of N2O were also collected from original articles. The ratios of gross autotrophic nitrification to gross ammonium immobilization, gross autotrophic nitrification to gross N mineralization, and nitrate to ammonium were calculated and included in the analysis. We also calculated the net ammonium production and net nitrate production. Data from organic, mineral, and mixed (organic + mineral) soil horizons were used to analyze the global-scale pattern in the data; however, the comparisons between different ecosystem types were limited to data from mineral soil layers. In large-scale pattern analysis, measurements from disturbed and intact soils were included, but the comparisons between different ecosystem types were limited to measurements from disturbed soils. Most of the collected studies were conducted under laboratory incubation under aerobic conditions. The dataset included three terrestrial ecosystems: forests (58%), grasslands (15%), and croplands (25%). We coded climatic zones as humid subtropical, tropical wet, the Mediterranean, continental, and marine west coast based on the Köppen Classification System.
We first calculated the average gross N transformation rates across ecosystem types, and analyzed global-scale patterns in the data by regression analysis. Second, we predicted the global distribution of soil gross N transformation rates by five machine-learning models using a global database of soil and climatic variables. Third, we conducted structural equation modelling (SEM) to estimate the factors directly and indirectly control soil N cycling. Finally, we calculated the ratios of gross autotrophic nitrification to gross ammonium immobilization and nitrate to ammonium , and used mixed-effects meta-regression models to explore the most important factors affecting these ratios. These ratios are used as indicators of the potential risk of N loss. Soils with a high ratios have greater potential of N loss than those with low ratios.
We found that total nitrate consumption represents 49% of the total nitrate production globally with a high ratio of autotrophic nitrification to ammonium immobilization (1.71±0.31), manifesting a leaky N cycle. We observed high spatial variations in the global N cycle as its pattern changes from a conservative cycle in forests to a less conservative one in grasslands and a leaky one in croplands, as indicated by the increasing ratios of autotrophic nitrification to ammonium immobilization and nitrate to ammonium. The structural equation modelling revealed that soil properties (soil pH, total N and carbon to N ratio) were more important in shaping the internal N cycle than climate. We suggest that the global N cycle requires a shift towards agroforestry systems and a possible increase of nitrate retention in croplands, which would play a vital role in ecological restoration.

Research sample

We systematically searched all peer-reviewed papers published prior to December 2020 that examined soil gross N transformation rates using the Web of Science and Google Scholar Database and searched for references within these papers. Our search also included studies summarized in previously published gross N transformation rates meta-analyses. We utilized the following terms: 'gross nitrogen rates'; 'soil gross nitrogen transformation'; 'gross nitrogen mineralization'; 'gross nitrification'; 'gross nitrogen immobilization'; or 'gross dissimilatory nitrate reduction to ammonium' to search for papers. We employed the following criteria for compiling gross N transformation rate data: 1) gross N transformation rates were estimated using the topsoil samples (0-20 cm), 2) Most of the incubation periods for gross N transformation rates range from 24 to 48 h, and 3) Gross N transformation rates data were quantified based on the 15N isotopic pool dilution technique and tracing model. In total, 398 studies met these criteria.

Sampling strategy

We followed the guidelines of PRISMA (Preferred Reporting Items for Systematic Reviews and Meta-Analyses) to perform the literature search. We employed the following criteria for compiling gross N transformation rate data: 1) gross N transformation rates were estimated using the topsoil samples (0-20 cm), 2) Most of the incubation periods for gross N transformation rates range from 24 to 48 h, and 3) Gross N transformation rates data were quantified based on the 15N isotopic pool dilution technique and tracing model. In total, 398 studies met these criteria.

Data collection

We systematically searched all peer-reviewed papers published prior to December 2020 that examined soil gross N transformation rates using the Web of Science and Google Scholar Database and searched for references within these papers. Our search also included studies summarized in previously published gross N transformation rates meta-analyses. We utilized the following terms: 'gross nitrogen rates'; 'soil gross nitrogen transformation'; 'gross nitrogen mineralization'; 'gross nitrification'; 'gross nitrogen immobilization'; or 'gross dissimilatory nitrate reduction to ammonium' to search for papers.

Timing and spatial scale

We systematically searched all peer-reviewed papers published prior to December 2020 that examined soil gross N transformation rates.

Data exclusions

For the meta-analysis dataset, the studies which didn't follow the criteria described in "Sampling strategy" were excluded into analysis.

Reproducibility

All attempts to repeat the experiment were successful.

Randomization	Not applicable
Blinding	Blinding is not relevant to our study. Our study is a meta analysis regarding the global soil nitrogen cycle.

Did the study involve field work? ☐ Yes ☒ No

Reporting for specific materials, systems and methods

We require information from authors about some types of materials, experimental systems and methods used in many studies. Here, indicate whether each material, system or method listed is relevant to your study. If you are not sure if a list item applies to your research, read the appropriate section before selecting a response.

Materials & experimental systems

n/a	Involved in the study
☒	☐ Antibodies
☒	☐ Eukaryotic cell lines
☒	☐ Palaeontology and archaeology
☒	☐ Animals and other organisms
☒	☐ Human research participants
☒	☐ Clinical data
☒	☐ Dual use research of concern

Methods

n/a	Involved in the study
☒	☐ ChIP-seq
☒	☐ Flow cytometry
☒	☐ MRI-based neuroimaging
